# Enhancing medical intern competence in urethral catheterization: Impact of simulation-based training on knowledge, self-efficacy, and clinical outcomes

**DOI:** 10.1371/journal.pone.0353677

**Published:** 2026-07-17

**Authors:** Maher Abdessater, Anthony Kanbar, Joey El Khoury, Rami Halabi, Ramy Touma Sawaya, Raghid El Khoury

**Affiliations:** 1 Doctoral College, Holy Spirit University of Kaslik (USEK), Jounieh, Lebanon; 2 Division of Urology, Department of Surgery, American University of Beirut Medical Center, Beirut, Lebanon; 3 School of medicine and medical sciences, Holy Spirit University of Kaslik (USEK), Jounieh, Lebanon; Jan Biziel University Hospital No 2 in Bydgoszcz: Szpital Uniwersytecki Nr 2 im dr Jana Biziela w Bydgoszczy, POLAND

## Abstract

**Introduction:**

Urethral catheterization is a common procedure, yet most related complications occur when performed by new medical interns. Studies have shown inadequate training and knowledge among junior doctors. This study evaluated the declarative knowledge of new interns regarding urethral catheterization and assessed the effectiveness of a simulation-based urology session in improving their knowledge, confidence, and perceived self-efficacy.

**Methods:**

A one-day urology simulation workshop was conducted for 40 new interns at a university hospital. Participants completed a pre-session online questionnaire assessing demographics, urinary catheterization knowledge, and self-reported confidence and self-efficacy. The workshop included three modules: an interactive presentation, instructional videos, and hands-on simulation on male and female models. Post-session assessments measured changes in knowledge, confidence, and self-efficacy scores using standardized evaluation tools.

**Results:**

Forty new medical interns participated. Only 15% felt adequately prepared before training. Knowledge gaps were identified regarding catheter types and sizes (92.5%), optimal patient positioning (62%), proper meatus exposure, and balloon inflation technique (100%). Following the workshop, mean knowledge scores increased from 7.7 ± 1.24 to 12.2 ± 1.11, self-reported confidence from 6.60 ± 1.79 to 8.66 ± 0.94, and perceived self-efficacy from 3.32 ± 0.94 to 4.32 ± 0.62 (all p < 0.001). At six-month follow-up, participants collectively performed 274 urethral catheterizations with a 93% success rate and a low complication rate of 4% (n = 11), reflecting effective transfer of learning from the simulation to clinical practice.

**Discussion:**

New medical interns demonstrated insufficient baseline knowledge of urethral catheterization. The structured urology simulation session significantly improved their declarative knowledge, confidence, and perceived self-efficacy, underscoring the value of simulation-based training in early postgraduate medical education.

## Background

Urethral catheterization (UC) is routinely performed in hospitalized patients [1]. During this procedure, a catheter is inserted into the urethra and advanced through the striated urinary sphincter and bladder neck into the bladder to drain urine. If there is a need for continuous urine drainage, a balloon surrounding the tip of the catheter is inflated in the bladder to hold the catheter proximal to the bladder neck [2]. UC is performed for therapeutic purposes, such as urinary retention [3], bladder emptying or urine output measurement perioperatively or in acutely ill patients [3,4], urinary incontinence, drug delivery, bladder irrigation [5], social and hygiene reasons [3], or diagnostic purposes, such as urodynamic studies, cystogram imaging, collection of urine sample [5]. Apart from clean intermittent self-catheterization, UC is a sterile procedure that requires special knowledge to assess the indications, contraindications, and potential complications, and specific technical skills to expose the urethral meatus properly and align the urethra correctly during the maneuver [6].

Successful UC is operator dependent. Early detection of risk factors for complications in males, such as enlarged prostate and urethral strictures, allows for correct decision-making to prevent UC-related morbidity [7]. Similarly in women, special skills and maneuvers are required when risk factors for difficult catheterization are present, such as morbid obesity, intravaginal retraction of the urethral meatus, and prolapse, to optimize the visualization and access the introitus and urethral meatus [8,9]. Iatrogenic urethral trauma caused by medical intervention occurs in 0.3% of catheterization maneuvers during the insertion process, and is mainly caused by false passage creation or intraurethral balloon inflation and may lead to short-term and long-term morbidities [10,11]. Complications from UC increase hospital stay by 9.4 ± 10 days and costs by €335,377 over 6 months [10]. Recent efforts are focusing on the identification of strategies to reduce inappropriate catheter use and avoid associated complications in the short-term, such as infection, hematuria, obstructive kidney injury, need for transfusion or cystoscopy, and long-term, such as urethral stricture, need for long-term bladder catheter or self-urethral dilation [12–16].

At the university hospital, UC is performed traditionally by physicians. Newly qualified doctors are expected to safely and confidently perform UC at the end of their training [1]. However, recent literature shows that junior doctors receive inadequate theoretical and practical training concerning UC during their years in medical school [7,17] before their direct involvement in patient care. The rate of medical interns who did not feel prepared to perform this skill varied in the literature between 18.6% and 55.6% among the Irish [7], Scottish [18], French [19], American [20], and Filipino [17] experiences. Most catheterization-related morbidities occur when interns perform UC, especially at the beginning of their internship and when they are unsupervised [1,7]. Gaps were reported in the theoretical knowledge of the steps of UC, and the mechanisms and identification of iatrogenic injuries [17]. On the practical side, nearly half of new interns have not undergone a urology rotation during their undergraduate training [21,22] and have never performed UC. The majority reported a lack of previously supervised catheterization and especially in female patients [21].

UC training programs successfully improved the knowledge of new interns and their confidence level in performing the skill [23,24] and reduced the incidence of iatrogenic injuries. Still, the real benefit of simulation-based training in UC is debatable because the current models may lack the typical challenges encountered in actual patients, such as increased pain, difficult entry to the bladder, and anatomical variations.

Therefore, as part of a plan to reduce catheter-related morbidity in practice, this study was conducted to evaluate new interns’ declarative knowledge regarding UC and assess the effectiveness of a urology simulation session in improving their declarative knowledge score, perceived self-efficacy (PSE), and technical skills.

## Methods

This study employed a prospective single-group pre–post educational design to evaluate the impact of a simulation-based urethral catheterization workshop on interns’ knowledge, technical performance, and perceived self-efficacy.

### Participant recruitment

The urology simulation session was conducted for new interns at the University Hospital. This was done at the beginning of their academic year in order to adequately train the interns on proper, safe, and comfortable technique. Inclusion criteria for the study mandated that all participants were first-year interns who graduated from the affiliated medical school the previous year. Furthermore, all participants were not previously part of a urology training or simulation session and had no extra specialized urology-related training aside from their rotations during undergraduate training. All participants had the study thoroughly explained to them and were asked to sign an informed consent prior to enrollment.

### Pre-testing

The urology simulation session was a one-day curriculum organized by the urology department to teach participants about UC. Interns were divided into small groups of eight. The simulation session had 2 theoretical and one practical module of twenty minutes each. Prior to starting the educational modules, all participants were asked to fill out an anonymous, online questionnaire and perform a foley catheter insertion on the available male and female simulation models.

The first section in the questionnaire asked about demographic characteristics such as age, sex, self-reported confidence level on a scale of 1–10, previous attempts at inserting a foley catheter, and opinion on preferred method of training for hands-on procedures. The following section included 2 open ended questions asking participants to give the indications and contraindications for UC, and 13 dichotomous or multiple-choice questions with a single correct answer that were inspired by previous similar works. This section assessed medical knowledge related to catheter insertion, such as the types of available catheters and sizes, optimal place to apply lubricant, angle of penis during insertion, depth of catheter insertion, and importance of drained urine volume after insertion. Self-reported confidence in performing urethral catheterization was measured using a numerical rating scale from 1 to 10, where 10 indicated the highest level of confidence. Perceived self-efficacy was evaluated using a 5-point Likert scale reflecting the participant’s perceived preparedness to perform urethral catheterization. These items were developed for the purpose of this educational evaluation and are consistent with approaches commonly used in simulation-based medical education studies to capture changes in perceived competence following training. Finally, the perceived self-efficiency (PSE) score reflected interns’ beliefs in their competence to perform UC, and involved a self-reflective 5-point Likert scale, for the question “How prepared do you feel to perform UC on a scale from 1 to 5 (where 5 is the highest and 1 is the lowest preparedness levels)?”. The questionnaire was reviewed by the teaching urologist involved in the training program to ensure clarity and relevance to the learning objectives.

The knowledge questionnaire included two open-ended questions assessing recognition of indications and contraindications for urethral catheterization, as well as thirteen multiple-choice or dichotomous questions addressing key procedural and theoretical aspects of catheterization, including catheter types and sizes, patient and operator positioning, sterile technique, exposure of the urethral meatus, lubrication, insertion depth, balloon inflation, and procedural documentation. Each correct response was assigned one point, generating a cumulative declarative knowledge score used for pre- and post-training comparison. The questionnaire items were developed based on previously published educational studies on urethral catheterization and standard urology references, including Campbell-Walsh Urology, to ensure that the content reflected clinically relevant knowledge and procedural steps expected from new medical interns.The first attempt of foley insertion was done under supervision of a senior resident, who utilized the Objective Structured Assessment of Technical Skill (OSATS) [25] rubric to assess the skills and proficiency of each participant. Each participant performed a catheterization attempt before the educational session and again after completion of the training. The procedures were evaluated by senior urology residents supervising the simulation session, who were familiar with the OSATS evaluation tool. Each step in the rubric was assigned one point, for a maximum score of 15. Pre- and post-training OSATS scores were summarized using mean±standard deviation and compared using paired statistical testing to assess improvement in technical performance following the training.

The assessed skills included history taking for indications and contraindications, correct choice of balloon inflation solution, catheter type and material, proper patient and operator positioning, adequate sterilization of the genitals and maintenance of sterile technique during insertion, optimal exposure of urethral meatus, application of lube, penile stretching and angling, catheter insertion, balloon inflation, and urine drainage (Table 1).

**Table 1:** OSATS rubric for trainee evaluation concerning urethral catheterization.

Skill performance checkpoints	Points
Indication (history) check	1
Contraindications check	1
Choice of catheter type	1
Choice of catheter size	1
Patient positioning	1
Operator positioning	1
Genital cleansing	1
Balloon integrity check	1
Exposure of urethral meatus	1
Urethra alignment	1
Lubricant application	1
Depth of insertion of the catheter	1
Choice of balloon inflation material	1
Verification of the drained urine volume	1
Documentation	1

OSATS = Objective Structured Assessment of Technical Skills. Each checkpoint was assigned 1 point, for a maximum total score of 15.

### Training development

The training curriculum was developed based on previous works in literature [7,17]. All teaching sessions were given by an experienced urologist with more than five years of teaching experience. The learning objectives of the simulation session were to educate the participants on the indications, contraindications, and the steps of a correct UC, while also enhancing the technical skills and confidence in their performance of UC.

After the pretest, the first theoretical module involved a short, interactive PowerPoint presentation which highlighted key points in UC: anatomical considerations in males and females, indications, contraindications, catheter types and selection criteria, detailed steps of UC in males and females, complications, and the importance of documentation and seeking help for complex cases. There was also emphasis on the importance of the “one gentle attempt only” rule to reduce the likelihood of iatrogenic false passage^10^. Materials were selected from the Campbell-Walsh Urology, 11^th^ edition [23]. In the second theoretical module, catheter insertion was demonstrated by videos showing the steps of UC with verbal descriptions. Videos were specific to the female and male dummies available at the simulation center, and were picked from the provider’s website [26,27]. Lastly, interns reattempted UC on male and female dummies under the supervision of the senior urology residents, who commented on the technique and outlined the flaws. The senior residents again utilized the OSATS rubric to assess improvement of technical skills.

The workshop was conducted in small groups of approximately eight participants to facilitate interaction and individualized instruction. Each group was supervised by a senior urology resident, resulting in an approximate instructor-to-learner ratio of 1:8 during the simulation exercises. The hands-on simulation module lasted approximately 20 minutes, during which participants practiced urethral catheterization on male and female simulation models. Trainees were encouraged to perform the procedure while verbalizing each step. Feedback was provided immediately after each attempt using a structured, formative approach, in which the supervising resident highlighted correct steps, identified technical errors, and provided practical suggestions for improvement. This immediate feedback aimed to reinforce correct technique and address common procedural mistakes.

### Transfer of learning

The educational impact of the simulation was assessed following Kirkpatrick’s four-level model. Levels 1 and 2 (reaction and learning) were evaluated through participant satisfaction, knowledge, confidence, and perceived self-efficacy immediately after the session. Levels 3 and 4 (behavioral change and results) were assessed at six-month follow-up through clinical performance indicators, number of catheterizations performed, procedural success rate, and complication rate, providing evidence of transfer of learning into clinical practice [28].

Six months after the simulation, a remote assessment was conducted to evaluate behavioral change (Level 3) and patient-related outcomes (Level 4). Data on the number, success, and complications of catheterizations were collected from interns’ clinical logbooks and verified by supervising urology residents. This approach allowed pragmatic evaluation of how simulation-based training translated into real clinical performance.

Procedural success was defined as successful insertion of the urinary catheter with appropriate urine drainage and no need for additional attempts by another operator or alternative catheterization techniques. Complications were defined as any adverse event occurring during or following the catheterization procedure, including urethrorrhagia, false passage creation, urinary tract infection, or the need for suprapubic catheterization. Follow-up data were collected from interns’ procedural logbooks during their clinical rotations and were reviewed in collaboration with supervising urology residents and physicians to verify the reported number of procedures, procedural outcomes, and complications.

### Statistical analysis

Interns’ declarative knowledge and PSE scores were reported using descriptive statistics with means and standard deviations or frequencies and percentages. Pre- and post-training scores were compared using paired t-tests. Although perceived self-efficacy and confidence were measured using Likert-type scales, these outcomes were treated as approximately continuous variables and summarized using means and standard deviations, a commonly accepted approach in educational research for within-subject comparisons. Effect sizes with corresponding 95% confidence intervals were also reported to facilitate interpretation of the magnitude of observed changes. Statistical significance was defined as p < 0.05.

Data from surveys were collected using an online form. The analysis was conducted using RStudio 2021.09.0 + 351 “Ghost Orchid” Release [29]. The inclusion of six-month outcome data permitted assessment of behavioral change and patient-level results (Kirkpatrick Levels 3 and 4), expanding the evaluation beyond knowledge and self-efficacy gains.

### Ethical considerations

This study was reviewed and approved by the Ethical Review Board of the Holy Spirit University of Kaslik (USEK), Jounieh, Lebanon (Approval number: EN-CETH-001). All participants were informed about the objectives of the study prior to enrollment. Written informed consent was obtained from all participants before participation. The study procedures were conducted in accordance with the ethical principles outlined in the Declaration of Helsinki.

## Results

### Participant demographics

The study initially recruited 44 interns, however, 4 were not included as they had previously participated in general simulation sessions that included foley catheterization during their undergraduate training.

40 new medical interns participated in the urology simulation session, of which 35% (n = 14) were males and 65% (n = 26) were females. All had completed five years of studies at the same medical school.

### Participant initial knowledge

All participants considered history taking and contraindications recognition essential before UC and were able to identify at least 3 indications, such as urinary drainage during retention, control of incontinence, and bladder irrigation, and contraindications, such as urethral trauma, acute infection, and known bladder tumors. The majority (92%) did not know the difference between catheter types and sizes. Only 38% (n = 15) would position the patient supine for catheterization, and 43% (n = 17) would stand at the correct patient side, which is the side of the operator’s dominant hand. All of them agreed that the procedure should be sterile. Most participants did not know how to correctly expose the urethral meatus (75%, n = 30), the importance of the solution used to inflate the balloon (100%, n = 40), or that lubricant must be applied into the urethra (25%, n = 10). In contrast, most interns (83%, n = 33) knew that the penis should be stretched perpendicular to the body during catheterization, and that the catheter had to be introduced to the hub (65%, n = 26). The majority agreed on the importance of documentation (85%, n = 34) and assessment of the volume drained (90%, n = 36) (Table 2 and further detailed in S1 Table).

**Table 2:** Pre- and post-test interns’ declarative knowledge concerning urethral catheterization.

Questions	Pre-training correct n(%)	post-training correct n(%)	McNemar p-value
What are the indications for UC?	40 (100)	40 (100)	–
What are the contraindications for UC?	40 (100)	40 (100)	–
Do you know the difference between catheter types?	3 (7.5)	34 (85.0)	**<0.001**
Are you able to correctly choose the catheter size?	4 (10.0)	31 (77.5)	**<0.001**
What is the best patient position during UC?	15 (37.5)	40 (100)	**<0.001**
Where is the best position of the operator who is performing the UC?	17 (42.5)	17 (42.5)	–
Should the UC be a sterile procedure?	40 (100)	40 (100)	–
Should the balloon be checked before UC?	1 (2.5)	25 (62.5)	**<0.001**
Do you know how the correctly expose the urethral meatus in males and females?	10 (25.0)	10 (25.0)	–
At which angle should the penis be stretched during UC?	33 (82.5)	33 (82.5)	–
Where is the lubricant best applied before UC?	25 (62.5)	33 (82.5)	**<0.001**
What is the level or depth of UC insertion?	26 (65.0)	35 (87.5)	**0.004**
Is the type of solution used to inflate the balloon important?	0 (0)	37 (92.5)	**<0.001**
Is the volume of urine drained important?	36 (90.0)	36 (90.0)	–
Is procedure documentation important?	34 (85.0)	37 (92.5)	0.453

Values are presented as n (%).

### Variation in knowledge, PSE, and confidence levels

**Fig 1:**
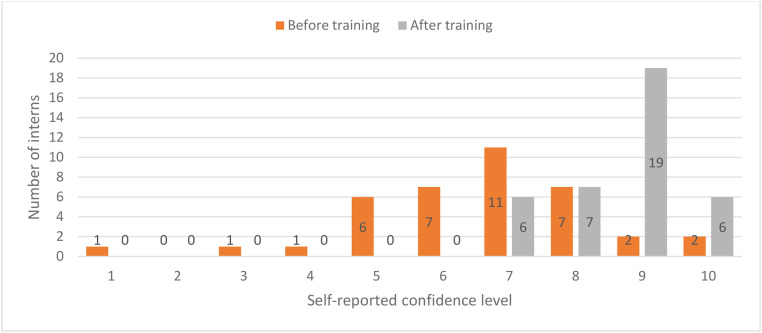
Self-reported confidence levels before and after the boot camp.

The pre- and post-training assessments showed an increase in the mean declarative knowledge score from 7.7 ± 1.24 to 12.2 ± 1.11, and in the mean PSE score, which increased from 3.32 ± 0.94 to 4.32 ± 0.62. Prior to training, the self-reported confidence levels (SRCL) ranged from 1 to 10, with a median of 7 and a mean of 6.6 ± 1.79. After the training session, the levels varied exclusively between 7 and 10. The mean confidence level was 8.66 ± 0.94, with a median of 9. All the improvements showed a statistical significance (p < 0.001). The magnitude of the improvements is reflected by the mean differences and their 95% confidence intervals, indicating substantial gains in knowledge, perceived self-efficacy, and confidence following the training session (Table 3, Fig 1). Nevertheless, several specific knowledge items showed no measurable improvement following training, including operator positioning, correct exposure of the urethral meatus, penile traction angle, urine volume assessment, and procedure documentation, highlighting areas that may require greater emphasis in future training sessions

**Table 3:** Changes in the scores of knowledge, PSE, SRCL, and OSATS for TS and NTS before and after training.

	Before training	After training	Mean of difference [95%CI]	P-value
**Knowledge scores**	**7.7 ± 1.24**	**12.2 ± 1.11**	**4.5 [4.07-4.92]**	**<0.05**
**PSE scores**	**3.32 ± 0.94**	**4.32 ± 0.62**	**1 [0.65-1.36]**	**<0.05**
**SRCL scores**	**6.6 ± 1.79**	**8.66 ± 0.94**	**2.05 [1.39-2.72]**	**<0.05**
**TS scores**	**8.9 ± 2.1**	**12.8 ± 2.3**	**3.9 [3.1-4.8]**	**<0.01**
**NTS scores**	**6.8 ± 2.2**	**7.2 ± 3.1**	**0.4 [−0.6 to 1.4]**	**0.4**

PSE = : perceived self-efficacy; SRCL = self-reported confidence levels; OSATS = Objective Structured Assessment of Technical Skills; TS = technical skills; NTS = non-technical skills; Values are presented as mean±standard deviation.

P-values were calculated using paired statistical testing comparing scores before and after training.

### Participant satisfaction

Prior to the training, participants were asked to self-report their readiness to perform UC in the hospital, resulting in 34 (85%) feeling unprepared to perform the procedure and only 15% (n = 6) feeling adequately prepared. The most preferred teaching modalities among the new interns were live simulation (n = 22, 55%) followed by supervised bedside teaching (n = 18, 45%) (Table 4). At the end of the training, Preparedness was reassessed immediately after the training through the post-training satisfaction survey, which showed that 68% (n = 26) of the trainees were adequately prepared to perform the procedure, while 92.5% (n = 37) were very satisfied with the training. All (n = 40) rated the quality of the training as good and 97.5% (n = 39) reported that the training met their needs and that the skills acquired were useful in their daily work. Almost all trainees (97.5%, n = 39) stated that they would recommend this training to others (Table 5).

**Table 4:** Pre-test participant preparedness and preferred method of learning.

Feeling adequately prepared for UC*	Yes	6 (15%)
No	34 (85%)
Preferred method of training	Live simulation demonstration	22 (55%)
	Supervised bedside catheterization	18 (45%)
	Video	0 (0%)
	Conference	0 (0%)

UC = Urethral catheterization.

Percentages are calculated based on the total number of participants (N = 40).

**Table 5:** Participant satisfaction towards the boot camp.

Domain	Answer	n	%
Satisfaction	Very well	37	92.5%
	Ok	3	7.5%
Quality of training	Very well	40	100%
	Ok	0	0%
Meeting the needs of trainees	Very well	39	97.5%
	Ok	1	2.5%
Usefulness of the skills acquired in daily practice	Yes	39	97.5%
	No	1	2.5%
Recommendation of the exercise to other trainees	Yes	39	97.5%
	No	1	2.5%

Percentages are calculated based on the total number of participants (N = 40).

Responses were collected through a post-training satisfaction questionnaire.

### Transfer of learning outcomes

Over 6 months, interns collectively performed 274 urinary catheterizations, reflecting sustained application of learned skills in clinical settings (Level 3). The overall procedural success rate was 93% (n = 255), with a low failure rate of 6.9% (n = 19) and complication rate of 4% (n = 11) (Level 4), indicating that simulation training positively influenced both behavior and patient outcomes. The median number of procedures performed per intern was 5 [IQR: 4–10], demonstrating variability in individual procedural exposure.

Of the 19 failed attempts, the most common causes of failure were urethral meatal stenosis in 32% (n = 6) and difficulty in identifying the urethral meatus in women in 32% (n = 6). Other reported causes were inadequate exposure of the genitals due to patient disability (e.g., hip fracture) in 26% (n = 5) and phimosis in male patients in 10% (n = 2). Overall, 4% (n = 11) were complicated.

The most common complication was urethrorrhagia with spontaneous resolution in 55% (n = 6) of cases. This was followed by the occurrence of a false passage in 18% (n = 2), a urinary tract infection in 18% (n = 2) and the need for suprapubic catheterization in 9% (n = 1) (Table 6).

**Table 6:** Rate and causes of urinary catheterization failure and complications.

Description	n	%
Total UC performed	**274**	
Successful attempts	**255**	**93.1%**
Failed attempts	**19**	**6.9%**
Reasons for failed attempts (n = 19)		
Difficulty identifying urethral meatus (Female)	6	31.6%
Phimosis (Male)	2	10.5
Inadequate exposure of the genitals	5	26.3%
Stenosis of the urethral meatus	6	31.5%
Complications	**11**	**4.0%**
Urethrorrhagia with spontaneous resolution	6	54.5%
False passage creation	2	18.2%
Urinary tract infection	2	18.2%
Need for suprapubic catheterization	1	9.1%

UC = urethral catheterization.

% for total failure and complication rates are based on total catheterisation attempts (n = 274).

% for reasons for failed attempts are based on total failed attempts (n = 19).

% for complications are based on total complications (n=11).

## Discussion

The primary purpose of the simulation session was to bridge the gap between the knowledge gained in medical school and the technical skills needed in clinical practice. Based on the previous literature showing that new interns caused higher UC-related morbidity [1], this training was organized at the beginning of the internship with the aim of teaching new interns the essential steps and basic clinical skills for UC and allowing them to practice on dummies before catheterizing real patients, which may help support the development of confidence and competence in performing UC [17].

The self-assessment questionnaire administered before the training helped the trainees identify their weaknesses, strengths, knowledge, and PSE regarding UC. Only 15% of the new interns felt adequately prepared to perform UC. The rate was lower than what was reported in the literature by Manalo et al. (55.6%) [17], Thomas et al. (36% and 52%) [7], and Bigot et al. (26% and 38.3%) [19], which raises the need to focus on procedural skill teaching in medical school and emphasizes the importance of the simulation session organized. All our interns (100%) were aware of the importance of history-taking before proceeding to catheterization, in contrast to 23.6% [17] and 26.7% [30] reported in previous studies by Manalo et al. and Browne et al, respectively. This step is of great importance because it allows the recognition of risk factors of complications [17]. More than half of the participants (63%) knew adequately that the lubricant should be applied inside the urethra, despite the tendency to apply it to the catheter, as reported in 97.8% of cases in the Manalo et al. study [17]. Most of our interns were aware of the importance of documentation (85%) and assessment of the volume drained (90%), in contrast with the low rates of documentation (only in 29% of patients with an indwelling catheter) reported by Cornia et al. [31]

The training focused on the weaknesses identified by the initial survey regarding catheter types and sizes, patient positioning during catheterization, operator positioning at the bedside, correct exposure of the urethral meatus, and the importance of the solution used to inflate the balloon. The short learning modules of 20 minutes each was efficient to increase knowledge retention while maintaining attention and focus. The division of interns into small groups of 8 individuals helped optimize the learning experience without excessive crowding and time restrictions. Also, the incorporation of different methods in the training, such as a didactic lecture, video-based demonstration, and hands-on simulation, maximized the teaching experience, as demonstrated by previous works [32,33]. Despite being underreported in various urology curricula, simulation-based learning has demonstrated its efficacy in urology training [32,34]. Participation in the simulation session was associated with an increase in declarative knowledge and PSE scores among the new interns concerning UC. Similarly, Waters et al. demonstrated the beneficial effects of interactive teaching of UC on the confidence level and examination scores [32]. Browne et al. also reported a beneficial effect of the structured practical training on the SRCL of new interns on a different categorical scale [30]. Despite the overall improvement in knowledge scores, several individual knowledge items demonstrated little or no measurable improvement following training, including operator positioning, correct exposure of the urethral meatus, penile traction angle, and the importance of assessing urine volume drained after catheterization. One possible explanation is that baseline performance for some of these items was already relatively high, limiting the potential for measurable gains. For other domains, particularly operator positioning and urethral meatus exposure, the simulation training may not have devoted sufficient emphasis to these concepts compared with other aspects of catheterization such as catheter selection, patient positioning, and balloon inflation. These findings are important because they help identify specific procedural elements that may require enhanced instruction, additional simulation practice, or focused feedback in future iterations of the curriculum.

The inclusion of a six-month follow-up assessment extends this study beyond Kirkpatrick Level 2, demonstrating measurable behavioral change and patient-related outcomes. The high procedural success and low complication rates observed during follow-up provide descriptive insight into interns’ early clinical experience with catheterization. However, these observational data should be interpreted cautiously, as ongoing clinical exposure, supervision, and repeated procedural practice during the internship period may also have influenced these outcomes.

The originality of this study remains in its timing, clear methodology, and objective outcome assessment. The academic timing of the simulation session just before the beginning of the internship both boosted learning capabilities for the participants while also reducing bias in participant experience. At the time of the simulation session, interns would have completed medical school and acquired sufficient medical background to understand the anatomical and physiological basis needed for UC, however, as shown in the results, few of them have ever performed UC on patients. The timing proposed in this study differed from the previous work done by Waters et al., where teaching targeted second-year medical students [32]. To facilitate the assimilation of the UC, there was focus on the essential steps using the standard two-way catheters, without much complicated discussion of the other specific aspects of UC, such as three-ways catheterization, bladder washout, and continuous bladder irrigation, like Browne et al. [30] because those topics were considered more specific to urology residents. The impact of the training session was assessed using an objective declarative knowledge score that was calculated before and after the training, without exclusive reliance on the self-reported outcome such as the perceived-self efficacy and SRCL in this study as in previous works in the literature [30].

This study had several limitations. First, The study did not include a parallel control group, as all new interns at the university were required to attend the simulation session as part of their induction curriculum. Although the pre-test served as a within-subject baseline, future studies should adopt a controlled or randomized design comparing simulation-based teaching with traditional instruction to better delineate causal effects. Second, the effect of the training on UC success and complications in clinical practice on real patients was not observed by the evaluators in the simulation session. Instead, at this stage, there was more reliance on self-reported parameters and knowledge. Third, the small population size, while representative of the population of medical graduates working as new interns, may be a limiting factor for larger programs that incorporate hundreds of new interns every year, emphasizing the importance of this study to be redone in different settings such as university hospitals with larger intern bodies. Also, given the single-group pre–post design of this study, the observed improvements in knowledge, confidence, and clinical performance cannot be attributed exclusively to the simulation session. During the six-month follow-up period, interns were also exposed to routine clinical practice, supervision from senior residents and attending physicians, and repeated procedural experience, which may have contributed to the observed outcomes. Therefore, the results should be interpreted as an association between participation in the training program and improved outcomes rather than a definitive causal relationship. Finally, the study method used simulation with peer feedback, which is considered superior to other conventional learning styles such as lectures, videos, and demonstrations. However, metric-dependent proficiency-based progression training is recently considered the most efficient and performant way to optimize the learning experience [32]. As such, this study may further benefit as a steppingstone to assess the use of metric-dependent proficiency-based progression in different surgical specialties and especially in urology.

## Conclusion

Junior doctors have insufficient knowledge regarding UC. Participation in the urology simulation session was associated with improvements in declarative knowledge scores, SRCL, and perceived self-efficacy regarding UC among new interns, showing promise in improving technical skills and confidence for newly graduated interns being introduced into medical practice. Future studies using controlled or randomized designs would be valuable to better isolate the specific impact of simulation-based training on clinical outcomes.

### Lessons for practice

- Simulation-based training for urethral catheterization may help bridge the gap between theoretical knowledge and clinical performance among new medical interns.
- Early, structured procedural training during internship orientation may contribute to improvements in interns’ confidence, technical proficiency, and patient safety outcomes.
- Incorporating simulation sessions into intern induction programs supports sustainable improvements in procedural success rates and reductions in catheter-related complications.

## Supporting information

S1 Table Detailed pre-and post-training interns’ declarative knowledge concerning urethral catheterization. (DOCX)

## Abbreviations

UC: urinary catheterization
PSE: perceived self-efficacy
CHUNDS: Notre Dame des Secours University Hospital Center
USEK: Holy Spirit University of Kaslik
OSATS: Objective Structured Assessment of Technical Skill
SD: standard deviation
