# Supplementary material for: Enhancing medical intern competence in urethral catheterization: Impact of simulation-based training on knowledge, self-efficacy, and clinical outcomes
Source: PLoS One. 2026 Jul 17;21(7):e0353677. doi: 10.1371/journal.pone.0353677 (PMC13379000; doi:10.1371/journal.pone.0353677)
Supplement: S1 Table — (DOCX) [file pone.0353677.s001.docx]

Enhancing medical intern competence in urethral catheterization: impact of simulation-based training on knowledge, self-efficacy, and clinical outcomes.

| Supp. Table 1. Detailed pre-and post-training interns' declarative knowledge concerning urethral catheterization | | | |
| --- | --- | --- | --- |
| Questions | **Answers** | **Pre-training answers n ()** | **Post-training answers n ()** |
| What are the indications for UC? | Given 3 correct answers | 40 (100) | 40 (100) |
|  | Less than 3 correct answers | 0 (0) | 0 (0) |
| What are the contraindications for UC? | Given 3 correct answers | 40 (100) | 40 (100) |
|  | Less than 3 correct answers | 0 (0) | 0 (0) |
| Do you know the difference between catheter types? | Yes | 3 (8) | 34 (85) |
|  | No | 37 (92) | 6 (15) |
| Are you able to correctly choose the catheter size? | Yes | 3 (8) | 31 (77.5) |
|  | No | 37 (92) | 9 (22.5 |
| What is the best patient position during UC? | Supine | 15 (38) | 40 (100) |
|  | Semi-seated | 25 (62) | 0 (0) |
| Where is the best position of the operator who is performing the UC? | The patient side which is the same as the operator's dominant hand | 17 (43) | 17 (43) |
|  | The patient side which is opposite to the operator's dominant hand | 23 (57) | 23 (57) |
| Should the UC be a sterile procedure? | Yes | 40 (100) | 40 (100) |
|  | No | 0 (0) | 0 (0) |
| Should the balloon be checked before UC? | Yes | 1 (3) | 25 (62.5) |
|  | No | 39 (97) | 15 (37.5) |
| Do you know how the correctly expose the urethral meatus in males and females? | Yes | 10 (25) | 10 (25) |
|  | No | 30 (75) | 30 (75) |
| At which angle should the penis be stretched during UC? | Perpendicular to the body | 33 (83) | 33 (0.83) |
|  | Parallel to the body | 7 (17) | 7 (0.17) |
|  | No particular angle | 0 (0) | 0 (0) |
| Where is the lubricant best applied before UC? | Catheter | 25 (63) | 3 (7.5) |
|  | Urethra | 10 (25) | 33 (82.5) |
|  | Urethral meatus | 5 (12) | 4 (10) |
| What is the level or depth of UC insertion? | To the hub (where the connection for a drainage tube and the inflation port meet) | 26 (65) | 35 (87.5) |
|  | Past the mid-point of the shaft of the catheter | 5 (12) | 0 (0) |
|  | No particular level: the balloon is inflated as soon as the urine passes from the catheter | 9 (23) | 5 (12.5) |
| Is the type of solution used to inflate the balloon important? | Yes | 0 (0) | 37 (92.5) |
|  | No | 40 (100) | 3 (7.5) |
| Is the volume of urine drained important? | Yes | 36 (90) | 36 (90) |
|  | No | 4 (100) | 4 (100) |
| Is procedure documentation important? | Yes | 34 (85) | 37 (92.5) |
|  | No | 6 (15) | 3 (2.5) |
