# Supplementary material for: Enhancing medical intern competence in urethral catheterization: Impact of simulation-based training on knowledge, self-efficacy, and clinical outcomes
Source: PLoS One. 2026 Jul 17;21(7):e0353677. doi: 10.1371/journal.pone.0353677 (PMC13379000; doi:10.1371/journal.pone.0353677)
Supplement: Attachment — Submitted filename: Review response Foley.pdf [file pone.0353677.s005.pdf]

Dear Editors and Reviewers of PLOS ONE,

We would like to sincerely thank the Editor and the Reviewers for their careful evaluation of our manuscript and for the constructive comments provided. We have revised the manuscript accordingly and believe that these revisions have significantly improved the clarity, transparency, and scientific rigor of the work.

Below we provide a detailed, point-by-point response to each comment. All corresponding changes have been incorporated into the revised manuscript.

Editor comments

1.Please ensure that your manuscript meets PLOS ONE's style requirements, including those for file naming. The PLOS ONE style templates can be found at

https://journals.plos.org/plosone/s/file?id=wjVg/PLOSOne_formatting_sample_main_body.pdf and https://journals.plos.org/plosone/s/file?id=ba62/PLOSOne_formatting_sample_title_authors_affiliations.pdf

Response: The manuscript formatting has been revised to ensure full compliance with the formatting and style requirements of PLOS ONE, including file structure and labeling.

2. We note that you have indicated that there are restrictions to data sharing for this study. PLOS only allows data to be available upon request if there are legal or ethical restrictions on sharing data publicly. For more information on unacceptable data access restrictions, please see http://journals.plos.org/plosone/s/data-availability#loc-unacceptable-data-access-restrictions.

Before we proceed with your manuscript, please address the following prompts:

a) If there are ethical or legal restrictions on sharing a de-identified data set, please explain them in detail (e.g., data contain potentially identifying or sensitive patient information, data are owned by a third-party organization, etc.) and who has imposed them (e.g., a Research Ethics Committee or Institutional Review Board, etc.). Please also provide contact information for a data access committee, ethics committee, or other institutional body to which data requests may be sent.

b) If there are no restrictions, please upload the minimal anonymized data set necessary to replicate your study findings to a stable, public repository and provide us with the relevant URLs, DOIs, or accession numbers. For a list of recommended repositories, please see

https://journals.plos.org/plosone/s/recommended-repositories. You also have the option of uploading the data as Supporting Information files, but we would recommend depositing data directly to a data repository if possible.

We will update your Data Availability statement on your behalf to reflect the information you provide.

Response: To comply with the data sharing policies of PLOS ONE, the de-identified dataset supporting the findings of this study has been deposited in the public repository BioStudies, hosted by the European Bioinformatics Institute.

The dataset is available under accession number S-BSST2802 and can be accessed at:

https://www.ebi.ac.uk/biostudies/studies/S-BSST2802

The Data Availability Statement in the manuscript has been updated accordingly.

3. Please include your full ethics statement in the 'Methods' section of your manuscript file. In your statement, please include the full name of the IRB or ethics committee who approved or waived your study, as well as whether or not you obtained informed written or verbal consent. If consent was waived for your study, please include this information in your statement as well.

Response: Thank you for this important suggestion. We have now expanded the ethics statement in the Methods section to include the full name of the approving ethics committee. The manuscript now specifies that the study was approved by the Ethical Review Board of the Holy Spirit University of Kaslik (USEK), Jounieh, Lebanon, and that written informed consent was obtained from all participants prior to enrollment. The study's compliance with the principles of the Declaration of Helsinki has also been clarified.

4. Please upload a copy of Figures 1-3, to which you refer in your text on page 11. If the figure is no longer to be included as part of the submission please remove all reference to it within the text.

Response: These figures were not intended to be included in the final submission; therefore, all references to Figures 1–3 have been removed from the manuscript.

5. Please include a copy of Table 1-6 which you refer to in your text on page 7,10,11 and 12.

Response: Tables 1–7 have now been included in the revised manuscript and appropriately labeled. We also corrected a referencing error in the text where Table 6 was cited twice; the second citation has now been corrected to Table 7.

Reviewers' comments:

Reviewer #1: This manuscript addresses a relevant and practical training need (urethral catheterization) using a simulation-based workshop for new interns. The pre-post improvements in knowledge and self-reported outcomes are promising. To strengthen clarity, transparency, and reproducibility, several aspects of reporting and interpretation would benefit from further clarification. I therefore recommend major revision.

1. **Interpretation in light of the study design**

Given the single-group pre–post design, please consider softening causal language in the Discussion and Conclusions. In particular, it would be helpful to frame findings in descriptive terms and to acknowledge that other factors during the follow-up period (e.g., ongoing clinical exposure or supervision) may also have contributed to the observed changes.

Response: We appreciate this important comment. The Discussion and Conclusion sections have been revised to adopt more descriptive language when interpreting the findings. We now explicitly acknowledge that improvements observed during follow-up may also reflect ongoing clinical exposure, supervision by senior physicians, and routine clinical practice during the internship period. These revisions clarify that the observed improvements cannot be attributed solely to the simulation intervention.

2. Reporting of skills outcomes (OSATS)

OSATS is described as an important skills assessment. Please clarify whether pre- and post-training OSATS results are available and, if so, present summary statistics and the corresponding statistical analysis. Additional brief information on who performed the ratings and how assessments were conducted would help readers interpret these results.

Response: Pre- and post-training Objective Structured Assessment of Technical Skills (OSATS) evaluations were indeed conducted. The corresponding summary statistics and statistical comparisons are now reported in Table 3.

To further improve clarity, the Methods section now includes additional details regarding the evaluation process. Catheterization procedures were assessed by senior urology residents supervising the simulation sessions, using the standardized OSATS rubric. Participants were evaluated before the educational intervention and again following completion of the training, allowing direct comparison of technical performance.

3. Description of measurement tools

For the knowledge assessment, please provide additional detail on the test items and scoring (either in the main text or as Supporting Information), as well as how content relevance was established. For self-efficacy and confidence measures, please clarify whether these items were adapted from existing instruments or locally developed, and briefly justify their use.

Response: We have expanded the Methods section to provide further detail regarding the knowledge assessment and scoring system. The questionnaire consisted of multiple-choice and open-ended questions addressing key aspects of urethral catheterization, including indications, contraindications, catheter types and sizes, patient positioning, sterile technique, procedural steps, and complication prevention. Each correct response was assigned one point, generating a cumulative knowledge score.

The questionnaire content was developed based on established urological educational resources, including Campbell-Walsh Urology, to ensure content relevance.

The self-efficacy and confidence measures were locally developed for this educational evaluation and utilized Likert-type scales commonly used in simulation-based education research to capture participants' perceived preparedness and confidence before and after training.

4. Statistical analysis

Please clarify the rationale for using paired t-tests for Likert-scale outcomes, or consider alternative non-parametric analyses if more appropriate. Where feasible, reporting effect sizes with confidence intervals alongside p-values would further aid interpretation.

Response: In this study, Likert-scale outcomes (perceived self-efficacy and confidence) were treated as approximately continuous variables and summarized using means and standard deviations, which is a commonly accepted approach in educational research when evaluating within-participant pre-post changes. Accordingly, paired t-tests were used for statistical comparison.

To improve interpretability, the Methods section now clarifies this rationale, and effect size estimates with 95% confidence intervals have been added to the Results section alongside the reported p-values.

5. Six-month follow-up outcomes

Please define the criteria used for “success” and “complications” and briefly describe how follow-up data were collected and verified. Given the observational nature of these data, a more descriptive interpretation in the discussion would be appropriate.

Response: We have clarified these definitions in the Methods section.

Procedural success was defined as successful insertion of the urinary catheter with adequate urine drainage without the need for assistance or alternative catheterization techniques.

Complications included adverse events occurring during or immediately after catheterization, such as urethrorrhagia, false passage creation, urinary tract infection, or the need for suprapubic catheterization.

We also clarified that six-month follow-up data were obtained from intern procedural logbooks and confirmed with supervising urology residents. The Discussion section has been revised to present these findings in a descriptive manner, acknowledging their observational nature.

6. Intervention description

To support reproducibility, please consider adding a few practical details on the workshop implementation (e.g., approximate instructor-to-learner ratio, hands-on practice time, and the general approach to feedback).

Response: The description of the simulation workshop has been expanded in the Methods section to include additional practical details, including the instructor-to-learner ratio, duration of hands-on practice, and the structured feedback approach used during the training sessions. These additions aim to improve the reproducibility of the educational intervention.

7. Data availability

Please review the data availability statement to ensure it aligns with PLOS ONE policies, for example, by indicating whether de-identified data or materials can be shared via a repository or other access mechanism.

Response: The Data Availability Statement has been updated to comply with PLOS ONE policies. The de-identified dataset supporting this study has been deposited in the public repository BioStudies under accession number S-BSST2802.

8. Other

- Please ensure consistent use of key terms (e.g., self-efficacy/confidence).

- Check that all tables and figures referenced in the text are included and clearly labeled.

- Minor language editing would improve clarity in a few places.

Response: We have carefully reviewed the manuscript to ensure consistent terminology, particularly regarding perceived self-efficacy and confidence. All tables and figures referenced in the manuscript have been verified and correctly labeled. The manuscript has also undergone minor language editing to improve clarity and readability.

We thank the Editor and Reviewers again for their valuable comments and insightful suggestions. We believe that the revisions made in response to these comments have strengthened the manuscript and improved its clarity and methodological transparency.

We appreciate your consideration of our revised manuscript.

Sincerely,

Maher Abdessater

On behalf of all co-authors
