# Supplementary material for: Enhancing medical intern competence in urethral catheterization: Impact of simulation-based training on knowledge, self-efficacy, and clinical outcomes
Source: PLoS One. 2026 Jul 17;21(7):e0353677. doi: 10.1371/journal.pone.0353677 (PMC13379000; doi:10.1371/journal.pone.0353677)
Supplement: Attachment — Submitted filename: Response to Reviewer 1.pdf [file pone.0353677.s008.pdf]

Response to Reviewer 1

We sincerely thank the reviewer for the thoughtful and constructive comments. We greatly appreciate the time and effort dedicated to reviewing our manuscript. The suggestions have significantly improved the clarity, organization, and scientific rigor of the paper. All comments were carefully considered and addressed as detailed below.

Comment 1

The term “intern” is used throughout the manuscript; however, this may be ambiguous, as it can refer to different healthcare professions (e.g., nursing, pharmacy, or medicine). For clarity and precision, I recommend specifying “medical intern” in the title, and in the content for the first mention.

Response: The terminology has been revised throughout the manuscript to specify “medical intern” where appropriate, including in the title and at first mention within the text, in order to improve clarity and avoid ambiguity regarding the study population.

Comment 2

Some statements need revision, as detailed in the document. For example, the phrase “... questionnaire conducted ....” should be revised to “... questionnaire administered ....”

Response: The manuscript has undergone thorough linguistic revision, and the suggested wording changes, including replacing “questionnaire conducted” with “questionnaire administered,” have been implemented throughout the manuscript to improve readability and academic style.

Comment 3

It is indicated that training was provide to the new interns, however, it is not specified at what point of internship. This is only mentioned in the discussion, please consider clarifying when (specific time) the medical interns were recruited and trained in the methods section.

Response: The Methods section has been revised to clarify the timing of recruitment and training. Specifically, we now state that the simulation workshop was conducted at the beginning of the interns’ academic year, prior to substantial clinical exposure, as part of their early internship orientation and training.

Comment 4

The ethics statement (in the Methods introduction) indicates approval by the "University Institutional Review Board," however the specific university/institution is not identified. For clarity and transparency, please specify the name of the university (and include the approval reference number if available). Also, the ethical statements are repeated in both the Methods introduction and Ethical considerations sections. These can be consolidated under Ethics consideration section:

Response: The ethical approval statement has been clarified and consolidated within the dedicated "Ethical considerations" section. The manuscript now specifies that the study was approved by the Ethical Review Board of the Holy Spirit University of Kaslik (USEK), Jounieh, Lebanon (Approval number: EN-CETH-001). Redundant ethical statements were removed from the Methods introduction to improve manuscript organization and avoid repetition.

Comment 5

Two variables "Feeling adequately prepared for UC" and "Preferred method of training" are included in demographic tables, however, they do not appropriate fit as demographic characteristics. You may consider reporting them with satisfaction finding.

Response: These variables were removed from the demographic characteristics table and reorganized into a separate section addressing participant preparedness and training preferences, alongside satisfaction-related findings, to improve the conceptual consistency of the Results section.

Comment 6

The initial declarative knowledge is provided in the narrative and then indicates that these are also presented in a table 3. However, the corresponding table 3 does not display the pre-test scores as stated. Instead, table 3 displays the changes in the OSATS scores for TS and NTS before and after training. Please address this across the results to ensure consistency between texts and corresponding tables.

Response: The Results section and tables were revised for consistency and clarity. The updated Table 3 now presents both pre- and post-training declarative knowledge responses side by side, together with McNemar test p-values, allowing direct comparison of knowledge acquisition following the intervention.

Comment 7

While the summary of overall and change in knowledge scores (Table 5.) is appropriately presented with statistical comparison, the item-level table reports only pre-training responses (Table 4). Given that post-training data are available, it would improve clarity and interpretability to present both pre- and post-training responses side by side for each item. This would allow readers to better understand which specific knowledge areas improved following the intervention.

Response: In response, the item-level knowledge table was completely revised. The updated table now presents both pre-training and post-training correct responses for each knowledge item in a side-by-side format, together with corresponding McNemar p-values. This revision substantially improves interpretability and allows readers to identify the specific knowledge domains that improved after the simulation-based intervention.

Comment 8

Also, the item-level presentation of responses is informative; however, reporting all response options for each question may reduce clarity. Consider simplifying the table to present the proportion of correct responses for each item (pre- and post-training), which would more directly reflect knowledge acquisition. Detailed response distributions could be included as supplementary material if needed.

Response: We agree with the reviewer's suggestion. The item-level knowledge table was simplified to report only the proportion of correct responses before and after training for each question, rather than displaying all response options. This improved readability and allowed the table to more directly reflect declarative knowledge acquisition following the intervention. Detailed response distributions were removed from the main manuscript.

Comment 9

In the section "transfer of learning outcomes", the results are reported thought these are difficult to interpret. In particular, it is unclear how the proportions in the table were derived and the denominator used. The total number of urethral catheterisations performed collectively is reported as 274, however, it would be helpful to include the distribution of procedures per medical intern (e.g., range and/or median with IQR) to better understand variability in individual procedural exposure within the six months post training.

Response: The “Transfer of learning outcomes” section and accompanying tables were revised extensively to improve clarity and interpretability. Specifically, explanatory footnotes were added to clarify the denominators used for each percentage calculation:

- procedure outcome percentages were calculated based on the total number of catheterization attempts,
- causes of failed attempts were calculated relative to total failed attempts,
- complication percentages were calculated relative to total complications.

This clarification has now been explicitly stated in the revised table footnotes and Results section.

Comment 10

It would be helpful to include the distribution of procedures per medical intern (e.g., range and/or median with IQR) to better understand variability in individual procedural exposure.

Response: In response, additional descriptive data regarding procedural exposure were added to the Results section. We now report that the median number of urethral catheterizations performed per intern during the six-month follow-up period was 5 [IQR: 4–10], demonstrating variability in individual clinical exposure following the training intervention.

We again sincerely thank the reviewer for the insightful comments and recommendations, which significantly improved the quality, clarity, and scientific presentation of our manuscript.

Sincerely,

Ramy Touma Sawaya, MD

(on behalf of all coauthors
