# Supplementary material for: Enhancing medical intern competence in urethral catheterization: Impact of simulation-based training on knowledge, self-efficacy, and clinical outcomes
Source: PLoS One. 2026 Jul 17;21(7):e0353677. doi: 10.1371/journal.pone.0353677 (PMC13379000; doi:10.1371/journal.pone.0353677)
Supplement: Attachment — Submitted filename: PONE-D-25-64830_R2_reviewer_2026.05.24.pdf [file pone.0353677.s007.pdf]

PLOS One

Enhancing Medical Intern Competence in Urethral Catheterization: Impact of Simulation-Based Training on Knowledge, Self-Efficacy, and Clinical Outcomes.

--Manuscript Draft--

**Manuscript Number:**	PONE-D-25-64830R2
**Article Type:**	Research Article
**Full Title:**	Enhancing Medical Intern Competence in Urethral Catheterization: Impact of Simulation-Based Training on Knowledge, Self-Efficacy, and Clinical Outcomes.
**Short Title:**	Evaluating urinary catheterization simulation training for new interns
**Corresponding Author:**	Ramy Touma Sawaya, M.D.<br>AUBMC: American University of Beirut Medical Center<br>Beirut, Beirut LEBANON
**Keywords:**	urinary catheterization; intern; education; Simulation
**Abstract:**	Introduction: Urethral catheterization is a common procedure, yet most related complications occur when performed by new interns. Studies have shown inadequate training and knowledge among junior doctors. This study evaluated the declarative knowledge of new interns regarding urethral catheterization and assessed the effectiveness of a simulation-based urology session in improving their knowledge, confidence, and perceived self-efficacy.<br>Methods: A one-day urology simulation workshop was conducted for 40 new interns at a university hospital. Participants completed a pre-session online questionnaire assessing demographics, urinary catheterization knowledge, and self-reported confidence and self-efficacy. The workshop included three modules: an interactive presentation, instructional videos, and hands-on simulation on male and female models. Post-session assessments measured changes in knowledge, confidence, and self-efficacy scores using standardized evaluation tools.<br>Results: Forty new medical interns participated. Only 15% felt adequately prepared before training. Knowledge gaps were identified regarding catheter types and sizes (92.5%), optimal patient positioning (62%), proper meatus exposure, and balloon inflation technique (100%). Following the workshop, mean knowledge scores increased from 7.7±1.24 to 12.2±1.11, self-reported confidence from 6.60±1.79 to 8.66±0.94, and perceived self-efficacy from 3.32±0.94 to 4.32±0.62 (all p<0.001). At six-month follow-up, participants collectively performed 274 urethral catheterizations with a 93% success rate and a low complication rate of 4% (n=11), reflecting effective transfer of learning from the simulation to clinical practice.<br>Discussion: New medical interns demonstrated insufficient baseline knowledge of urethral catheterization. The structured urology simulation session significantly improved their declarative knowledge, confidence, and perceived self-efficacy, underscoring the value of simulation-based training in early postgraduate medical education.
**Order of Authors:**	Maher Abdessater
	Anthony Kanbar
	Joey El Khoury
	Rami Halabi
	Ramy Touma Sawaya, M.D.
	Raghid El Khoury
**Response to Reviewers:**	Response to Reviewer 1<br>We sincerely thank the reviewer for the thoughtful and constructive comments. We greatly appreciate the time and effort dedicated to reviewing our manuscript. The suggestions have significantly improved the clarity, organization, and scientific rigor of the paper. All comments were carefully considered and addressed as detailed below.<br>Comment 1<br>The term “intern” is used throughout the manuscript; however, this may be ambiguous,

as it can refer to different healthcare professions (e.g., nursing, pharmacy, or medicine). For clarity and precision, I recommend specifying “medical intern” in the title, and in the content for the first mention.
Response: The terminology has been revised throughout the manuscript to specify “medical intern” where appropriate, including in the title and at first mention within the text, in order to improve clarity and avoid ambiguity regarding the study population.
Comment 2
Some statements need revision, as detailed in the document. For example, the phrase “… questionnaire conducted ….” should be revised to “… questionnaire administered ….”
Response: The manuscript has undergone thorough linguistic revision, and the suggested wording changes, including replacing “questionnaire conducted” with “questionnaire administered,” have been implemented throughout the manuscript to improve readability and academic style.
Comment 3
It is indicated that training was provide to the new interns, however, it is not specified at what point of internship. This is only mentioned in the discussion, please consider clarifying when (specific time) the medical interns were recruited and trained in the methods section.
Response: The Methods section has been revised to clarify the timing of recruitment and training. Specifically, we now state that the simulation workshop was conducted at the beginning of the interns’ academic year, prior to substantial clinical exposure, as part of their early internship orientation and training.
Comment 4
The ethics statement (in the Methods introduction) indicates approval by the “University Institutional Review Board,” however the specific university/institution is not identified. For clarity and transparency, please specify the name of the university (and include the approval reference number if available). Also, the ethical statements are repeated in both the Methods introduction and Ethical considerations sections. These can be consolidated under Ethics consideration section:
Response: The ethical approval statement has been clarified and consolidated within the dedicated “Ethical considerations” section. The manuscript now specifies that the study was approved by the Ethical Review Board of the Holy Spirit University of Kaslik (USEK), Jounieh, Lebanon (Approval number: EN-CETH-001). Redundant ethical statements were removed from the Methods introduction to improve manuscript organization and avoid repetition.
Comment 5
Two variables “Feeling adequately prepared for UC” and “Preferred method of training” are included in demographic tables, however, they do not appropriate fit as demographic characteristics. You may consider reporting them with satisfaction finding.
Response: These variables were removed from the demographic characteristics table and reorganized into a separate section addressing participant preparedness and training preferences, alongside satisfaction-related findings, to improve the conceptual consistency of the Results section.
Comment 6
The initial declarative knowledge is provided in the narrative and then indicates that these are also presented in a table 3. However, the corresponding table 3 does not display the pre-test scores as stated. Instead, table 3 displays the changes in the OSATS scores for TS and NTS before and after training. Please address this across the results to ensure consistency between texts and corresponding tables.
Response: The Results section and tables were revised for consistency and clarity. The updated Table 3 now presents both pre- and post-training declarative knowledge responses side by side, together with McNemar test p-values, allowing direct comparison of knowledge acquisition following the intervention.
Comment 7
While the summary of overall and change in knowledge scores (Table 5.) is appropriately presented with statistical comparison, the item-level table reports only pre-training responses (Table 4). Given that post-training data are available, it would improve clarity and interpretability to present both pre- and post-training responses side by side for each item. This would allow readers to better understand which specific knowledge areas improved following the intervention.
Response: In response, the item-level knowledge table was completely revised. The updated table now presents both pre-training and post-training correct responses for each knowledge item in a side-by-side format, together with corresponding McNemar

Powered by Editorial Manager® and ProduXion Manager® from Aries Systems Corporation

	p-values. This revision substantially improves interpretability and allows readers to identify the specific knowledge domains that improved after the simulation-based intervention.<br>Comment 8<br>Also, the item-level presentation of responses is informative; however, reporting all response options for each question may reduce clarity. Consider simplifying the table to present the proportion of correct responses for each item (pre- and post-training), which would more directly reflect knowledge acquisition. Detailed response distributions could be included as supplementary material if needed.<br>Response: We agree with the reviewer's suggestion. The item-level knowledge table was simplified to report only the proportion of correct responses before and after training for each question, rather than displaying all response options. This improved readability and allowed the table to more directly reflect declarative knowledge acquisition following the intervention. Detailed response distributions were removed from the main manuscript.<br>Comment 9<br>In the section "transfer of learning outcomes", the results are reported thought these are difficult to interpret. In particular, it is unclear how the proportions in the table were derived and the denominator used. The total number of urethral catheterisations performed collectively is reported as 274, however, it would be helpful to include the distribution of procedures per medical intern (e.g., range and/or median with IQR) to better understand variability in individual procedural exposure within the six months post training.<br>Response: The "Transfer of learning outcomes" section and accompanying tables were revised extensively to improve clarity and interpretability. Specifically, explanatory footnotes were added to clarify the denominators used for each percentage calculation:<br>•<br>procedure outcome percentages were calculated based on the total number of catheterization attempts,<br>•<br>causes of failed attempts were calculated relative to total failed attempts,<br>•<br>complication percentages were calculated relative to total complications.<br>This clarification has now been explicitly stated in the revised table footnotes and Results section.<br>Comment 10<br>It would be helpful to include the distribution of procedures per medical intern (e.g., range and/or median with IQR) to better understand variability in individual procedural exposure.<br>Response: In response, additional descriptive data regarding procedural exposure were added to the Results section. We now report that the median number of urethral catheterizations performed per intern during the six-month follow-up period was 5 [IQR: 4–10], demonstrating variability in individual clinical exposure following the training intervention.<br>We again sincerely thank the reviewer for the insightful comments and recommendations, which significantly improved the quality, clarity, and scientific presentation of our manuscript.<br>Sincerely,<br>Ramy Touma Sawaya, MD<br>(on behalf of all coauthors
**Additional Information:**	
**Question**	**Response**
**Financial Disclosure**<br><br>Enter a financial disclosure statement that describes the sources of funding for the work included in this submission. Review the submission guidelines for detailed requirements. View published research articles from *PLOS ONE* for specific	The author(s) received no specific funding for this work.

examples.<br><br>This statement is required for submission and **will appear in the published article** if the submission is accepted. Please make sure it is accurate.<br><br>**Funded studies**<br>Enter a statement with the following details:<br>• Initials of the authors who received each award<br>• Grant numbers awarded to each author<br>• The full name of each funder<br>• URL of each funder website<br>• Did the sponsors or funders play any role in the study design, data collection and analysis, decision to publish, or preparation of the manuscript?<br><br>Did you receive funding for this work?	
**Competing Interests**<br><br>Use the instructions below to enter a competing interest statement for this submission. On behalf of all authors, disclose any competing interests that could be perceived to bias this work—acknowledging all financial support and any other relevant financial or non-financial competing interests.<br><br>This statement is required for submission and **will appear in the published article** if the submission is accepted. Please make sure it is accurate and that any funding sources listed in your Funding Information later in the submission form are also declared in your Financial Disclosure statement.<br><br>View published research articles from *PLOS ONE* for specific examples.	The authors have declared that no competing interests exist.

Powered by Editorial Manager® and ProduXion Manager® from Aries Systems Corporation

**NO authors have competing interests**<br><br>Enter: *The authors have declared that no competing interests exist.*<br><br>**Authors with competing interests**<br><br>Enter competing interest details beginning with this statement:<br><br>*I have read the journal's policy and the authors of this manuscript have the following competing interests: [insert competing interests here]*<br><br>* typeset	
**Data Availability**<br><br>Authors are required to make all data underlying the findings described fully available, without restriction, and from the time of publication. PLOS allows rare exceptions to address legal and ethical concerns. See the PLOS Data Policy and FAQ for detailed information.<br><br>A Data Availability Statement describing where the data can be found is required at submission. Your answers to this question constitute the Data Availability Statement and **will be published in the article**, if accepted.<br><br>**Important:** Stating 'data available on request from the author' is not sufficient. If your data are only available upon request, select 'No' for the first question and explain your exceptional situation in the text box.<br><br>Do the authors confirm that all data underlying the findings described in their manuscript are fully available without restriction?	No - some restrictions will apply
Additional data availability information:	
**Ethics Statement**<br><br>Enter an ethics statement for this submission. This statement is required if	USEK IRB<br>In response to your request, and after meeting of the Ethics Committee<br>On 01/07/2020 and discussion of your project (E111518), the decision taken is Favorable

the study involved:<br><br>• Human participants<br>• Human specimens or tissue<br>• Vertebrate animals or cephalopods<br>• Vertebrate embryos or tissues<br>• Field research<br><br>Write "N/A" if the submission does not require an ethics statement.<br><br>General guidance is provided below. Consult the submission guidelines for detailed instructions. **Make sure that all information entered here is included in the Methods section of the manuscript.**	Written consent via online form

Powered by Editorial Manager® and ProduXion Manager® from Aries Systems Corporation

**Format for specific study types**<br><br>**Human Subject Research (involving human participants and/or tissue)**<br>• Give the name of the institutional review board or ethics committee that approved the study<br>• Include the approval number and/or a statement indicating approval of this research<br>• Indicate the form of consent obtained (written/oral) or the reason that consent was not obtained (e.g. the data were analyzed anonymously)<br><br>**Animal Research (involving vertebrate animals, embryos or tissues)**<br>• Provide the name of the Institutional Animal Care and Use Committee (IACUC) or other relevant ethics board that reviewed the study protocol, and indicate whether they approved this research or granted a formal waiver of ethical approval<br>• Include an approval number if one was obtained<br>• If the study involved *non-human primates*, add *additional details* about animal welfare and steps taken to ameliorate suffering<br>• If anesthesia, euthanasia, or any kind of animal sacrifice is part of the study, include briefly which substances and/or methods were applied<br><br>**Field Research**<br><br>Include the following details if this study involves the collection of plant, animal, or other materials from a natural setting:<br>• Field permit number<br>• Name of the institution or relevant body that granted permission	
**Data Availability**<br><br>Before publication, Authors are required to make fully available and without restriction all data underlying their findings. Sharing the data underlying your findings increases the visibility, credibility, and impact of your work, while supporting transparency and future scientific discovery. Please see our PLOS Data	All relevant de-identified data supporting the findings of this study are publicly available in the BioStudies repository hosted by the European Bioinformatics Institute under accession number S-BSST2802. The dataset can be accessed at: https://www.ebi.ac.uk/biostudies/studies/S-BSST2802

Powered by Editorial Manager® and ProduXion Manager® from Aries Systems Corporation

Policy page for detailed information on this policy.<br><br>A **Data Availability Statement**, detailing where the data can be accessed, is required at first submission. Insert your Data Availability Statement in the box below.<br><br>Please see the data reporting section of our submission guidelines for instructions on what you need to include in your Data Availability Statement. Examples of acceptable Data Availability Statements are included here for reference:<br>1. The minimal data set [and accompanying code, where generated] is available at [repository name] via [DOI hyperlink]<br>2. No data was generated by this study. The following existing data sources were used: [dataset name] from [dataset location] available via [web address or DOI hyperlink].<br>3. For Study Protocols: No datasets were generated or analysed during the current study. All relevant data from this study will be made available upon study completion.<br><br>*PLOS allows rare exemptions to address legal and ethical concerns. If you have legal or ethical restrictions, please use the box below to detail these in full sentences for the Journal team to consider.*<br><br>This statement will be typeset if the manuscript is accepted for publication.	

Manuscript

Click here to access/download;Manuscript;Foley MedEd Manuscript (1) (1) - Clean.docx

Enhancing medical intern competence in urethral catheterization: impact of simulation-based training on knowledge, self-efficacy, and clinical outcomes.

Evaluating urinary catheterization simulation training for new interns

Authors: Maher Abdessater[1,2], Anthony Kanbar[3], Joey El Khoury[3], Rami Halabi[3], Ramy Touma Sawaya[2*], Raghid El Khoury[3]*

Institutional affiliations: 1: Doctoral College, Holy Spirit University of Kaslik (USEK), Jounieh, Lebanon

2: Division of Urology, Department of Surgery, American University of Beirut Medical Center, Beirut, Lebanon.

3. School of medicine and medical sciences, Holy Spirit University of Kaslik (USEK), Jounieh, Lebanon

**Corresponding authors: Maher Abdessater, ma718@aub.edu.lb; Ramy Touma Sawaya, rt81@aub.edu.lb*

Declarations:

Ethical considerations: The training curriculum was reviewed and approved by the ethical committee of the university medical center with no specific concerns.

Consent to participate and publish signed during study explanation and prior to enrollment.

Availability of data and materials: All relevant de-identified data supporting the findings of this study are publicly available in the BioStudies repository hosted by the European Bioinformatics Institute under accession number S-BSST2802. The dataset can be accessed at: https://www.ebi.ac.uk/biostudies/studies/S-BSST2802

Funding: None

Author contributions: AK wrote the manuscript. JK and RH conducted training sessions. RK, AK guided the analysis. MA supervised the manuscript writing and the interpretations. RK, AK supervised the project. RTS, MA, and AK performed the statistical analysis. All authors read and approved the final manuscript.

Acknowledgements: We thank Dr Raghid El Khoury, Dr Antoine Kassis and Dr Rodrigue Saad who had important contributions and whose support was instrumental to the success of the training sessions.

Abbreviations: UC: urinary catheterization; PSE: perceived self-efficacy; CHUNDS: Notre Dame des Secours University Hospital Center; USEK: Holy Spirit University of Kaslik; OSATS: Objective Structured Assessment of Technical Skill; SD: standard deviation

Abstract

Introduction: Urethral catheterization is a common procedure, yet most related complications occur when performed by new medical interns. Studies have shown inadequate training and knowledge among junior doctors. This study evaluated the declarative knowledge of new interns regarding urethral catheterization and assessed the effectiveness of a simulation-based urology session in improving their knowledge, confidence, and perceived self-efficacy.

Methods: A one-day urology simulation workshop was conducted for 40 new interns at a university hospital. Participants completed a pre-session online questionnaire assessing demographics, urinary catheterization knowledge, and self-reported confidence and self-efficacy. The workshop included three modules: an interactive presentation, instructional videos, and hands-on simulation on male and female models. Post-session assessments measured changes in knowledge, confidence, and self-efficacy scores using standardized evaluation tools.

Results: Forty new medical interns participated. Only 15% felt adequately prepared before training. Knowledge gaps were identified regarding catheter types and sizes (92.5%), optimal patient positioning (62%), proper meatus exposure, and balloon inflation technique (100%). Following the workshop, mean knowledge scores increased from 7.7±1.24 to 12.2±1.11, self-reported confidence from 6.60±1.79 to 8.66±0.94, and perceived self-efficacy from 3.32±0.94 to 4.32±0.62 (all $p<0.001$). At six-month follow-up, participants collectively

performed 274 urethral catheterizations with a 93% success rate and a low complication rate of 4% (n=11), reflecting effective transfer of learning from the simulation to clinical practice.

Discussion: New medical interns demonstrated insufficient baseline knowledge of urethral catheterization. The structured urology simulation session significantly improved their declarative knowledge, confidence, and perceived self-efficacy, underscoring the value of simulation-based training in early postgraduate medical education.

Keywords: Urinary catheterization; intern; education; simulation

Background

Urethral catheterization (UC) is routinely performed in hospitalized patients[1]. During this procedure, a catheter is inserted into the urethra and advanced through the striated urinary sphincter and bladder neck into the bladder to drain urine. If there is a need for continuous urine drainage, a balloon surrounding the tip of the catheter is inflated in the bladder to hold the catheter proximal to the bladder neck[2]. UC is performed for therapeutic purposes, such as urinary retention[3], bladder emptying or urine output measurement perioperatively or in acutely ill patients[3,4], urinary incontinence, drug delivery, bladder irrigation[5], social and hygiene reasons[3], or diagnostic purposes, such as urodynamic studies, cystogram imaging, collection of urine sample[5]. Apart from clean intermittent self-catheterization, UC is a sterile procedure that requires special knowledge to assess the indications, contraindications, and potential complications, and specific technical skills to expose the urethral meatus properly and align the urethra correctly during the maneuver[6].

Successful UC is operator dependent. Early detection of risk factors for complications in males, such as enlarged prostate and urethral strictures, allows for correct decision-making to prevent UC-related morbidity[7]. Similarly in women, special skills and maneuvers are required when risk factors for difficult catheterization are present, such as morbid obesity, intravaginal retraction of the urethral meatus, and prolapse, to optimize the visualization and access the introitus and urethral meatus[8,9]. Iatrogenic urethral trauma caused by medical intervention occurs in 0.3% of catheterization maneuvers during the insertion process, and is mainly caused by false passage creation or intraurethral balloon inflation and may lead to short-term and long-term morbidities[10,11]. Complications from UC increase hospital stay

by 9.4±10 days and costs by €335,377 over 6 months[10]. Recent efforts are focusing on the identification of strategies to reduce inappropriate catheter use and avoid associated complications in the short-term, such as infection, hematuria, obstructive kidney injury, need for transfusion or cystoscopy, and long-term, such as urethral stricture, need for long-term bladder catheter or self-urethral dilation[12–16].

At the university hospital, UC is performed traditionally by physicians. Newly qualified doctors are expected to safely and confidently perform UC at the end of their training[1]. However, recent literature shows that junior doctors receive inadequate theoretical and practical training concerning UC during their years in medical school[7,17] before their direct involvement in patient care. The rate of medical interns who did not feel prepared to perform this skill varied in the literature between 18.6% and 55.6% among the Irish[7], Scottish[18], French[19], American[20], and Filipino[17] experiences. Most catheterization-related morbidities occur when interns perform UC, especially at the beginning of their internship and when they are unsupervised[1,7]. Gaps were reported in the theoretical knowledge of the steps of UC, and the mechanisms and identification of iatrogenic injuries[17]. On the practical side, nearly half of new interns have not undergone a urology rotation during their undergraduate training[21,22] and have never performed UC. The majority reported a lack of previously supervised catheterization and especially in female patients[21].

UC training programs successfully improved the knowledge of new interns and their confidence level in performing the skill[23,24] and reduced the incidence of iatrogenic injuries. Still, the real benefit of simulation-based training in UC is debatable because the current models may lack the typical challenges encountered in actual patients, such as increased pain, difficult entry to the bladder, and anatomical variations.

Therefore, as part of a plan to reduce catheter-related morbidity in practice, this study was conducted to evaluate new interns' declarative knowledge regarding UC and assess the effectiveness of a urology simulation session in improving their declarative knowledge score, perceived self-efficacy (PSE), and technical skills.

Methods

This study employed a prospective single-group pre–post educational design to evaluate the impact of a simulation-based urethral catheterization workshop on interns' knowledge, technical performance, and perceived self-efficacy.

Participant recruitment

The urology simulation session was conducted for new interns at the University Hospital. This was done at the beginning of their academic year in order to adequately train the interns on proper, safe, and comfortable technique. Inclusion criteria for the study mandated that all participants were first-year interns who graduated from the affiliated medical school the previous year. Furthermore, all participants were not previously part of a urology training or simulation session and had no extra specialized urology-related training aside from their rotations during undergraduate training. All participants had the study thoroughly explained to them and were asked to sign an informed consent prior to enrollment.

Pre-testing

The urology simulation session was a one-day curriculum organized by the urology department to teach participants about UC. Interns were divided into small groups of eight. The simulation session had 2 theoretical and one practical module of twenty minutes each. Prior to starting the educational modules, all participants were asked to fill out an anonymous, online

questionnaire and perform a foley catheter insertion on the available male and female simulation models.

The first section in the questionnaire asked about demographic characteristics such as age, sex, self-reported confidence level on a scale of 1 to 10, previous attempts at inserting a foley catheter, and opinion on preferred method of training for hands-on procedures. The following section included 2 open ended questions asking participants to give the indications and contraindications for UC, and 13 dichotomous or multiple-choice questions with a single correct answer that were inspired by previous similar works. This section assessed medical knowledge related to catheter insertion, such as the types of available catheters and sizes, optimal place to apply lubricant, angle of penis during insertion, depth of catheter insertion, and importance of drained urine volume after insertion. Self-reported confidence in performing urethral catheterization was measured using a numerical rating scale from 1 to 10, where 10 indicated the highest level of confidence. Perceived self-efficacy was evaluated using a 5-point Likert scale reflecting the participant's perceived preparedness to perform urethral catheterization. These items were developed for the purpose of this educational evaluation and are consistent with approaches commonly used in simulation-based medical education studies to capture changes in perceived competence following training. Finally, the perceived self-efficiency (PSE) score reflected interns' beliefs in their competence to perform UC, and involved a self-reflective 5-point Likert scale, for the question "How prepared do you feel to perform UC on a scale from 1 to 5 (where 5 is the highest and 1 is the lowest preparedness levels)?". The questionnaire was reviewed by the teaching urologist involved in the training program to ensure clarity and relevance to the learning objectives.

The knowledge questionnaire included two open-ended questions assessing recognition of indications and contraindications for urethral catheterization, as well as thirteen multiple-

choice or dichotomous questions addressing key procedural and theoretical aspects of catheterization, including catheter types and sizes, patient and operator positioning, sterile technique, exposure of the urethral meatus, lubrication, insertion depth, balloon inflation, and procedural documentation. Each correct response was assigned one point, generating a cumulative declarative knowledge score used for pre- and post-training comparison. The questionnaire items were developed based on previously published educational studies on urethral catheterization and standard urology references, including Campbell-Walsh Urology, to ensure that the content reflected clinically relevant knowledge and procedural steps expected from new medical interns.The first attempt of foley insertion was done under supervision of a senior resident, who utilized the Objective Structured Assessment of Technical Skill (OSATS)[25] rubric to assess the skills and proficiency of each participant. Each participant performed a catheterization attempt before the educational session and again after completion of the training. The procedures were evaluated by senior urology residents supervising the simulation session, who were familiar with the OSATS evaluation tool. Each step in the rubric was assigned one point, for a maximum score of 15. Pre- and post-training OSATS scores were summarized using mean±standard deviation and compared using paired statistical testing to assess improvement in technical performance following the training.

The assessed skills included history taking for indications and contraindications, correct choice of balloon inflation solution, catheter type and material, proper patient and operator positioning, adequate sterilization of the genitals and maintenance of sterile technique during insertion, optimal exposure of urethral meatus, application of lube, penile stretching and angling, catheter insertion, balloon inflation, and urine drainage (Table 1).

Table 1. OSATS rubric for trainee evaluation concerning urethral catheterization

**Skill performance checkpoints**	**Points**
Indication (history) check	1

Contraindications check	1
Choice of catheter type	1
Choice of catheter size	1
Patient positioning	1
Operator positioning	1
Genital cleansing	1
Balloon integrity check	1
Exposure of urethral meatus	1
Urethra alignment	1
Lubricant application	1
Depth of insertion of the catheter	1
Choice of balloon inflation material	1
Verification of the drained urine volume	1
Documentation	1

OSATS=Objective Structured Assessment of Technical Skills. Each checkpoint was assigned 1 point, for a maximum total score of 15.

Training development

The training curriculum was developed based on previous works in literature[7,17]. All teaching sessions were given by an experienced urologist with more than five years of teaching experience. The learning objectives of the simulation session were to educate the participants on the indications, contraindications, and the steps of a correct UC, while also enhancing the technical skills and confidence in their performance of UC.

After the pretest, the first theoretical module involved a short, interactive PowerPoint presentation which highlighted key points in UC: anatomical considerations in males and females, indications, contraindications, catheter types and selection criteria, detailed steps of UC in males and females, complications, and the importance of documentation and seeking help for complex cases. There was also emphasis on the importance of the "one gentle attempt only" rule to reduce the likelihood of iatrogenic false passage[10]. Materials were selected from the Campbell-Walsh Urology, 11th edition[23]. In the second theoretical module, catheter insertion was demonstrated by videos showing the steps of UC with verbal descriptions. Videos were specific to the female and male dummies available at the simulation

center, and were picked from the provider's website[26,27]. Lastly, interns reattempted UC on male and female dummies under the supervision of the senior urology residents, who commented on the technique and outlined the flaws. The senior residents again utilized the OSATS rubric to assess improvement of technical skills.

The workshop was conducted in small groups of approximately eight participants to facilitate interaction and individualized instruction. Each group was supervised by a senior urology resident, resulting in an approximate instructor-to-learner ratio of 1:8 during the simulation exercises. The hands-on simulation module lasted approximately 20 minutes, during which participants practiced urethral catheterization on male and female simulation models. Trainees were encouraged to perform the procedure while verbalizing each step. Feedback was provided immediately after each attempt using a structured, formative approach, in which the supervising resident highlighted correct steps, identified technical errors, and provided practical suggestions for improvement. This immediate feedback aimed to reinforce correct technique and address common procedural mistakes.

Transfer of learning

The educational impact of the simulation was assessed following Kirkpatrick's four-level model. Levels 1 and 2 (reaction and learning) were evaluated through participant satisfaction, knowledge, confidence, and perceived self-efficacy immediately after the session. Levels 3 and 4 (behavioral change and results) were assessed at six-month follow-up through clinical performance indicators, number of catheterizations performed, procedural success rate, and complication rate, providing evidence of transfer of learning into clinical practice[28].

Six months after the simulation, a remote assessment was conducted to evaluate behavioral change (Level 3) and patient-related outcomes (Level 4). Data on the number, success, and complications of catheterizations were collected from interns' clinical logbooks and verified by supervising urology residents. This approach allowed pragmatic evaluation of how simulation-based training translated into real clinical performance.

Procedural success was defined as successful insertion of the urinary catheter with appropriate urine drainage and no need for additional attempts by another operator or alternative catheterization techniques. Complications were defined as any adverse event occurring during or following the catheterization procedure, including urethrorrhagia, false passage creation, urinary tract infection, or the need for suprapubic catheterization. Follow-up data were collected from interns' procedural logbooks during their clinical rotations and were reviewed in collaboration with supervising urology residents and physicians to verify the reported number of procedures, procedural outcomes, and complications.

Statistical analysis

Interns' declarative knowledge and PSE scores were reported using descriptive statistics with means and standard deviations or frequencies and percentages. Pre- and post-training scores were compared using paired t-tests. Although perceived self-efficacy and confidence were measured using Likert-type scales, these outcomes were treated as approximately continuous variables and summarized using means and standard deviations, a commonly accepted approach in educational research for within-subject comparisons. Effect sizes with corresponding 95% confidence intervals were also reported to facilitate

interpretation of the magnitude of observed changes. Statistical significance was defined as $p < 0.05$.

Data from surveys were collected using an online form. The analysis was conducted using RStudio 2021.09.0+351 "Ghost Orchid" Release[29]. The inclusion of six-month outcome data permitted assessment of behavioral change and patient-level results (Kirkpatrick Levels 3 and 4), expanding the evaluation beyond knowledge and self-efficacy gains.

Ethical considerations

This study was reviewed and approved by the Ethical Review Board of the Holy Spirit University of Kaslik (USEK), Jounieh, Lebanon (Approval number: EN-CETH-001). All participants were informed about the objectives of the study prior to enrollment. Written informed consent was obtained from all participants before participation. The study procedures were conducted in accordance with the ethical principles outlined in the Declaration of Helsinki.

Results

Participant demographics

The study initially recruited 44 interns, however, 4 were not included as they had previously participated in general simulation sessions that included foley catheterization during their undergraduate training.

40 new medical interns participated in the urology simulation session, of which 35% (n=14) were males and 65% (n=26) were females. All had completed five years of studies at the same medical school. At the time of the training, only 15% (n=6) felt prepared correctly (Table 2).

Table 2. Study participants' demographic characteristics

Variable	Characteristics	N (%)
Age (years)	25-26	40 (100%
Sex	Males	14 (35%)
	Females	26 (65%)
Consent to participate	Yes	40 (100%)
	No	0 (0%)

Percentages are calculated based on the total number of participants (N = 40).

Participant initial knowledge

All participants considered history taking and contraindications recognition essential before UC and were able to identify at least 3 indications, such as urinary drainage during retention, control of incontinence, and bladder irrigation, and contraindications, such as urethral trauma, acute infection, and known bladder tumors. The majority (92%) did not know the difference between catheter types and sizes. Only 38% (n=15) would position the patient supine for catheterization, and 43% (n=17) would stand at the correct patient side, which is the side of the operator's dominant hand. All of them agreed that the procedure should be sterile. Most participants did not know how to correctly expose the urethral meatus (75%, n=30), the importance of the solution used to inflate the balloon (100%, n=40), or that lubricant must be applied into the urethra (25%, n=10). In contrast, most interns (83%, n=33) knew that the penis should be stretched perpendicular to the body during catheterization, and that the catheter had to be introduced to the hub (65%, n=26). The majority agreed on

the importance of documentation (85%, n=34) and assessment of the volume drained (90%, n=36) (Table 3 and further detailed in Supp Table 1).

Table 3. Pre- and post-test interns' declarative knowledge concerning urethral catheterization

Questions	Pre-training correct n(%)	post-training correct n(%)	McNemar p-value
**What are the indications for UC?**	40 (100)	40 (100)	-
**What are the contraindications for UC?**	40 (100)	40 (100)	-
**Do you know the difference between catheter types?**	3 (8)	34 (85)	**<0.001**
**Are you able to correctly choose the catheter size?**	4 (8)	31 (77.5)	**<0.001**
**What is the best patient position during UC?**	15 (38)	40 (100)	**<0.001**
**Where is the best position of the operator who is performing the UC?**	17 (43)	17 (42.5)	-
**Should the UC be a sterile procedure?**	40 (100)	40 (100)	-
**Should the balloon be checked before UC?**	1 (3)	25 (62.5)	**<0.001**
**Do you know how the correctly expose the urethral meatus in males and females?**	10 (25)	10 (25)	-
**At which angle should the penis be stretched during UC?**	33 (83)	33 (83)	-
**Where is the lubricant best applied before UC?**	25 (63)	33 (82.5)	**<0.001**
**What is the level or depth of UC insertion?**	26 (65)	35 (87.5)	**0.004**
**Is the type of solution used to inflate the balloon important?**	0 (0)	37 (92.5)	**<0.001**
**Is the volume of urine drained important?**	36 (90)	36 (90)	-
**Is procedure documentation important?**	34 (85)	37 (92.5)	0.453
**Values are presented as n (%)**			

Variation in knowledge, PSE, and confidence levels

The pre- and post-training assessments showed an increase in the mean declarative knowledge score from 7.7±1.24 to 12.2±1.11, and in the mean PSE score, which increased from 3.32±0.94 to 4.32±0.62. Prior to training, the self-reported confidence levels (SRCL)

ranged from 1 to 10, with a median of 7 and a mean of 6.6±1.79. After the training session, the levels varied exclusively between 7 and 10. The mean confidence level was 8.66±0.94, with a median of 9. All the improvements showed a statistical significance (p<0.001) (Table 4).

Table 4. Changes in the OSATS scores for TS and NTS before and after training.

	Before training	After training	P-value
**TS scores**	**8.9±2.1**	**12.8±2.3**	**<0.01**
**NTS scores**	**6.8±2.2**	**7.2±3.1**	**0.4**

TS=technical skills; NTS=non-technical skills; OSATS=Objective Structured Assessment of Technical Skills.
Values are presented as mean±standard deviation.
P-values were calculated using paired statistical testing comparing scores before and after training.

Participant satisfaction

Prior to the training, participants were asked to self-report their readiness to perform UC in the hospital, resulting in 34 (85%) feeling unprepared to perform the procedure. The most common teaching modalities among the new interns were live simulation (n=20, 53%) followed by supervised bedside teaching (n=18, 47%) (Table 5a). At the end of the training, the survey showed that 92.5% (n=37) of the trainees were very satisfied. All (n=40) rated the quality of the training as good and 97.5% (n=39) reported that the training met their needs and that the skills acquired were useful in their daily work. Almost all trainees (97.5%, n=39) stated that they would recommend this training to others The magnitude of the improvements is reflected by the mean differences and their 95% confidence intervals, indicating substantial gains in knowledge, perceived self-efficacy, and confidence following the training session (Table 5b).

Table 5a. Pre-test participant preparedness and preferred method of learning

Feeling adequately prepared for UC*	Yes	6 (15%)
	No	34 (85%)
Preferred method of training	Live simulation demonstration	20 (53%)
	Supervised bedside catheterization	18 (47%)
	Video	0 (0%)
	Conference	0 (0%)

UC=Urethral catheterization
Percentages are calculated based on the total number of participants (N = 40).

Table 5b. Changes in the mean PSE, self-reported confidence level, and declarative knowledge scores before and after training.

	**Before training**	**After training**	**Mean of difference [95% confidence interval]**	**P-value**
Mean declarative knowledge score	7.7±1.24	12.2±1.11	4.5 [4.07-4.92]	<0.05
Mean PSE score	3.32±0.94	4.32±0.62	1 [0.65-1.36]	<0.05
Self-reported confidence level	6.60±1.79	8.66±0.94	2.05 [1.39-2.72]	<0.05

PSE=perceived self-efficacy.
Values are presented as mean ± standard deviation.
Mean differences (effect size) are reported with 95% confidence intervals.
P-values were calculated using paired statistical testing comparing scores before and after training.

Transfer of learning outcomes

Over 6 months, interns collectively performed 274 urinary catheterizations, reflecting sustained application of learned skills in clinical settings (Level 3). The median number of procedures performed per intern was 5 [IQR: 4–10], demonstrating variability in individual procedural exposure. The overall procedural success rate was 93%, with a low complication rate of 4% (Level 4), indicating that simulation training positively influenced both behavior and patient outcomes. The most common causes of failure were urethral meatal stenosis in 32% (n=6) and difficulty in identifying the urethral meatus in women in 32% (n=6). Other reported causes were inadequate exposure of the genitals due to patient disability (e.g., hip

fracture) in 26% (n=5) and phimosis in male patients in 10% (n=2). Overall, 4% (n=11) were complicated (Table 6).

Table 6: participant satisfaction towards the boot camp

Domain	Answer	n	%
**Satisfaction**	Very well	37	92.5%
	Ok	3	7.5%
**Quality of training**	Very well	40	100%
	Ok	0	0%
**Meeting the needs of trainees**	Very well	39	97.5%
	Ok	1	2.5%
**Usefulness of the skills acquired in daily practice**	Yes	39	97.5%
	No	1	2.5%
**Recommendation of the exercise to other trainees**	Yes	39	97.5%
	No	1	2.5%

Percentages are calculated based on the total number of participants (N=40).
Responses were collected through a post-training satisfaction questionnaire.

The most common complication was urethrorrhagia with spontaneous resolution in 55% (n = 6) of cases. This was followed by the occurrence of a false passage in 18% (n=2), a urinary tract infection in 18% (n=2) and the need for suprapubic catheterization in 9% (n=1) (Table 7).

Table 7. Rate and causes of urinary catheterization failure and complications.

Description	n	%
**Total UC performed**	274	100
**Failed attempts**	**19**	**7%**
**Reasons for failed attempts (n=19)**		
**Difficulty identifying urethral meatus (Female)**	6	32%
**Phimosis (Male)**	2	10%
**Inadequate exposure of the genitals**	5	26%
**Stenosis of the urethral meatus**	6	32%
**Complications**	**11**	**4%**
**Urethrorrhagia with spontaneous resolution**	6	55%
**False passage creation**	2	18%
**Urinary tract infection**	2	18%
**Need for suprapubic catheterization**	1	9%

UC=urethral catheterization.
% for procedure outcomes are based on total catheterisation attempts (n=274)
% for reasons for failed attempts are based on total failed attempts (n=19)
% for complications are based on total complications (n=11)

Discussion

The primary purpose of the simulation session was to bridge the gap between the knowledge gained in medical school and the technical skills needed in clinical practice. Based on the previous literature showing that new interns caused higher UC-related morbidity[1], this training was organized at the beginning of the internship with the aim of teaching new interns the essential steps and basic clinical skills for UC and allowing them to practice on dummies before catheterizing real patients, which may help support the development of confidence and competence in performing UC[17].

The self-assessment questionnaire administered before the training helped the trainees identify their weaknesses, strengths, knowledge, and PSE regarding UC. Only 15 % of the new interns felt adequately prepared to perform UC. The rate was lower than what was reported in the literature by Manalo et al. (55.6%)[17], Thomas et al. (36% and 52%)[7], and Bigot et al. (26% and 38.3%)[19], which raises the need to focus on procedural skill teaching in medical school and emphasizes the importance of the simulation session organized. All our interns (100%) were aware of the importance of history-taking before proceeding to catheterization, in contrast to 23.6%[17] and 26.7%[30] reported in previous studies by Manalo et al. and Browne et al, respectively. This step is of great importance because it allows the recognition of risk factors of complications[17]. More than half of the participants (63%) knew adequately that the lubricant should be applied inside the urethra, despite the tendency to apply it to the catheter, as reported in 97.8% of cases in the Manalo et al. study[17]. Most of our interns were aware of the importance of documentation (85%) and assessment of the

volume drained (90%), in contrast with the low rates of documentation (only in 29% of patients with an indwelling catheter) reported by Cornia et al.[31]

The training focused on the weaknesses identified by the initial survey regarding catheter types and sizes, patient positioning during catheterization, operator positioning at the bedside, correct exposure of the urethral meatus, and the importance of the solution used to inflate the balloon. The short learning modules of 20 minutes each was efficient to increase knowledge retention while maintaining attention and focus. The division of interns into small groups of 8 individuals helped optimize the learning experience without excessive crowding and time restrictions. Also, the incorporation of different methods in the training, such as a didactic lecture, video-based demonstration, and hands-on simulation, maximized the teaching experience, as demonstrated by previous works[32,33]. Despite being underreported in various urology curricula, simulation-based learning has demonstrated its efficacy in urology training[32,34]. Participation in the simulation session was associated with an increase in declarative knowledge and PSE scores among the new interns concerning UC. Similarly, Waters et al. demonstrated the beneficial effects of interactive teaching of UC on the confidence level and examination scores[32]. Browne et al. also reported a beneficial effect of the structured practical training on the SRCL of new interns on a different categorical scale[30].

The inclusion of a six-month follow-up assessment extends this study beyond Kirkpatrick Level 2, demonstrating measurable behavioral change and patient-related outcomes. The high procedural success and low complication rates observed during follow-up provide descriptive insight into interns' early clinical experience with catheterization. However, these observational data should be interpreted cautiously, as ongoing clinical

exposure, supervision, and repeated procedural practice during the internship period may also have influenced these outcomes.

The originality of this study remains in its timing, clear methodology, and objective outcome assessment. The academic timing of the simulation session just before the beginning of the internship both boosted learning capabilities for the participants while also reducing bias in participant experience. At the time of the simulation session, interns would have completed medical school and acquired sufficient medical background to understand the anatomical and physiological basis needed for UC, however, as shown in the results, few of them have ever performed UC on patients. The timing proposed in this study differed from the previous work done by Waters et al., where teaching targeted second-year medical students[32]. To facilitate the assimilation of the UC, there was focus on the essential steps using the standard two-way catheters, without much complicated discussion of the other specific aspects of UC, such as three-ways catheterization, bladder washout, and continuous bladder irrigation, like Browne et al.[30] because those topics were considered more specific to urology residents. The impact of the training session was assessed using an objective declarative knowledge score that was calculated before and after the training, without exclusive reliance on the self-reported outcome such as the perceived-self efficacy and SRCL in this study as in previous works in the literature[30].

This study had several limitations. First, The study did not include a parallel control group, as all new interns at the university were required to attend the simulation session as part of their induction curriculum. Although the pre-test served as a within-subject baseline, future studies should adopt a controlled or randomized design comparing simulation-based teaching with traditional instruction to better delineate causal effects. Second, the effect of

the training on UC success and complications in clinical practice on real patients was not observed by the evaluators in the simulation session. Instead, at this stage, there was more reliance on self-reported parameters and knowledge. Third, the small population size, while representative of the population of medical graduates working as new interns, may be a limiting factor for larger programs that incorporate hundreds of new interns every year, emphasizing the importance of this study to be redone in different settings such as university hospitals with larger intern bodies. Also, given the single-group pre–post design of this study, the observed improvements in knowledge, confidence, and clinical performance cannot be attributed exclusively to the simulation session. During the six-month follow-up period, interns were also exposed to routine clinical practice, supervision from senior residents and attending physicians, and repeated procedural experience, which may have contributed to the observed outcomes. Therefore, the results should be interpreted as an association between participation in the training program and improved outcomes rather than a definitive causal relationship. Finally, the study method used simulation with peer feedback, which is considered superior to other conventional learning styles such as lectures, videos, and demonstrations. However, metric-dependent proficiency-based progression training is recently considered the most efficient and performant way to optimize the learning experience[32]. As such, this study may further benefit as a steppingstone to assess the use of metric-dependent proficiency-based progression in different surgical specialties and especially in urology.

Conclusion

Junior doctors have insufficient knowledge regarding UC. Participation in the urology simulation session was associated with improvements in declarative knowledge scores, SRCL, and perceived self-efficacy regarding UC among new interns, showing promise in improving technical skills and confidence for newly graduated interns being introduced into medical practice. Future studies using controlled or randomized designs would be valuable to better isolate the specific impact of simulation-based training on clinical outcomes.

Lessons for Practice

- Simulation-based training for urethral catheterization may help bridge the gap between theoretical knowledge and clinical performance among new medical interns.
- Early, structured procedural training during internship orientation may contribute to improvements in interns' confidence, technical proficiency, and patient safety outcomes.
- Incorporating simulation sessions into intern induction programs supports sustainable improvements in procedural success rates and reductions in catheter-related complications.

References

1. Bhatt NR DN Quinlan MR, et al. A prospective audit on the effect of training and educational workshops on the incidence of urethral catheterization injuries. *Can Urol Assoc J*. 2017;11(7):E302-E306. doi:10.5489/cuaj.4271

2. Tan E AA Kankam H, Menezes P. Urinary catheterization 2: technique and managing failure. *Br J Hosp Med*. 2019;80(9):C136-C138. doi:10.12968/hmed.2019.80.9.C136

3. Tan E AA Kankam H, Menezes P. Urinary catheterization 1: indications. *Br J Hosp Med*. 2019;80(9):C133-C135. doi:10.12968/hmed.2019.80.9.C133

4. Clayton JL. Indwelling Urinary Catheters: A Pathway to Health Care–Associated Infections. *AORN Journal*. 2017;105(5):446-452. doi:10.1016/j.aorn.2017.02.013

5. Wilson M. Causes and management of indwelling urinary catheter-related pain. *Br J Nurs*. 2008;17(4):232-239. doi:10.12968/bjon.2008.17.4.28712

6. Haider MZ AP. Bladder Catheterization. In: StatPearls. *StatPearls Publishing*. Published online 2022.

7. Thomas AZ GS Meagher D, Creagh T. Avoidable iatrogenic complications of urethral catheterization and inadequate intern training in a tertiary-care teaching hospital. *BJU Int*. 2009;104(8):1109-1112. doi:10.1111/j.1464-410X.2009.08494.x

8. Ghaffary C YA Villanueva C, Leslie SW. A Practical Approach to Difficult Urinary Catheterizations. *Curr Urol Rep*. 2013;14(6):565-579. doi:10.1007/s11934-013-0364-3

9. Willette P CS. Current Trends in the Management of Difficult Urinary Catherizations. *WestJEM*. 2012;13(6):472-478. doi:10.5811/westjem.2011.11.6810

10. Davis NF QM Bhatt NR, et al. Incidence, Cost, Complications and Clinical Outcomes of Iatrogenic Urethral Catheterization Injuries: A Prospective Multi-Institutional Study. *J Urol*. 2016;196(5):1473-1477. doi:10.1016/j.juro.2016.05.114

11. Croghan SM HL O'Connor EM, et al. A Prospective Multi-Institutional Evaluation of Iatrogenic Urethral Catheterization Injuries. *Journal of Investigative Surgery*. 2022;35(10):1761-1766. doi:10.1080/08941939.2022.2109226

12. Knoll BM WD Ellingson L, et al. Reduction of Inappropriate Urinary Catheter Use at a Veterans Affairs Hospital Through a Multifaceted Quality Improvement Project. *Clinical Infectious Diseases*. 2011;52(11):1283-1290. doi:10.1093/cid/cir188

13. Fakih MG. Reducing Inappropriate Urinary Catheter Use: A Statewide Effort. *Arch Intern Med*. 2012;172(3):255. doi:10.1001/archinternmed.2011.627

14. Niederhauser A ZS Marschall J, Schwappach DL. Nurses' and Physicians' Perceptions of Indwelling Urinary Catheter Practices and Culture in Their Institutions. *J Patient Saf*. 2020;16(2):e82-e89. doi:10.1097/PTS.0000000000000502

15. Meddings J RM Krein SL, Fakih MG, Olmsted RN, Saint S. Reducing unnecessary urinary catheter use and other strategies to prevent catheter-associated urinary tract infection: an integrative review. *BMJ Qual Saf*. 2014;23(4):277-289. doi:10.1136/bmjqs-2012-001774

16. Murphy C FM Prieto J. Interventions to minimise the initial use of indwelling urinary catheters in acute care: A systematic review. *International Journal of Nursing Studies*. 2014;51(1):4-13. doi:10.1016/j.ijnurstu.2012.12.007

17. Manalo M LM Buckley BS. Medical interns' knowledge and training regarding urethral catheter insertion and insertion-related urethral injury in male patients. *BMC Med Educ*. 2011;11(1):73. doi:10.1186/1472-6920-11-73

18. Grimes N LJ McKay A, McIlhenny C. Foundation Year 1 doctors' experience and confidence in catheterisation: A multicentre survey. *Journal of Clinical Urology*. 2019;12(5):380-384. doi:10.1177/2051415819854924

19. Bigot P RM Orsat M, et al. Évaluation des compétences pratiques en fin de deuxième cycle des études médicales : exemple du drainage du bas appareil urinaire. *Progrès en Urologie*. 2008;18(2):125-131. doi:10.1016/j.purol.2007.10.003

20. Vigran H, Krischak M, Khoei A, Wan J, Romo PB, Hadj-Moussa M. Efficacy of a Targeted Foley Catheter Education Program. *Journal of Surgical Education*. 2025;82(9):103578. doi:10.1016/j.jsurg.2025.103578

21. Cetti R SR Bissell L, Shaw R. The urological foot soldier: are we equipping our foundation-year doctors? *The Bulletin of the Royal College of Surgeons of England*. 2010;92(8):284-287. doi:10.1308/147363510X12779829582415

22. Nic An Ríogh AU OC Lonergan PE, Davis NF. Designing and assessing a urethral catheter skills workshop for intern doctors in a university teaching hospital. *Ir J Med Sci*. 2020;189(4):1501-1506. doi:10.1007/s11845-020-02240-4

23. Tailly T DJD. Fundamentals of Urinary Tract Drainage. In: *Campbell-Walsh Urology.* 11th ed. Elsevier; 2016:119-124.

24. Mubarak M IQ Alsaeed M, Alalawi M. Transurethral Catheterization in Early Training: The Impact of Peer-Led Mentorship. *Surgery Research and Practice*. 2021;2021:1-6. doi:10.1155/2021/8498835

25. Martin JA, Regehr G, Reznick R, et al. Objective structured assessment of technical skill (OSATS) for surgical residents. *Br J Surg*. 1997;84(2):273-278. doi:10.1046/j.1365-2168.1997.02502.x

26. Limbs LT. Female Catheterization Trainer. *Limbs & Things*. Published online 2021.

27. Limbs LT. Male Catheterization Trainer. *Limbs & Things*. Published online 2021.

28. Kirkpatrick D. Techniques for evaluation training programs. Published online 1959.

29. R Core Team. R : A Language and Environment for Statistical Computing. *R Foundation*. Published online 2021.

30. Browne C NS Nolan JM, et al. The impact of a structured clinical training course on interns' self-reported confidence with core clinical urology skills. *Ir J Med Sci*. 2018;187(1):255-260. doi:10.1007/s11845-017-1616-x

31. Cornia PB AJ Fraser S, Saint S, Lipsky BA. Computer-based order entry decreases duration of indwelling urinary catheterization in hospitalized patients. *Am J Med*. 2003;114(5):404-407. doi:10.1016/s0002-9343(02)01568-1

32. Waters PS MT Kelly BD, et al. The acquisition and retention of urinary catheterisation skills using surgical simulator devices: teaching method or student traits. *BMC Med Educ*. 2014;14:264. doi:10.1186/s12909-014-0264-3

33. Lehmann R SA Bosse HM, Lutz T, Huwendiek S. Student perceptions of a video-based blended learning approach for improving pediatric physical examination skills. *Ann Anat*. Published online 2016.

34. Ballouhey Q CJ Lescure V, Clermidi P, Romain J, Guigonis V. Simulation et rétention des acquisitions : application au drainage vésical. *Prog Urol*. 2015;25(9):516-522.

Click here to access/download
Supporting Information
FoleyTables and Figures (1).docx

Click here to access/download
Supporting Information
PLOS Authorship Changes Form (2026) signed.pdf

Enhancing medical intern competence in urethral catheterization: impact of simulation-based training on knowledge, self-efficacy, and clinical outcomes.

Evaluating urinary catheterization simulation training for new interns

Authors: Maher Abdessater[1,2], Anthony Kanbar[3], Joey El Khoury[3], Rami Halabi[3], Ramy Touma Sawaya[2*], Raghid El Khoury[3]*

Institutional affiliations: 1: Doctoral College, Holy Spirit University of Kaslik (USEK), Jounieh, Lebanon

2: Division of Urology, Department of Surgery, American University of Beirut Medical Center, Beirut, Lebanon.

3. School of medicine and medical sciences, Holy Spirit University of Kaslik (USEK), Jounieh, Lebanon

**Corresponding authors: Maher Abdessater, ma718@aub.edu.lb; Ramy Touma Sawaya, rt81@aub.edu.lb*

Declarations:

Ethical considerations: The training curriculum was reviewed and approved by the ethical committee of the university medical center with no specific concerns.

Consent to participate and publish signed during study explanation and prior to enrollment.

Availability of data and materials: All relevant de-identified data supporting the findings of this study are publicly available in the BioStudies repository hosted by the European Bioinformatics Institute under accession number S-BSST2802. The dataset can be accessed at: https://www.ebi.ac.uk/biostudies/studies/S-BSST2802

Funding: None

Author contributions: AK wrote the manuscript. JK and RH conducted training sessions. RK, AK guided the analysis. MA supervised the manuscript writing and the interpretations. RK, AK supervised the project. RTS, MA, and AK performed the statistical analysis. All authors read and approved the final manuscript.

Acknowledgements: We thank Dr Raghid El Khoury, Dr Antoine Kassis and Dr Rodrigue Saad who had important contributions and whose support was instrumental to the success of the training sessions.

Abbreviations: UC: urinary catheterization; PSE: perceived self-efficacy; CHUNDS: Notre Dame des Secours University Hospital Center; USEK: Holy Spirit University of Kaslik; OSATS: Objective Structured Assessment of Technical Skill; SD: standard deviation

Abstract

Introduction: Urethral catheterization is a common procedure, yet most related complications occur when performed by new medical interns. Studies have shown inadequate training and knowledge among junior doctors. This study evaluated the declarative knowledge of new interns regarding urethral catheterization and assessed the effectiveness of a simulation-based urology session in improving their knowledge, confidence, and perceived self-efficacy.

Methods: A one-day urology simulation workshop was conducted for 40 new interns at a university hospital. Participants completed a pre-session online questionnaire assessing demographics, urinary catheterization knowledge, and self-reported confidence and self-efficacy. The workshop included three modules: an interactive presentation, instructional videos, and hands-on simulation on male and female models. Post-session assessments measured changes in knowledge, confidence, and self-efficacy scores using standardized evaluation tools.

Results: Forty new medical interns participated. Only 15% felt adequately prepared before training. Knowledge gaps were identified regarding catheter types and sizes (92.5%), optimal patient positioning (62%), proper meatus exposure, and balloon inflation technique (100%). Following the workshop, mean knowledge scores increased from 7.7±1.24 to 12.2±1.11, self-reported confidence from 6.60±1.79 to 8.66±0.94, and perceived self-efficacy from 3.32±0.94 to 4.32±0.62 (all $p<0.001$). At six-month follow-up, participants collectively

performed 274 urethral catheterizations with a 93% success rate and a low complication rate of 4% (n=11), reflecting effective transfer of learning from the simulation to clinical practice.

Discussion: New medical interns demonstrated insufficient baseline knowledge of urethral catheterization. The structured urology simulation session significantly improved their declarative knowledge, confidence, and perceived self-efficacy, underscoring the value of simulation-based training in early postgraduate medical education.

Keywords: Urinary catheterization; intern; education; simulation

Background

Urethral catheterization (UC) is routinely performed in hospitalized patients[1]. During this procedure, a catheter is inserted into the urethra and advanced through the striated urinary sphincter and bladder neck into the bladder to drain urine. If there is a need for continuous urine drainage, a balloon surrounding the tip of the catheter is inflated in the bladder to hold the catheter proximal to the bladder neck[2]. UC is performed for therapeutic purposes, such as urinary retention[3], bladder emptying or urine output measurement perioperatively or in acutely ill patients[3,4], urinary incontinence, drug delivery, bladder irrigation[5], social and hygiene reasons[3], or diagnostic purposes, such as urodynamic studies, cystogram imaging, collection of urine sample[5]. Apart from clean intermittent self-catheterization, UC is a sterile procedure that requires special knowledge to assess the indications, contraindications, and potential complications, and specific technical skills to expose the urethral meatus properly and align the urethra correctly during the maneuver[6].

Successful UC is operator dependent. Early detection of risk factors for complications in males, such as enlarged prostate and urethral strictures, allows for correct decision-making to prevent UC-related morbidity[7]. Similarly in women, special skills and maneuvers are required when risk factors for difficult catheterization are present, such as morbid obesity, intravaginal retraction of the urethral meatus, and prolapse, to optimize the visualization and access the introitus and urethral meatus[8,9]. Iatrogenic urethral trauma caused by medical intervention occurs in 0.3% of catheterization maneuvers during the insertion process, and is mainly caused by false passage creation or intraurethral balloon inflation and may lead to short-term and long-term morbidities[10,11]. Complications from UC increase hospital stay

by 9.4±10 days and costs by €335,377 over 6 months[10]. Recent efforts are focusing on the identification of strategies to reduce inappropriate catheter use and avoid associated complications in the short-term, such as infection, hematuria, obstructive kidney injury, need for transfusion or cystoscopy, and long-term, such as urethral stricture, need for long-term bladder catheter or self-urethral dilation[12–16].

At the university hospital, UC is performed traditionally by physicians. Newly qualified doctors are expected to safely and confidently perform UC at the end of their training[1]. However, recent literature shows that junior doctors receive inadequate theoretical and practical training concerning UC during their years in medical school[7,17] before their direct involvement in patient care. The rate of medical interns who did not feel prepared to perform this skill varied in the literature between 18.6% and 55.6% among the Irish[7], Scottish[18], French[19], American[20], and Filipino[17] experiences. Most catheterization-related morbidities occur when interns perform UC, especially at the beginning of their internship and when they are unsupervised[1,7]. Gaps were reported in the theoretical knowledge of the steps of UC, and the mechanisms and identification of iatrogenic injuries[17]. On the practical side, nearly half of new interns have not undergone a urology rotation during their undergraduate training[21,22] and have never performed UC. The majority reported a lack of previously supervised catheterization and especially in female patients[21].

UC training programs successfully improved the knowledge of new interns and their confidence level in performing the skill[23,24] and reduced the incidence of iatrogenic injuries. Still, the real benefit of simulation-based training in UC is debatable because the current models may lack the typical challenges encountered in actual patients, such as increased pain, difficult entry to the bladder, and anatomical variations.

Therefore, as part of a plan to reduce catheter-related morbidity in practice, this study was conducted to evaluate new interns' declarative knowledge regarding UC and assess the effectiveness of a urology simulation session in improving their declarative knowledge score, perceived self-efficacy (PSE), and technical skills.

Methods

This study employed a prospective single-group pre–post educational design to evaluate the impact of a simulation-based urethral catheterization workshop on interns' knowledge, technical performance, and perceived self-efficacy.

~~This study was reviewed and approved by the University Institutional Review Board, with an approval date from June 2022 to July 2023, during which data was collected and simulation sessions were done. Participation was voluntary, and all interns provided written informed consent before inclusion. All procedures conformed to the principles of the Declaration of Helsinki.~~

Participant recruitment

The urology simulation session was conducted for new interns at the University Hospital. This was done at the beginning of their academic year in order to adequately train the interns on proper, safe, and comfortable technique. Inclusion criteria for the study mandated that all participants were first-year interns who graduated from the affiliated medical school the previous year. Furthermore, all participants were not previously part of a urology training or simulation session and had no extra specialized urology-related training aside from their rotations during undergraduate training. All participants had the study thoroughly explained to them and were asked to sign an informed consent prior to enrollment.

Pre-testing

The urology simulation session was a one-day curriculum organized by the urology department to teach participants about UC. Interns were divided into small groups of eight. The simulation session had 2 theoretical and one practical module of twenty minutes each. Prior to starting the educational modules, all participants were asked to fill out an anonymous, online questionnaire and perform a foley catheter insertion on the available male and female simulation models.

The first section in the questionnaire asked about demographic characteristics such as age, sex, self-reported confidence level on a scale of 1 to 10, previous attempts at inserting a foley catheter, and opinion on preferred method of training for hands-on procedures. The following section included 2 open ended questions asking participants to give the indications and contraindications for UC, and 13 dichotomous or multiple-choice questions with a single correct answer that were inspired by previous similar works. This section assessed medical knowledge related to catheter insertion, such as the types of available catheters and sizes, optimal place to apply lubricant, angle of penis during insertion, depth of catheter insertion, and importance of drained urine volume after insertion. Self-reported confidence in performing urethral catheterization was measured using a numerical rating scale from 1 to 10, where 10 indicated the highest level of confidence. Perceived self-efficacy was evaluated using a 5-point Likert scale reflecting the participant's perceived preparedness to perform urethral catheterization. These items were developed for the purpose of this educational evaluation and are consistent with approaches commonly used in simulation-based medical education studies to capture changes in perceived competence following training. Finally, the perceived self-efficiency (PSE) score reflected interns' beliefs in their competence to perform UC, and involved a self-reflective 5-point Likert scale, for the question "How prepared do you feel to

perform UC on a scale from 1 to 5 (where 5 is the highest and 1 is the lowest preparedness levels)?". The questionnaire was reviewed by the teaching urologist involved in the training program to ensure clarity and relevance to the learning objectives.

The knowledge questionnaire included two open-ended questions assessing recognition of indications and contraindications for urethral catheterization, as well as thirteen multiple-choice or dichotomous questions addressing key procedural and theoretical aspects of catheterization, including catheter types and sizes, patient and operator positioning, sterile technique, exposure of the urethral meatus, lubrication, insertion depth, balloon inflation, and procedural documentation. Each correct response was assigned one point, generating a cumulative declarative knowledge score used for pre- and post-training comparison. The questionnaire items were developed based on previously published educational studies on urethral catheterization and standard urology references, including Campbell-Walsh Urology, to ensure that the content reflected clinically relevant knowledge and procedural steps expected from new medical interns.The first attempt of foley insertion was done under supervision of a senior resident, who utilized the Objective Structured Assessment of Technical Skill (OSATS)[25] rubric to assess the skills and proficiency of each participant. Each participant performed a catheterization attempt before the educational session and again after completion of the training. The procedures were evaluated by senior urology residents supervising the simulation session, who were familiar with the OSATS evaluation tool. Each step in the rubric was assigned one point, for a maximum score of 15. Pre- and post-training OSATS scores were summarized using mean±standard deviation and compared using paired statistical testing to assess improvement in technical performance following the training.

The assessed skills included history taking for indications and contraindications, correct choice of balloon inflation solution, catheter type and material, proper patient and operator

positioning, adequate sterilization of the genitals and maintenance of sterile technique during insertion, optimal exposure of urethral meatus, application of lube, penile stretching and angling, catheter insertion, balloon inflation, and urine drainage (Table 1).

Table 1. OSATS rubric for trainee evaluation concerning urethral catheterization

Skill performance checkpoints	Points
Indication (history) check	1
Contraindications check	1
Choice of catheter type	1
Choice of catheter size	1
Patient positioning	1
Operator positioning	1
Genital cleansing	1
Balloon integrity check	1
Exposure of urethral meatus	1
Urethra alignment	1
Lubricant application	1
Depth of insertion of the catheter	1
Choice of balloon inflation material	1
Verification of the drained urine volume	1
Documentation	1

OSATS=Objective Structured Assessment of Technical Skills. Each checkpoint was assigned 1 point, for a maximum total score of 15.

Training development

The training curriculum was developed based on previous works in literature[7,17]. All teaching sessions were given by an experienced urologist with more than five years of teaching experience. The learning objectives of the simulation session were to educate the participants on the indications, contraindications, and the steps of a correct UC, while also enhancing the technical skills and confidence in their performance of UC.

After the pretest, the first theoretical module involved a short, interactive PowerPoint presentation which highlighted key points in UC: anatomical considerations in males and females, indications, contraindications, catheter types and selection criteria, detailed steps of UC in males and females, complications, and the importance of documentation and seeking

help for complex cases. There was also emphasis on the importance of the "one gentle attempt only" rule to reduce the likelihood of iatrogenic false passage[10]. Materials were selected from the Campbell-Walsh Urology, 11th edition[23]. In the second theoretical module, catheter insertion was demonstrated by videos showing the steps of UC with verbal descriptions. Videos were specific to the female and male dummies available at the simulation center, and were picked from the provider's website[26,27]. Lastly, interns reattempted UC on male and female dummies under the supervision of the senior urology residents, who commented on the technique and outlined the flaws. The senior residents again utilized the OSATS rubric to assess improvement of technical skills.

The workshop was conducted in small groups of approximately eight participants to facilitate interaction and individualized instruction. Each group was supervised by a senior urology resident, resulting in an approximate instructor-to-learner ratio of 1:8 during the simulation exercises. The hands-on simulation module lasted approximately 20 minutes, during which participants practiced urethral catheterization on male and female simulation models. Trainees were encouraged to perform the procedure while verbalizing each step. Feedback was provided immediately after each attempt using a structured, formative approach, in which the supervising resident highlighted correct steps, identified technical errors, and provided practical suggestions for improvement. This immediate feedback aimed to reinforce correct technique and address common procedural mistakes.

Transfer of learning

The educational impact of the simulation was assessed following Kirkpatrick’s four-level model. Levels 1 and 2 (reaction and learning) were evaluated through participant

satisfaction, knowledge, confidence, and perceived self-efficacy immediately after the session. Levels 3 and 4 (behavioral change and results) were assessed at six-month follow-up through clinical performance indicators, number of catheterizations performed, procedural success rate, and complication rate, providing evidence of transfer of learning into clinical practice[28].

Six months after the simulation, a remote assessment was conducted to evaluate behavioral change (Level 3) and patient-related outcomes (Level 4). Data on the number, success, and complications of catheterizations were collected from interns' clinical logbooks and verified by supervising urology residents. This approach allowed pragmatic evaluation of how simulation-based training translated into real clinical performance.

Procedural success was defined as successful insertion of the urinary catheter with appropriate urine drainage and no need for additional attempts by another operator or alternative catheterization techniques. Complications were defined as any adverse event occurring during or following the catheterization procedure, including urethrorrhagia, false passage creation, urinary tract infection, or the need for suprapubic catheterization. Follow-up data were collected from interns' procedural logbooks during their clinical rotations and were reviewed in collaboration with supervising urology residents and physicians to verify the reported number of procedures, procedural outcomes, and complications.

Statistical analysis

Interns' declarative knowledge and PSE scores were reported using descriptive statistics with means and standard deviations or frequencies and percentages. Pre- and post-training scores were compared using paired t-tests. Although perceived self-efficacy and

confidence were measured using Likert-type scales, these outcomes were treated as approximately continuous variables and summarized using means and standard deviations, a commonly accepted approach in educational research for within-subject comparisons. Effect sizes with corresponding 95% confidence intervals were also reported to facilitate interpretation of the magnitude of observed changes. Statistical significance was defined as $p < 0.05$.

Data from surveys were collected using an online form. The analysis was conducted using RStudio 2021.09.0+351 "Ghost Orchid" Release[29]. The inclusion of six-month outcome data permitted assessment of behavioral change and patient-level results (Kirkpatrick Levels 3 and 4), expanding the evaluation beyond knowledge and self-efficacy gains.

Ethical considerations

This study was reviewed and approved by the Ethical Review Board of the Holy Spirit University of Kaslik (USEK), Jounieh, Lebanon (Approval number: EN-CETH-001). All participants were informed about the objectives of the study prior to enrollment. Written informed consent was obtained from all participants before participation. The study procedures were conducted in accordance with the ethical principles outlined in the Declaration of Helsinki.

Results

Participant demographics

The study initially recruited 44 interns, however, 4 were not included as they had previously participated in general simulation sessions that included foley catheterization during their undergraduate training.

40 new medical interns participated in the urology simulation session, of which 35% (n=14) were males and 65% (n=26) were females. All had completed five years of studies at the same medical school. At the time of the training, only 15% (n=6) felt prepared correctly (Table 2).

Table 2. Study participants' demographic characteristics

Variable	Characteristics	N (%)
Age (years)	25-26	40 (100%
Sex	Males<br>Females	14 (35%)<br>26 (65%)
Consent to participate	Yes<br>No	40 (100%)<br>0 (0%)

~~UC=urethral catheterization.~~

Percentages are calculated based on the total number of participants (N = 40).

Participant initial knowledge

All participants considered history taking and contraindications recognition essential before UC and were able to identify at least 3 indications, such as urinary drainage during retention, control of incontinence, and bladder irrigation, and contraindications, such as urethral trauma, acute infection, and known bladder tumors. The majority (92%) did not know the difference between catheter types and sizes. Only 38% (n=15) would position the patient supine for catheterization, and 43% (n=17) would stand at the correct patient side, which is the side of the operator's dominant hand. All of them agreed that the procedure should be sterile. Most participants did not know how to correctly expose the urethral meatus (75%, n=30), the importance of the solution used to inflate the balloon (100%, n=40), or that

lubricant must be applied into the urethra (25%, n=10). In contrast, most interns (83%, n=33) knew that the penis should be stretched perpendicular to the body during catheterization, and that the catheter had to be introduced to the hub (65%, n=26). The majority agreed on the importance of documentation (85%, n=34) and assessment of the volume drained (90%, n=36) (Table 3 and further detailed in Supp Table 1~~3~~).

Table 3. Pre- and post-test interns' declarative knowledge concerning urethral catheterization

**Questions**	**Pre-training correct n(%)**	**post-training correct n(%)**	**McNemar p-value**
**What are the indications for UC?**	40 (100)	40 (100)	-
**What are the contraindications for UC?**	40 (100)	40 (100)	-
**Do you know the difference between catheter types?**	3 (8)	34 (85)	**<0.001**
**Are you able to correctly choose the catheter size?**	4 (8)	31 (77.5)	**<0.001**
**What is the best patient position during UC?**	15 (38)	40 (100)	**<0.001**
**Where is the best position of the operator who is performing the UC?**	17 (43)	17 (42.5)	-
**Should the UC be a sterile procedure?**	40 (100)	40 (100)	-
**Should the balloon be checked before UC?**	1 (3)	25 (62.5)	**<0.001**
**Do you know how the correctly expose the urethral meatus in males and females?**	10 (25)	10 (25)	-
**At which angle should the penis be stretched during UC?**	33 (83)	33 (83)	-
**Where is the lubricant best applied before UC?**	25 (63)	33 (82.5)	**<0.001**
**What is the level or depth of UC insertion?**	26 (65)	35 (87.5)	**0.004**
**Is the type of solution used to inflate the balloon important?**	0 (0)	37 (92.5)	**<0.001**
**Is the volume of urine drained important?**	36 (90)	36 (90)	-
**Is procedure documentation important?**	34 (85)	37 (92.5)	0.453

Values are presented as n (%)

~~**Table 3. Changes in the OSATS scores for TS and NTS before and after training.**~~

Formatted Table

Formatted: English (United States)

Formatted: Font: Bold

Formatted: Font: Bold

Formatted: Font: Bold

Formatted: Font: Bold

Formatted: Font: Bold

Formatted: Font: Bold

Formatted: Font: Bold

	Before training	After training	P-value
TS scores	8.9±2.1	12.8±2.3	<0.01
NTS scores	6.8±2.2	7.2±3.1	0.4

TS=technical skills; NTS=non-technical skills; OSATS=Objective Structured Assessment of Technical Skills.
Values are presented as mean±standard deviation.
P-values were calculated using paired statistical testing comparing scores before and after training.

Variation in knowledge, PSE, and confidence levels

The prepre- and post-training assessments showed an increase in the mean declarative knowledge score from 7.7±1.24 to 12.2±1.11, and in the mean PSE score, which increased from 3.32±0.94 to 4.32±0.62. Prior to training, the self-reported confidence levels (SRCL) ranged from 1 to 10, with a median of 7 and a mean of 6.6±1.79. After the training session, the levels varied exclusively between 7 and 10. The mean confidence level was 8.66±0.94, with a median of 9. All the improvements showed a statistical significance (p<0.001) (Table 4).

Table 4. Changes in the OSATS scores for TS and NTS before and after training.

	Before training	After training	P-value
TS scores	8.9±2.1	12.8±2.3	<0.01
NTS scores	6.8±2.2	7.2±3.1	0.4

TS=technical skills; NTS=non-technical skills; OSATS=Objective Structured Assessment of Technical Skills.
Values are presented as mean±standard deviation.
P-values were calculated using paired statistical testing comparing scores before and after training.

Formatted: Indent: First line: 0"

Table 4. Pre-training interns' declarative knowledge concerning urethral catheterization

Questions	Answers	n	%
What are the indications for UC?	Given 3 correct answers	40	100%
	Less than 3 correct answers	0	0
What are the contraindications for UC?	Given 3 correct answers	40	100%
	Less than 3 correct answers	0	0
Do you know the difference between catheter types?	Yes	3	8%
	No	37	92%
Are you able to correctly choose the catheter size?	Yes	3	8%
	No	37	92%
What is the best patient position during UC?	Supine	15	38%
	Semi-seated	25	62%
Where is the best position of the operator who is performing the UC?	The patient side which is the same as the operator's dominant hand	17	43%
	The patient side which is opposite to the operator's dominant hand	23	57%
Should the UC be a sterile procedure?	Yes	40	100%
	No	0	0%
Should the balloon be checked before UC?	Yes	1	3%
	No	39	97%
Do you know how the correctly expose the urethral meatus in males and females?	Yes	10	25%
	No	30	75%
At which angle should the penis be stretched during UC?	Perpendicular to the body	33	83%
	Parallel to the body	7	17%
	No particular angle	0	0%
Where is the lubricant best applied before UC?	Catheter	10	25%
	Urethra	25	63%
	Urethral meatus	5	12%
What is the level or depth of UC insertion?	To the hub (where the connection for a drainage tube and the inflation port meet)	26	65%
	Past the mid-point of the shaft of the catheter	5	12%
	No particular level: the balloon is inflated as soon as the urine passes from the catheter	9	23%
Is the type of solution used to inflate the balloon important?	Yes	0	0%
	No	40	100%
Is the volume of urine drained important?	Yes	36	90%
	No	4	10%
Is procedure documentation important?	Yes	34	85%
	No	6	15%

PSE = perceived self-efficacy.
Values are presented as mean ± standard deviation.
Mean differences are reported with 95% confidence intervals.
P-values were calculated using paired statistical testing comparing scores before and after training.

Formatted Table

Participant satisfaction

Prior to the training, participants were asked to self-report their readiness to perform UC in the hospital, resulting in 34 (85%) feeling unprepared to perform the procedure. The most common teaching modalities among the new interns were live simulation (n=20, 53%) followed by supervised bedside teaching (n=18, 47%) (Table 5a). At the end of the training, the survey showed that 92.5% (n=37) of the trainees were very satisfied. All (n=40) rated the quality of the training as good and 97.5% (n=39) reported that the training met their needs and that the skills acquired were useful in their daily work. Almost all trainees (97.5%, n=39) stated that they would recommend this training to others The magnitude of the improvements is reflected by the mean differences and their 95% confidence intervals, indicating substantial gains in knowledge, perceived self-efficacy, and confidence following the training session (Table 5b).

Table 5a. Pre-test participant preparedness and preferred method of learning

Feeling adequately prepared for UC*	Yes	6 (15%)
	No	34 (85%)
Preferred method of training	Live simulation demonstration	20 (53%)
	Supervised bedside catheterization	18 (47%)
	Video	0 (0%)
	Conference	0 (0%)

UC=Urethral catheterization
Percentages are calculated based on the total number of participants (N = 40).

Formatted: Font: Bold

Formatted: Font: Bold

Table 5b. Changes in the mean PSE, self-reported confidence level, and declarative knowledge scores before and after training.

	Before training	After training	Mean of difference [95% confidence interval]	P-value
Mean declarative knowledge score	7.7±1.24	12.2±1.11	4.5 [4.07-4.92]	<0.05
Mean PSE score	3.32±0.94	4.32±0.62	1 [0.65-1.36]	<0.05

Self-reported confidence level	6.60±1.79	8.66±0.94	2.05 [1.39-2.72]	<0.05

PSE=perceived self-efficacy.
Values are presented as mean ± standard deviation.
Mean differences (effect size) are reported with 95% confidence intervals.
P-values were calculated using paired statistical testing comparing scores before and after training.

Transfer of learning outcomes

Over 6 months, interns collectively performed 274 urinary catheterizations, reflecting sustained application of learned skills in clinical settings (Level 3). The median number of procedures performed per intern was 5 [IQR: 4–10], demonstrating variability in individual procedural exposure. The overall procedural success rate was 93%, with a low complication rate of 4% (Level 4), indicating that simulation training positively influenced both behavior and patient outcomes. The most common causes of failure were urethral meatal stenosis in 32% (n=6) and difficulty in identifying the urethral meatus in women in 32% (n=6). Other reported causes were inadequate exposure of the genitals due to patient disability (e.g., hip fracture) in 26% (n=5) and phimosis in male patients in 10% (n=2). Overall, 4% (n=11) were complicated (Table 6).

Formatted: Font: 12 pt

Table 6: participant satisfaction towards the boot camp

Domain	Answer	n	%
Satisfaction	Very well	37	92.5%
	Ok	3	7.5%
Quality of training	Very well	40	100%
	Ok	0	0%
Meeting the needs of trainees	Very well	39	97.5%
	Ok	1	2.5%
Usefulness of the skills acquired in daily practice	Yes	39	97.5%
	No	1	2.5%
Recommendation of the exercise to other trainees	Yes	39	97.5%

Formatted Table

No	1	2.5%

Percentages are calculated based on the total number of participants (N=40). Responses were collected through a post-training satisfaction questionnaire.

The most common complication was urethrorrhagia with spontaneous resolution in 55% (n = 6) of cases. This was followed by the occurrence of a false passage in 18% (n=2), a urinary tract infection in 18% (n=2) and the need for suprapubic catheterization in 9% (n=1) (Table 7).

Table 7. Rate and causes of urinary catheterization failure and complications.

Formatted Table

**Description**	**n**	**%**
**Total UC performed**	274	100
**Failed attempts**	**19**	**7%**
**Reasons for failed attempts (n=19)**	-	-
**Difficulty identifying urethral meatus (Female)**	6	32%
**Phimosis (Male)**	2	10%
**Inadequate exposure of the genitals**	5	26%
**Stenosis of the urethral meatus**	6	32%
**Complications**	**11**	**4%**
**Urethrorrhagia with spontaneous resolution**	6	55%
**False passage creation**	2	18%
**Urinary tract infection**	2	18%
**Need for suprapubic catheterization**	1	9%

UC=urethral catheterization.
% for procedure outcomes are based on total catheterisation attempts (n=274)
% for reasons for failed attempts are based on total failed attempts (n=19)
% for complications are based on total complications (n=11)

~~**Table 7. Rate and causes of urinary catheterization failure and complications.**~~

~~**Description**~~	~~**n**~~	~~**%**~~
~~**Total UC**~~	~~274~~	~~100~~
~~**Failed attempts**~~	~~**19**~~	~~**7%**~~
~~**Difficulty identifying urethral meatus (Female)**~~	~~6~~	~~32%~~
~~**Phimosis (Male)**~~	~~2~~	~~10%~~
~~**Inadequate exposure of the genitals**~~	~~5~~	~~26%~~
~~**Stenosis of the urethral meatus**~~	~~6~~	~~32%~~
~~**Complications**~~	~~**11**~~	~~**4%**~~
~~**Urethrorrhagia with spontaneous resolution**~~	~~6~~	~~55%~~
~~**False passage creation**~~	~~2~~	~~18%~~
~~**Urinary tract infection**~~	~~2~~	~~18%~~
~~**Need for suprapubic catheterization**~~	~~1~~	~~9%~~

~~**UC=urethral catheterization.**~~
~~**Percentages for causes of failure and complications are calculated relative to the total number of failed attempts (n=19) and total complications (n=11), respectively.**~~

Discussion

The primary purpose of the simulation session was to bridge the gap between the knowledge gained in medical school and the technical skills needed in clinical practice. Based on the previous literature showing that new interns caused higher UC-related morbidity[1], this training was organized at the beginning of the internship with the aim of teaching new interns the essential steps and basic clinical skills for UC and allowing them to practice on dummies before catheterizing real patients, which may help support the development of confidence and competence in performing UC[17].

The self-assessment questionnaire ~~conducted~~ administered before the training helped the trainees identify their weaknesses, strengths, knowledge, and PSE regarding UC. Only 15 % of the new interns felt adequately prepared to perform UC. The rate was lower than what was reported in the literature by Manalo et al. (55.6%)[17], Thomas et al. (36% and 52%)[7], and Bigot et al. (26% and 38.3%)[19], which raises the need to focus on procedural skill teaching in medical school and emphasizes the importance of the simulation session organized. All our interns (100%) were aware of the importance of history-taking before proceeding to catheterization, in contrast to 23.6%[17] and 26.7%[30] reported in previous studies by Manalo et al. and Browne et al, respectively. This step is of great importance because it allows the recognition of risk factors of complications[17]. More than half of the participants (63%) knew adequately that the lubricant should be applied inside the urethra, despite the tendency to apply it to the catheter, as reported in 97.8% of cases in the Manalo et al. study[17]. Most of our interns were aware of the importance of documentation (85%) and assessment of the volume drained (90%), in contrast with the low rates of

documentation (only in 29% of patients with an indwelling catheter) reported by Cornia et al.[31]

The training focused on the weaknesses identified by the initial survey regarding catheter types and sizes, patient positioning during catheterization, operator positioning at the bedside, correct exposure of the urethral meatus, and the importance of the solution used to inflate the balloon. The short learning modules of 20 minutes each was efficient to increase knowledge retention while maintaining attention and focus. The division of interns into small groups of 8 individuals helped optimize the learning experience without excessive crowding and time restrictions. Also, the incorporation of different methods in the training, such as a didactic lecture, video-based demonstration, and hands-on simulation, maximized the teaching experience, as demonstrated by previous works[32,33]. Despite being underreported in various urology curricula, simulation-based learning has demonstrated its efficacy in urology training[32,34]. Participation in the simulation session was associated with an increase in declarative knowledge and PSE scores among the new interns concerning UC. Similarly, Waters et al. demonstrated the beneficial effects of interactive teaching of UC on the confidence level and examination scores[32]. Browne et al. also reported a beneficial effect of the structured practical training on the SRCL of new interns on a different categorical scale[30].

The inclusion of a six-month follow-up assessment extends this study beyond Kirkpatrick Level 2, demonstrating measurable behavioral change and patient-related outcomes. The high procedural success and low complication rates observed during follow-up provide descriptive insight into interns' early clinical experience with catheterization. However, these observational data should be interpreted cautiously, as ongoing clinical

exposure, supervision, and repeated procedural practice during the internship period may also have influenced these outcomes.

The originality of this study remains in its timing, clear methodology, and objective outcome assessment. The academic timing of the simulation session just before the beginning of the internship both boosted learning capabilities for the participants while also reducing bias in participant experience. At the time of the simulation session, interns would have completed medical school and acquired sufficient medical background to understand the anatomical and physiological basis needed for UC, however, as shown in the results, few of them have ever performed UC on patients. The timing proposed in this study differed from the previous work done by Waters et al., where teaching targeted second-year medical students[32]. To facilitate the assimilation of the UC, there was focus on the essential steps using the standard two-way catheters, without much complicated discussion of the other specific aspects of UC, such as three-ways catheterization, bladder washout, and continuous bladder irrigation, like Browne et al.[30] because those topics were considered more specific to urology residents. The impact of the training session was assessed using an objective declarative knowledge score that was calculated before and after the training, without exclusive reliance on the self-reported outcome such as the perceived-self efficacy and SRCL in this study as in previous works in the literature[30].

This study had several limitations. First, The study did not include a parallel control group, as all new interns at the university were required to attend the simulation session as part of their induction curriculum. Although the pre-test served as a within-subject baseline, future studies should adopt a controlled or randomized design comparing simulation-based teaching with traditional instruction to better delineate causal effects. Second, the effect of

the training on UC success and complications in clinical practice on real patients was not observed by the evaluators in the simulation session. Instead, at this stage, there was more reliance on self-reported parameters and knowledge. Third, the small population size, while representative of the population of medical graduates working as new interns, may be a limiting factor for larger programs that incorporate hundreds of new interns every year, emphasizing the importance of this study to be redone in different settings such as university hospitals with larger intern bodies. Also, given the single-group pre–post design of this study, the observed improvements in knowledge, confidence, and clinical performance cannot be attributed exclusively to the simulation session. During the six-month follow-up period, interns were also exposed to routine clinical practice, supervision from senior residents and attending physicians, and repeated procedural experience, which may have contributed to the observed outcomes. Therefore, the results should be interpreted as an association between participation in the training program and improved outcomes rather than a definitive causal relationship. Finally, the study method used simulation with peer feedback, which is considered superior to other conventional learning styles such as lectures, videos, and demonstrations. However, metric-dependent proficiency-based progression training is recently considered the most efficient and performant way to optimize the learning experience[32]. As such, this study may further benefit as a steppingstone to assess the use of metric-dependent proficiency-based progression in different surgical specialties and especially in urology.

Conclusion

Junior doctors have insufficient knowledge regarding UC. Participation in the urology simulation session was associated with improvements in declarative knowledge scores, SRCL, and perceived self-efficacy regarding UC among new interns, showing promise in improving technical skills and confidence for newly graduated interns being introduced into medical practice. Future studies using controlled or randomized designs would be valuable to better isolate the specific impact of simulation-based training on clinical outcomes.

Lessons for Practice

- Simulation-based training for urethral catheterization may help bridge the gap between theoretical knowledge and clinical performance among new medical interns.
- Early, structured procedural training during internship orientation may contribute to improvements in interns' confidence, technical proficiency, and patient safety outcomes.
- Incorporating simulation sessions into intern induction programs supports sustainable improvements in procedural success rates and reductions in catheter-related complications.

References

1. Bhatt NR DN Quinlan MR, et al. A prospective audit on the effect of training and educational workshops on the incidence of urethral catheterization injuries. *Can Urol Assoc J*. 2017;11(7):E302-E306. doi:10.5489/cuaj.4271

2. Tan E AA Kankam H, Menezes P. Urinary catheterization 2: technique and managing failure. *Br J Hosp Med*. 2019;80(9):C136-C138. doi:10.12968/hmed.2019.80.9.C136

3. Tan E AA Kankam H, Menezes P. Urinary catheterization 1: indications. *Br J Hosp Med*. 2019;80(9):C133-C135. doi:10.12968/hmed.2019.80.9.C133

4. Clayton JL. Indwelling Urinary Catheters: A Pathway to Health Care–Associated Infections. *AORN Journal*. 2017;105(5):446-452. doi:10.1016/j.aorn.2017.02.013

5. Wilson M. Causes and management of indwelling urinary catheter-related pain. *Br J Nurs*. 2008;17(4):232-239. doi:10.12968/bjon.2008.17.4.28712

6. Haider MZ AP. Bladder Catheterization. In: StatPearls. *StatPearls Publishing*. Published online 2022.

7. Thomas AZ GS Meagher D, Creagh T. Avoidable iatrogenic complications of urethral catheterization and inadequate intern training in a tertiary-care teaching hospital. *BJU Int*. 2009;104(8):1109-1112. doi:10.1111/j.1464-410X.2009.08494.x

8. Ghaffary C YA Villanueva C, Leslie SW. A Practical Approach to Difficult Urinary Catheterizations. *Curr Urol Rep*. 2013;14(6):565-579. doi:10.1007/s11934-013-0364-3

9. Willette P CS. Current Trends in the Management of Difficult Urinary Catherizations. *WestJEM*. 2012;13(6):472-478. doi:10.5811/westjem.2011.11.6810

10. Davis NF QM Bhatt NR, et al. Incidence, Cost, Complications and Clinical Outcomes of Iatrogenic Urethral Catheterization Injuries: A Prospective Multi-Institutional Study. *J Urol*. 2016;196(5):1473-1477. doi:10.1016/j.juro.2016.05.114

11. Croghan SM HL O'Connor EM, et al. A Prospective Multi-Institutional Evaluation of Iatrogenic Urethral Catheterization Injuries. *Journal of Investigative Surgery*. 2022;35(10):1761-1766. doi:10.1080/08941939.2022.2109226

12. Knoll BM WD Ellingson L, et al. Reduction of Inappropriate Urinary Catheter Use at a Veterans Affairs Hospital Through a Multifaceted Quality Improvement Project. *Clinical Infectious Diseases*. 2011;52(11):1283-1290. doi:10.1093/cid/cir188

13. Fakih MG. Reducing Inappropriate Urinary Catheter Use: A Statewide Effort. *Arch Intern Med*. 2012;172(3):255. doi:10.1001/archinternmed.2011.627

14. Niederhauser A ZS Marschall J, Schwappach DL. Nurses' and Physicians' Perceptions of Indwelling Urinary Catheter Practices and Culture in Their Institutions. *J Patient Saf*. 2020;16(2):e82-e89. doi:10.1097/PTS.0000000000000502

15. Meddings J RM Krein SL, Fakih MG, Olmsted RN, Saint S. Reducing unnecessary urinary catheter use and other strategies to prevent catheter-associated urinary tract infection: an integrative review. *BMJ Qual Saf*. 2014;23(4):277-289. doi:10.1136/bmjqs-2012-001774

16. Murphy C FM Prieto J. Interventions to minimise the initial use of indwelling urinary catheters in acute care: A systematic review. *International Journal of Nursing Studies*. 2014;51(1):4-13. doi:10.1016/j.ijnurstu.2012.12.007

17. Manalo M LM Buckley BS. Medical interns' knowledge and training regarding urethral catheter insertion and insertion-related urethral injury in male patients. *BMC Med Educ*. 2011;11(1):73. doi:10.1186/1472-6920-11-73

18. Grimes N LJ McKay A, McIlhenny C. Foundation Year 1 doctors' experience and confidence in catheterisation: A multicentre survey. *Journal of Clinical Urology*. 2019;12(5):380-384. doi:10.1177/2051415819854924

19. Bigot P RM Orsat M, et al. Évaluation des compétences pratiques en fin de deuxième cycle des études médicales : exemple du drainage du bas appareil urinaire. *Progrès en Urologie*. 2008;18(2):125-131. doi:10.1016/j.purol.2007.10.003

20. Vigran H, Krischak M, Khoei A, Wan J, Romo PB, Hadj-Moussa M. Efficacy of a Targeted Foley Catheter Education Program. *Journal of Surgical Education*. 2025;82(9):103578. doi:10.1016/j.jsurg.2025.103578

21. Cetti R SR Bissell L, Shaw R. The urological foot soldier: are we equipping our foundation-year doctors? *The Bulletin of the Royal College of Surgeons of England*. 2010;92(8):284-287. doi:10.1308/147363510X12779829582415

22. Nic An Ríogh AU OC Lonergan PE, Davis NF. Designing and assessing a urethral catheter skills workshop for intern doctors in a university teaching hospital. *Ir J Med Sci*. 2020;189(4):1501-1506. doi:10.1007/s11845-020-02240-4

23. Tailly T DJD. Fundamentals of Urinary Tract Drainage. In: *Campbell-Walsh Urology.* 11th ed. Elsevier; 2016:119-124.

24. Mubarak M IQ Alsaeed M, Alalawi M. Transurethral Catheterization in Early Training: The Impact of Peer-Led Mentorship. *Surgery Research and Practice*. 2021;2021:1-6. doi:10.1155/2021/8498835

25. Martin JA, Regehr G, Reznick R, et al. Objective structured assessment of technical skill (OSATS) for surgical residents. *Br J Surg*. 1997;84(2):273-278. doi:10.1046/j.1365-2168.1997.02502.x

26. Limbs LT. Female Catheterization Trainer. *Limbs & Things*. Published online 2021.

27. Limbs LT. Male Catheterization Trainer. *Limbs & Things*. Published online 2021.

28. Kirkpatrick D. Techniques for evaluation training programs. Published online 1959.

29. R Core Team. R : A Language and Environment for Statistical Computing. *R Foundation*. Published online 2021.

30. Browne C NS Nolan JM, et al. The impact of a structured clinical training course on interns' self-reported confidence with core clinical urology skills. *Ir J Med Sci*. 2018;187(1):255-260. doi:10.1007/s11845-017-1616-x

31. Cornia PB AJ Fraser S, Saint S, Lipsky BA. Computer-based order entry decreases duration of indwelling urinary catheterization in hospitalized patients. *Am J Med*. 2003;114(5):404-407. doi:10.1016/s0002-9343(02)01568-1

32. Waters PS MT Kelly BD, et al. The acquisition and retention of urinary catheterisation skills using surgical simulator devices: teaching method or student traits. *BMC Med Educ*. 2014;14:264. doi:10.1186/s12909-014-0264-3

33. Lehmann R SA Bosse HM, Lutz T, Huwendiek S. Student perceptions of a video-based blended learning approach for improving pediatric physical examination skills. *Ann Anat*. Published online 2016.

34. Ballouhey Q CJ Lescure V, Clermidi P, Romain J, Guigonis V. Simulation et rétention des acquisitions : application au drainage vésical. *Prog Urol*. 2015;25(9):516-522.

Response to Reviewer 1

We sincerely thank the reviewer for the thoughtful and constructive comments. We greatly appreciate the time and effort dedicated to reviewing our manuscript. The suggestions have significantly improved the clarity, organization, and scientific rigor of the paper. All comments were carefully considered and addressed as detailed below.

Comment 1

The term “intern” is used throughout the manuscript; however, this may be ambiguous, as it can refer to different healthcare professions (e.g., nursing, pharmacy, or medicine). For clarity and precision, I recommend specifying “medical intern” in the title, and in the content for the first mention.

Response: The terminology has been revised throughout the manuscript to specify “medical intern” where appropriate, including in the title and at first mention within the text, in order to improve clarity and avoid ambiguity regarding the study population.

Comment 2

Some statements need revision, as detailed in the document. For example, the phrase “… questionnaire conducted ….” should be revised to “… questionnaire administered ….”

Response: The manuscript has undergone thorough linguistic revision, and the suggested wording changes, including replacing “questionnaire conducted” with “questionnaire administered,” have been implemented throughout the manuscript to improve readability and academic style.

Comment 3

It is indicated that training was provide to the new interns, however, it is not specified at what point of internship. This is only mentioned in the discussion, please consider clarifying when (specific time) the medical interns were recruited and trained in the methods section.

Response: The Methods section has been revised to clarify the timing of recruitment and training. Specifically, we now state that the simulation workshop was conducted at the beginning of the interns’ academic year, prior to substantial clinical exposure, as part of their early internship orientation and training.

Comment 4

The ethics statement (in the Methods introduction) indicates approval by the “University Institutional Review Board,” however the specific university/institution is not identified. For clarity and transparency, please specify the name of the university (and include the approval reference number if available). Also, the ethical statements are repeated in both the Methods introduction and Ethical considerations sections. These can be consolidated under Ethics consideration section:

Response: The ethical approval statement has been clarified and consolidated within the dedicated “Ethical considerations” section. The manuscript now specifies that the study was approved by the Ethical Review Board of the Holy Spirit University of Kaslik (USEK), Jounieh, Lebanon (Approval number: EN-CETH-001). Redundant ethical statements were removed from the Methods introduction to improve manuscript organization and avoid repetition.

Comment 5

Two variables “Feeling adequately prepared for UC” and “Preferred method of training” are included in demographic tables, however, they do not appropriate fit as demographic characteristics. You may consider reporting them with satisfaction finding.

Response: These variables were removed from the demographic characteristics table and reorganized into a separate section addressing participant preparedness and training preferences, alongside satisfaction-related findings, to improve the conceptual consistency of the Results section.

Comment 6

The initial declarative knowledge is provided in the narrative and then indicates that these are also presented in a table 3. However, the corresponding table 3 does not display the pre-test scores as stated. Instead, table 3 displays the changes in the OSATS scores for TS and NTS before and after training. Please address this across the results to ensure consistency between texts and corresponding tables.

Response: The Results section and tables were revised for consistency and clarity. The updated Table 3 now presents both pre- and post-training declarative knowledge responses side by side, together with McNemar test p-values, allowing direct comparison of knowledge acquisition following the intervention.

Comment 7

While the summary of overall and change in knowledge scores (Table 5.) is appropriately presented with statistical comparison, the item-level table reports only pre-training responses (Table 4). Given that post-training data are available, it would improve clarity and interpretability to present both pre- and post-training responses side by side for each item. This would allow readers to better understand which specific knowledge areas improved following the intervention.

Response: In response, the item-level knowledge table was completely revised. The updated table now presents both pre-training and post-training correct responses for each knowledge item in a side-by-side format, together with corresponding McNemar p-values. This revision substantially improves interpretability and allows readers to identify the specific knowledge domains that improved after the simulation-based intervention.

Comment 8

Also, the item-level presentation of responses is informative; however, reporting all response options for each question may reduce clarity. Consider simplifying the table to present the proportion of correct responses for each item (pre- and post-training), which would more directly reflect knowledge acquisition. Detailed response distributions could be included as supplementary material if needed.

Response: We agree with the reviewer's suggestion. The item-level knowledge table was simplified to report only the proportion of correct responses before and after training for each question, rather than displaying all response options. This improved readability and allowed the table to more directly reflect declarative knowledge acquisition following the intervention. Detailed response distributions were removed from the main manuscript.

Comment 9

In the section "transfer of learning outcomes", the results are reported thought these are difficult to interpret. In particular, it is unclear how the proportions in the table were derived and the denominator used. The total number of urethral catheterisations performed collectively is reported as 274, however, it would be helpful to include the distribution of procedures per medical intern (e.g., range and/or median with IQR) to better understand variability in individual procedural exposure within the six months post training.

Response: The “Transfer of learning outcomes” section and accompanying tables were revised extensively to improve clarity and interpretability. Specifically, explanatory footnotes were added to clarify the denominators used for each percentage calculation:

- procedure outcome percentages were calculated based on the total number of catheterization attempts,
- causes of failed attempts were calculated relative to total failed attempts,
- complication percentages were calculated relative to total complications.

This clarification has now been explicitly stated in the revised table footnotes and Results section.

Comment 10

It would be helpful to include the distribution of procedures per medical intern (e.g., range and/or median with IQR) to better understand variability in individual procedural exposure.

Response: In response, additional descriptive data regarding procedural exposure were added to the Results section. We now report that the median number of urethral catheterizations performed per intern during the six-month follow-up period was 5 [IQR: 4–10], demonstrating variability in individual clinical exposure following the training intervention.

We again sincerely thank the reviewer for the insightful comments and recommendations, which significantly improved the quality, clarity, and scientific presentation of our manuscript.

Sincerely,

Ramy Touma Sawaya, MD

(on behalf of all coauthors
