# Supplementary material for: Enhancing medical intern competence in urethral catheterization: Impact of simulation-based training on knowledge, self-efficacy, and clinical outcomes
Source: PLoS One. 2026 Jul 17;21(7):e0353677. doi: 10.1371/journal.pone.0353677 (PMC13379000; doi:10.1371/journal.pone.0353677)
Supplement: Attachment — Submitted filename: Reviewer_1 Feedback.docx [file pone.0353677.s004.docx]

**Feedback to authors**

Thank you for the opportunity to review the manuscript – *“Enhancing Intern Competence in Urethral Catheterization: Impact of Simulation-Based Training on Knowledge, Self-Efficacy, and Clinical Outcomes*”. The manuscript addresses an important topic on medical interns’ competency in urethral catheterisation, thus its relevance to clinical skills training.

Having reviewed the article, I have provided detailed feedback in the document (as comments). However, below is a summary on some areas that need clarification/improvement.

1) **Terminology and wording**

- The term “intern” is used throughout the manuscript; however, this may be ambiguous, as it can refer to different healthcare professions (e.g., nursing, pharmacy, or medicine). For clarity and precision, I recommend specifying “medical intern” in the title, and in the content for the first mention.
- Some statements need revision, as detailed in the document. For example, the phrase “… questionnaire conducted ….” should be revised to “… questionnaire administered ….”

2) **Methods**

- It is indicated that training was provide to the new interns, however, it is not specified at what point of internship. This is only mentioned in the discussion, please consider clarifying when (specific time) the medical interns were recruited and trained in the methods section.
- The ethics statement (in the Methods introduction) indicates approval by the “University Institutional Review Board,” however the specific university/institution is not identified. For clarity and transparency, please specify the name of the university (and include the approval reference number if available). Also, the ethical statements are repeated in both the Methods introduction and Ethical considerations sections. These can be consolidated under Ethics consideration section:
  - “The study procedures were conducted in accordance with the ethical principles outlined in the Declaration of Helsinki” and “Written informed consent was obtained from all participants before participation”

3) **Results**

- Two variables “*Feeling adequately prepared for UC*” and “*Preferred method of training*” are included in demographic tables, however, they do not appropriate fit as demographic characteristics. You may consider reporting them with satisfaction finding.
- The initial declarative knowledge is provided in the narrative and then indicates that these are also presented in a table 3. However, the corresponding table 3 does not display the pre-test scores as stated. Instead, table 3 displays the changes in the OSATS scores for TS and NTS before and after training. Please address this across the results to ensure consistency between texts and corresponding tables.
- While the summary of overall and change in knowledge scores (Table 5.) is appropriately presented with statistical comparison, the item-level table reports only pre-training responses (Table 4). Given that post-training data are available, it would improve clarity and interpretability to present both pre- and post-training responses side by side for each item. This would allow readers to better understand which specific knowledge areas improved following the intervention.
- Also, the item-level presentation of responses is informative; however, reporting all response options for each question may reduce clarity. Consider simplifying the table to present the proportion of correct responses for each item (pre- and post-training), which would more directly reflect knowledge acquisition. Detailed response distributions could be included as supplementary material if needed.

An example of suggested table of results on knowledge

Table 1. Proportion of medical interns answering each knowledge item correctly pre and post training

| **Knowledge item** | **Pre-training**  **n (%)** | **Post-training**  **n (%)** |
| --- | --- | --- |
| Q1 |  |  |
| Q2 |  |  |
| Q3 |  |  |
| Qn |  |  |

- In the section “*transfer of learning outcomes*”, the results are reported thought these are difficult to interpret. In particular, it is unclear how the proportions in the table were derived and the denominator used. The total number of urethral catheterisations performed collectively is reported as 274, however, it would be helpful to include the distribution of procedures per medical intern (e.g., range and/or median with IQR) to better understand variability in individual procedural exposure within the six months post training.

An example of suggested table of results on outcomes of UC

Table xx. Outcomes of urethral catheterisation attempts among medical interns 6 months post-training

| **Outcome category** | **n** | **%** |
| --- | --- | --- |
| **Urethral catheterisation procedures performed (n=274)** |  |  |
| Successful |  |  |
| Failed |  |  |
| **Reasons for failed UC attempts (n=19)** |  |  |
| Difficulty identifying urethral meatus (Female) |  |  |
| Phimosis (Male) |  |  |
| Inadequate exposure of the genitals |  |  |
| Stenosis of the urethral meatus |  |  |
| **Complications (n=11)** |  |  |
| Urethrorrhagia with spontaneous resolution |  |  |
| False passage creation |  |  |
| Urinary tract infection |  |  |
| Need for suprapubic catheterization |  |  |
| ***Note:***   - *% for procedure outcomes are based on total catheterisation attempts* - *% for reasons for failed attempts are based on total failed attempts* - *% for complications are based on total complications* | | |
