# Supplementary material for: Enhancing medical intern competence in urethral catheterization: Impact of simulation-based training on knowledge, self-efficacy, and clinical outcomes
Source: PLoS One. 2026 Jul 17;21(7):e0353677. doi: 10.1371/journal.pone.0353677 (PMC13379000; doi:10.1371/journal.pone.0353677)
Supplement: Attachment — Submitted filename: Reviewer_2 Feedback_2026.05.24.docx [file pone.0353677.s006.docx]

**Second round review feedback**

I thank the authors for addressing the major concerns raised during the initial review. The manuscript has improved substantially following revision.

However, many of the detailed tracked comments inserted directly within the manuscript during the first review round do not appear to have been fully addressed. These annotations contain some minor but important issues related to clarity, presentation, wording, and consistency. Rather than repeating all such points here, I encourage the authors to carefully revisit and address all tracked comments I provided in the annotated manuscript.

In addition, I noted the following issues during this revision round:

1. There should be uniformity in the reporting of figures and percentages throughout the manuscript (e.g., in table 3, the item: Where is the best position of the operator who is performing the UC? Pre and post values are reported as 17 (43) vs 17 (42.5)
2. Table 6 appears to be placed in a section that does not appropriately correspond with the related text discussion. Please check the placement and sequencing of all tables to ensure consistency with the narrative flow.
3. Although there was an overall improvement following training, a few knowledge items showed little or no improvement when comparing pre- and post-training percentage correct scores. This is an important finding and should be acknowledged in the Results and discussed further in the Discussion section. Examples include knowledge related to the best operator position, angle the penis should be stretched, correctly exposing the urethral meatus, and importance of volume of urine drained. Highlighting these areas may help identify components insufficiently addressed by the training intervention.
4. Participants were asked about their readiness to perform urethral catheterization, and pre-training results are reported. Were participants also assessed post-training? If so, the post-training findings and comparison with pre-training should also be reported.
5. Preferred method of training is reported for only 38 participants rather than the total sample of 40. Please account for the two missing participants.
6. Some additional clarity is needed in reporting “transfer of learning outcomes”. A success rate of 93% is described in the text; however, Table 7 reports urethral catheterization performed as 100%. If the 93% reflects successful attempts out of all attempts performed, the table should be revised or clarified to better align with the textual description. I appreciate the improvements made from the previous review, but clearer alignment between the text and tables would improve interpretation.
7. Kindly reconsider the placement of Table 6. It may fit more appropriately before Table 5b, particularly since the results on satisfaction are discussed immediately after preparedness and preferred training methods.

Further details on specific areas requiring attention are also indicated within the manuscript using tracked comments.

In general, the manuscript has been considerably improved and I have no further major concerns. I recommend that the authors to address the minor revisions identified above.
