# Supplementary material for: Enhancing medical intern competence in urethral catheterization: Impact of simulation-based training on knowledge, self-efficacy, and clinical outcomes
Source: PLoS One. 2026 Jul 17;21(7):e0353677. doi: 10.1371/journal.pone.0353677 (PMC13379000; doi:10.1371/journal.pone.0353677)
Supplement: Attachment — Submitted filename: Response 05.06.docx [file pone.0353677.s009.docx]

Dear Editor and Reviewer,

We would like to thank the reviewer for the careful evaluation of our revised manuscript and for the constructive comments provided throughout the review process. We appreciate the recognition that the manuscript has improved substantially following revision. We carefully revisited the annotated manuscript and addressed the remaining concerns related to clarity, presentation, wording, consistency, and data reporting. All changes have been incorporated into the revised manuscript.

Our responses to the specific comments are provided below.

1. **Uniformity in the reporting of figures and percentages throughout the manuscript.**

Response: We reviewed all tables and text for consistency in numerical reporting and corrected formatting discrepancies where identified. Percentages and decimal places have been standardized throughout the manuscript.

2. **Table placement and sequencing.**

Response: We reviewed the placement and sequencing of all tables. The table previously identified as being out of sequence was relocated to the appropriate section of the Results, ensuring better alignment with the corresponding narrative and improved manuscript flow.

3. Knowledge items showing little or no improvement following training.

Response: We agree that these findings are important and merit further discussion. Additional details regarding the domains that demonstrated minimal or no measurable improvement following training were added to the Results section. Furthermore, these findings are now discussed in greater detail in the Discussion section, including possible explanations and implications for future curriculum development.

4. **Reporting of post-training preparedness.**

Response: Post-training preparedness data were available and have now been incorporated into the Results section. This allows direct comparison with pre-training preparedness and provides a more complete assessment of the educational intervention.

5. **Missing participant responses regarding preferred training methods.**

Response: The two responses initially categorized as missing were reviewed. These responses had been entered as “other” but corresponded to existing response categories and were therefore reassigned appropriately. The table has been updated to reflect the complete dataset.

6. **Clarity of transfer-of-learning outcomes and alignment between text and tables.**

Response: We revised the transfer-of-learning outcomes section and corresponding table to improve clarity and consistency. The number and percentage of successful catheterization attempts (255; 93.1%) have been explicitly included in the table to align with the results reported in the text.

7. **Placement of the satisfaction table.**

Response: Following revision and restructuring of the Results section, the satisfaction table was repositioned to better correspond with the discussion of participant preparedness, preferred training methods, and post-training satisfaction outcomes.

**Additional comments from the annotated manuscript.**

Response: We carefully reviewed all tracked comments included within the annotated manuscript from the previous review round and addressed the identified issues. Specifically:

• The preparedness statement was moved from the demographic section to the participant satisfaction section.

• The demographic table was removed, and the relevant demographic information is now described directly within the text.

• References to supplementary material were verified and retained appropriately.

• Numerical formatting and decimal place inconsistencies were corrected.

• Findings demonstrating limited improvement following training were further described in both the Results and Discussion sections.

• Post-workshop preparedness outcomes were added to the Results section.

• Table placement and numbering were corrected following restructuring of the Results section.

• Reclassification of responses previously entered as “other” was performed where appropriate.

• The transfer-of-learning outcomes table was revised to include successful catheterization attempts and improve consistency between text and tables.

We believe that these revisions have strengthened the manuscript and addressed the reviewer’s remaining concerns. We thank the reviewer and editor for their time and consideration and hope that the revised manuscript is now suitable for publication in PLOS ONE.

Sincerely,

Maher Abdessater

On behalf of all co-authors
