# Supplementary material for: Enhancing medical intern competence in urethral catheterization: Impact of simulation-based training on knowledge, self-efficacy, and clinical outcomes
Source: PLoS One. 2026 Jul 17;21(7):e0353677. doi: 10.1371/journal.pone.0353677 (PMC13379000; doi:10.1371/journal.pone.0353677)
Supplement: Peer review history

# Peer review: Decision Letter 0

**DOI:** 10.1371/journal.pone.0353677.r001

**Authors:** Stanisław Wroński

5 Mar 2026

PONE-D-25-64830

Enhancing Intern Competence in Urethral Catheterization: Impact of Simulation-Based Training on Knowledge, Self-Efficacy, and Clinical Outcomes.

PLOS One

Dear Dr. Touma Sawaya,

Thank you for submitting your manuscript to PLOS ONE. After careful consideration, we feel that it has merit but does not fully meet PLOS ONE’s publication criteria as it currently stands. Therefore, we invite you to submit a revised version of the manuscript that addresses the points raised during the review process.

Please submit your revised manuscript by Apr 19 2026 11:59PM. If you will need more time than this to complete your revisions, please reply to this message or contact the journal office at plosone@plos.org. When you're ready to submit your revision, log on to https://www.editorialmanager.com/pone/ and select the 'Submissions Needing Revision' folder to locate your manuscript file.

Please include the following items when submitting your revised manuscript:

- A letter that responds to each point raised by the academic editor and reviewer(s). You should upload this letter as a separate file labeled 'Response to Reviewers'.
- A marked-up copy of your manuscript that highlights changes made to the original version. You should upload this as a separate file labeled 'Revised Manuscript with Track Changes'.
- An unmarked version of your revised paper without tracked changes. You should upload this as a separate file labeled 'Manuscript'.

If you would like to make changes to your financial disclosure, please include your updated statement in your cover letter. Guidelines for resubmitting your figure files are available below the reviewer comments at the end of this letter.

If applicable, we recommend that you deposit your laboratory protocols in protocols.io to enhance the reproducibility of your results. Protocols.io assigns your protocol its own identifier (DOI) so that it can be cited independently in the future. For instructions see: https://journals.plos.org/plosone/s/submission-guidelines#loc-laboratory-protocols. Additionally, PLOS ONE offers an option for publishing peer-reviewed Lab Protocol articles, which describe protocols hosted on protocols.io. Read more information on sharing protocols at https://plos.org/protocols?utm_medium=editorial-email&utm_source=authorletters&utm_campaign=protocols.

We look forward to receiving your revised manuscript.

Kind regards,

Stanisław Jacek Wroński, M.D., Ph.D, FEBU

Academic Editor

PLOS One

Journal Requirements:

When submitting your revision, we need you to address these additional requirements.

1.Please ensure that your manuscript meets PLOS ONE's style requirements, including those for file naming. The PLOS ONE style templates can be found at

https://journals.plos.org/plosone/s/file?id=wjVg/PLOSOne_formatting_sample_main_body.pdf and

https://journals.plos.org/plosone/s/file?id=ba62/PLOSOne_formatting_sample_title_authors_affiliations.pdf

2. We note that you have indicated that there are restrictions to data sharing for this study. PLOS only allows data to be available upon request if there are legal or ethical restrictions on sharing data publicly. For more information on unacceptable data access restrictions, please see http://journals.plos.org/plosone/s/data-availability#loc-unacceptable-data-access-restrictions.

Before we proceed with your manuscript, please address the following prompts:

a) If there are ethical or legal restrictions on sharing a de-identified data set, please explain them in detail (e.g., data contain potentially identifying or sensitive patient information, data are owned by a third-party organization, etc.) and who has imposed them (e.g., a Research Ethics Committee or Institutional Review Board, etc.). Please also provide contact information for a data access committee, ethics committee, or other institutional body to which data requests may be sent.

b) If there are no restrictions, please upload the minimal anonymized data set necessary to replicate your study findings to a stable, public repository and provide us with the relevant URLs, DOIs, or accession numbers. For a list of recommended repositories, please see

https://journals.plos.org/plosone/s/recommended-repositories. You also have the option of uploading the data as Supporting Information files, but we would recommend depositing data directly to a data repository if possible.

We will update your Data Availability statement on your behalf to reflect the information you provide.

3. Please include your full ethics statement in the ‘Methods’ section of your manuscript file. In your statement, please include the full name of the IRB or ethics committee who approved or waived your study, as well as whether or not you obtained informed written or verbal consent. If consent was waived for your study, please include this information in your statement as well.

4. Please upload a copy of Figures 1-3, to which you refer in your text on page 11. If the figure is no longer to be included as part of the submission please remove all reference to it within the text.

5. Please include a copy of Table 1-6 which you refer to in your text on page 7,10,11 and 12.

6. If the reviewer comments include a recommendation to cite specific previously published works, please review and evaluate these publications to determine whether they are relevant and should be cited. There is no requirement to cite these works unless the editor has indicated otherwise.

[Note: HTML markup is below. Please do not edit.]

Reviewers' comments:

Reviewer's Responses to Questions

**Comments to the Author**

1. Is the manuscript technically sound, and do the data support the conclusions?

The manuscript must describe a technically sound piece of scientific research with data that supports the conclusions. Experiments must have been conducted rigorously, with appropriate controls, replication, and sample sizes. The conclusions must be drawn appropriately based on the data presented.

Reviewer #1: Partly

**********

2. Has the statistical analysis been performed appropriately and rigorously?

Reviewer #1: No

**********

3. Have the authors made all data underlying the findings in their manuscript fully available?

The PLOS Data policy requires authors to make all data underlying the findings described in their manuscript fully available without restriction, with rare exception (please refer to the Data Availability Statement in the manuscript PDF file). The data should be provided as part of the manuscript or its supporting information, or deposited to a public repository. For example, in addition to summary statistics, the data points behind means, medians and variance measures should be available. If there are restrictions on publicly sharing data—e.g. participant privacy or use of data from a third party—those must be specified.

Reviewer #1: No

**********

4. Is the manuscript presented in an intelligible fashion and written in standard English?

PLOS ONE does not copyedit accepted manuscripts, so the language in submitted articles must be clear, correct, and unambiguous. Any typographical or grammatical errors should be corrected at revision, so please note any specific errors here.

Reviewer #1: Yes

**********

5. Review Comments to the Author

Please use the space provided to explain your answers to the questions above. You may also include additional comments for the author, including concerns about dual publication, research ethics, or publication ethics. (Please upload your review as an attachment if it exceeds 20,000 characters)

Reviewer #1: This manuscript addresses a relevant and practical training need (urethral catheterization) using a simulation-based workshop for new interns. The pre-post improvements in knowledge and self-reported outcomes are promising. To strengthen clarity, transparency, and reproducibility, several aspects of reporting and interpretation would benefit from further clarification. I therefore recommend major revision.

1. Interpretation in light of the study design

Given the single-group pre–post design, please consider softening causal language in the Discussion and Conclusions. In particular, it would be helpful to frame findings in descriptive terms and to acknowledge that other factors during the follow-up period (e.g., ongoing clinical exposure or supervision) may also have contributed to the observed changes.

2. Reporting of skills outcomes (OSATS)

OSATS is described as an important skills assessment. Please clarify whether pre- and post-training OSATS results are available and, if so, present summary statistics and the corresponding statistical analysis. Additional brief information on who performed the ratings and how assessments were conducted would help readers interpret these results.

3. Description of measurement tools

For the knowledge assessment, please provide additional detail on the test items and scoring (either in the main text or as Supporting Information), as well as how content relevance was established. For self-efficacy and confidence measures, please clarify whether these items were adapted from existing instruments or locally developed, and briefly justify their use.

4. Statistical analysis

Please clarify the rationale for using paired t-tests for Likert-scale outcomes, or consider alternative non-parametric analyses if more appropriate. Where feasible, reporting effect sizes with confidence intervals alongside p-values would further aid interpretation.

5. Six-month follow-up outcomes

Please define the criteria used for “success” and “complications” and briefly describe how follow-up data were collected and verified. Given the observational nature of these data, a more descriptive interpretation in the discussion would be appropriate.

6. Intervention description

To support reproducibility, please consider adding a few practical details on the workshop implementation (e.g., approximate instructor-to-learner ratio, hands-on practice time, and the general approach to feedback).

7. Data availability

Please review the data availability statement to ensure it aligns with PLOS ONE policies, for example, by indicating whether de-identified data or materials can be shared via a repository or other access mechanism.

8. Other

- Please ensure consistent use of key terms (e.g., self-efficacy/confidence).

- Check that all tables and figures referenced in the text are included and clearly labeled.

- Minor language editing would improve clarity in a few places.

**********

6. PLOS authors have the option to publish the peer review history of their article (what does this mean?). If published, this will include your full peer review and any attached files.

If you choose “no”, your identity will remain anonymous but your review may still be made public.

**Do you want your identity to be public for this peer review?**  For information about this choice, including consent withdrawal, please see our Privacy Policy.

Reviewer #1: No

**********

[NOTE: If reviewer comments were submitted as an attachment file, they will be attached to this email and accessible via the submission site. Please log into your account, locate the manuscript record, and check for the action link "View Attachments". If this link does not appear, there are no attachment files.]

To ensure your figures meet our technical requirements, please review our figure guidelines: https://journals.plos.org/plosone/s/figures

You may also use PLOS’s free figure tool, NAAS, to help you prepare publication quality figures: https://journals.plos.org/plosone/s/figures#loc-tools-for-figure-preparation.

NAAS will assess whether your figures meet our technical requirements by comparing each figure against our figure specifications.

# Author comment: Author response to Decision Letter 1

**DOI:** 10.1371/journal.pone.0353677.r002

6 Mar 2026

Dear Editors and Reviewers of PLOS ONE,

We would like to sincerely thank the Editor and the Reviewers for their careful evaluation of our manuscript and for the constructive comments provided. We have revised the manuscript accordingly and believe that these revisions have significantly improved the clarity, transparency, and scientific rigor of the work. Below we provide a detailed, point-by-point response to each comment. All corresponding changes have been incorporated into the revised manuscript.

1.Please ensure that your manuscript meets PLOS ONE's style requirements, including those for file naming. The PLOS ONE style templates can be found at

https://journals.plos.org/plosone/s/file?id=wjVg/PLOSOne_formatting_sample_main_body.pdf and https://journals.plos.org/plosone/s/file?id=ba62/PLOSOne_formatting_sample_title_authors_affiliations.pdf

Response: Thank you for this comment. The manuscript formatting has been revised to ensure full compliance with the formatting and style requirements of PLOS ONE, including file structure and labeling.

2. We note that you have indicated that there are restrictions to data sharing for this study. PLOS only allows data to be available upon request if there are legal or ethical restrictions on sharing data publicly. For more information on unacceptable data access restrictions, please see http://journals.plos.org/plosone/s/data-availability#loc-unacceptable-data-access-restrictions.

Before we proceed with your manuscript, please address the following prompts:

a) If there are ethical or legal restrictions on sharing a de-identified data set, please explain them in detail (e.g., data contain potentially identifying or sensitive patient information, data are owned by a third-party organization, etc.) and who has imposed them (e.g., a Research Ethics Committee or Institutional Review Board, etc.). Please also provide contact information for a data access committee, ethics committee, or other institutional body to which data requests may be sent.

b) If there are no restrictions, please upload the minimal anonymized data set necessary to replicate your study findings to a stable, public repository and provide us with the relevant URLs, DOIs, or accession numbers. For a list of recommended repositories, please see

https://journals.plos.org/plosone/s/recommended-repositories. You also have the option of uploading the data as Supporting Information files, but we would recommend depositing data directly to a data repository if possible.

We will update your Data Availability statement on your behalf to reflect the information you provide.

Response: The data was uploaded to the BioStudies repository

BioStudies accession number S-BSST2802.

https://eur03.safelinks.protection.outlook.com/?url=https%3A%2F%2Fwww.ebi.ac.uk%2Fbiostudies%2Fstudies%2FS-BSST2802&data=05%7C02%7Crt81%40aub.edu.lb%7C89f1c93fe78f4692331c08de7a87a164%7Cc7ba5b1a41b643e9a1206ff654ada137%7C1%7C0%7C639082920124036026%7CUnknown%7CTWFpbGZsb3d8eyJFbXB0eU1hcGkiOnRydWUsIlYiOiIwLjAuMDAwMCIsIlAiOiJXaW4zMiIsIkFOIjoiTWFpbCIsIldUIjoyfQ%3D%3D%7C0%7C%7C%7C&sdata=fiNqcpaUc92wuBwiTM6DChlmWvQakco0eiI0pumJ870%3D&reserved=0

The dataset is available under accession number S-BSST2802 and can be accessed at:

https://www.ebi.ac.uk/biostudies/studies/S-BSST2802

The Data Availability Statement in the manuscript has been updated accordingly.

3. Please include your full ethics statement in the ‘Methods’ section of your manuscript file. In your statement, please include the full name of the IRB or ethics committee who approved or waived your study, as well as whether or not you obtained informed written or verbal consent. If consent was waived for your study, please include this information in your statement as well.

Response: Thank you for this important suggestion. We have now expanded the ethics statement in the Methods section to include the full name of the approving ethics committee. The manuscript now specifies that the study was approved by the Ethical Review Board of the Holy Spirit University of Kaslik (USEK), Jounieh, Lebanon, and that written informed consent was obtained from all participants prior to enrollment. The study’s compliance with the principles of the Declaration of Helsinki has also been clarified.

4. Please upload a copy of Figures 1-3, to which you refer in your text on page 11. If the figure is no longer to be included as part of the submission please remove all reference to it within the text.

Response: Thank you for this observation. These figures were not intended to be included in the final submission; therefore, all references to Figures 1–3 have been removed from the manuscript.

5. Please include a copy of Table 1-6 which you refer to in your text on page 7,10,11 and 12.

Response: Tables 1–7 have now been included in the revised manuscript and appropriately labeled. We also corrected a referencing error in the text where Table 6 was cited twice; the second citation has now been corrected to Table 7.

Reviewer Comments to the Author

Reviewer #1: This manuscript addresses a relevant and practical training need (urethral catheterization) using a simulation-based workshop for new interns. The pre-post improvements in knowledge and self-reported outcomes are promising. To strengthen clarity, transparency, and reproducibility, several aspects of reporting and interpretation would benefit from further clarification. I therefore recommend major revision.

1. Interpretation in light of the study design

Given the single-group pre–post design, please consider softening causal language in the Discussion and Conclusions. In particular, it would be helpful to frame findings in descriptive terms and to acknowledge that other factors during the follow-up period (e.g., ongoing clinical exposure or supervision) may also have contributed to the observed changes.

Response: We appreciate this important comment. The Discussion and Conclusion sections have been revised to adopt more descriptive language when interpreting the findings. We now explicitly acknowledge that improvements observed during follow-up may also reflect ongoing clinical exposure, supervision by senior physicians, and routine clinical practice during the internship period. These revisions clarify that the observed improvements cannot be attributed solely to the simulation intervention.

2. Reporting of skills outcomes (OSATS)

OSATS is described as an important skills assessment. Please clarify whether pre- and post-training OSATS results are available and, if so, present summary statistics and the corresponding statistical analysis. Additional brief information on who performed the ratings and how assessments were conducted would help readers interpret these results.

Response: Pre- and post-training Objective Structured Assessment of Technical Skills (OSATS) evaluations were indeed conducted. The corresponding summary statistics and statistical comparisons are now reported in Table 3.

To further improve clarity, the Methods section now includes additional details regarding the evaluation process. Catheterization procedures were assessed by senior urology residents supervising the simulation sessions, using the standardized OSATS rubric. Participants were evaluated before the educational intervention and again following completion of the training, allowing direct comparison of technical performance.

3. Description of measurement tools

For the knowledge assessment, please provide additional detail on the test items and scoring (either in the main text or as Supporting Information), as well as how content relevance was established. For self-efficacy and confidence measures, please clarify whether these items were adapted from existing instruments or locally developed, and briefly justify their use.

Response: We have expanded the Methods section to provide further detail regarding the knowledge assessment and scoring system. The questionnaire consisted of multiple-choice and open-ended questions addressing key aspects of urethral catheterization, including indications, contraindications, catheter types and sizes, patient positioning, sterile technique, procedural steps, and complication prevention. Each correct response was assigned one point, generating a cumulative knowledge score.

The questionnaire content was developed based on established urological educational resources, including Campbell-Walsh Urology, to ensure content relevance.

The self-efficacy and confidence measures were locally developed for this educational evaluation and utilized Likert-type scales commonly used in simulation-based education research to capture participants’ perceived preparedness and confidence before and after training.

4. Statistical analysis

Please clarify the rationale for using paired t-tests for Likert-scale outcomes, or consider alternative non-parametric analyses if more appropriate. Where feasible, reporting effect sizes with confidence intervals alongside p-values would further aid interpretation.

Response: In this study, Likert-scale outcomes (perceived self-efficacy and confidence) were treated as approximately continuous variables and summarized using means and standard deviations, which is a commonly accepted approach in educational research when evaluating within-participant pre-post changes. Accordingly, paired t-tests were used for statistical comparison.

To improve interpretability, the Methods section now clarifies this rationale, and effect size estimates with 95% confidence intervals have been added to the Results section alongside the reported p-values.

5. Six-month follow-up outcomes

Please define the criteria used for “success” and “complications” and briefly describe how follow-up data were collected and verified. Given the observational nature of these data, a more descriptive interpretation in the discussion would be appropriate.

Response: We have clarified these definitions in the Methods section.

Procedural success was defined as successful insertion of the urinary catheter with adequate urine drainage without the need for assistance or alternative catheterization techniques.

Complications included adverse events occurring during or immediately after catheterization, such as urethrorrhagia, false passage creation, urinary tract infection, or the need for suprapubic catheterization.

We also clarified that six-month follow-up data were obtained from intern procedural logbooks and confirmed with supervising urology residents. The Discussion section has been revised to present these findings in a descriptive manner, acknowledging their observational nature.

6. Intervention description

To support reproducibility, please consider adding a few practical details on the workshop implementation (e.g., approximate instructor-to-learner ratio, hands-on practice time, and the general approach to feedback).

Response: The description of the simulation workshop has been expanded in the Methods section to include additional practical details, including the instructor-to-learner ratio, duration of hands-on practice, and the structured feedback approach used during the training sessions. These additions aim to improve the reproducibility of the educational intervention.

7. Data availability

Please review the data availability statement to ensure it aligns with PLOS ONE policies, for example, by indicating whether de-identified data or materials can be shared via a repository or other access mechanism.

Response: The Data Availability Statement has been updated to comply with PLOS ONE policies. The de-identified dataset supporting this study has been deposited in the public repository BioStudies, hosted by the European Bioinformatics Institute, under accession number S-BSST2802.

8. Other

- Please ensure consistent use of key terms (e.g., self-efficacy/confidence).

- Check that all tables and figures referenced in the text are included and clearly labeled.

- Minor language editing would improve clarity in a few places.

Response: We have carefully reviewed the manuscript to ensure consistent terminology, particularly regarding perceived self-efficacy and confidence. All tables and figures referenced in the manuscript have been verified and correctly labeled. The manuscript has also undergone minor language editing to improve clarity and readability.

We thank the Editor and Reviewers again for their valuable comments and insightful suggestions. We believe that the revisions made in response to these comments have strengthened the manuscript and improved its clarity and methodological transparency.

We appreciate your consideration of our revised manuscript.

Sincerely,

Maher Abdessater

On behalf of all co-authors

Attachment Submitted filename: Review response Foley.pdf

# Peer review: Decision Letter 1

**DOI:** 10.1371/journal.pone.0353677.r003

**Authors:** Stanisław Wroński, Stanisław Wroński

14 May 2026

PONE-D-25-64830R1

Enhancing Intern Competence in Urethral Catheterization: Impact of Simulation-Based Training on Knowledge, Self-Efficacy, and Clinical Outcomes.

PLOS One

Dear Dr. Touma Sawaya,

Thank you for submitting your manuscript to PLOS ONE. After careful consideration, we feel that it has merit but does not fully meet PLOS ONE’s publication criteria as it currently stands. Therefore, we invite you to submit a revised version of the manuscript that addresses the points raised during the review process.

Please submit your revised manuscript by Jun 28 2026 11:59PM. If you will need more time than this to complete your revisions, please reply to this message or contact the journal office at plosone@plos.org. When you're ready to submit your revision, log on to https://www.editorialmanager.com/pone/ and select the 'Submissions Needing Revision' folder to locate your manuscript file.

Please include the following items when submitting your revised manuscript:

- A letter that responds to each point raised by the academic editor and reviewer(s). You should upload this letter as a separate file labeled 'Response to Reviewers'.
- A marked-up copy of your manuscript that highlights changes made to the original version. You should upload this as a separate file labeled 'Revised Manuscript with Track Changes'.
- An unmarked version of your revised paper without tracked changes. You should upload this as a separate file labeled 'Manuscript'.

If you would like to make changes to your financial disclosure, please include your updated statement in your cover letter. Guidelines for resubmitting your figure files are available below the reviewer comments at the end of this letter.

If applicable, we recommend that you deposit your laboratory protocols in protocols.io to enhance the reproducibility of your results. Protocols.io assigns your protocol its own identifier (DOI) so that it can be cited independently in the future. For instructions see: https://journals.plos.org/plosone/s/submission-guidelines#loc-laboratory-protocols. Additionally, PLOS ONE offers an option for publishing peer-reviewed Lab Protocol articles, which describe protocols hosted on protocols.io. Read more information on sharing protocols at https://plos.org/protocols?utm_medium=editorial-email&utm_source=authorletters&utm_campaign=protocols.

As the corresponding author, your ORCID iD is verified in the submission system and will appear in the published article. PLOS supports the use of ORCID, and we encourage all coauthors to register for an ORCID iD and use it as well. Please encourage your coauthors to verify their ORCID iD within the submission system before final acceptance, as unverified ORCID iDs will not appear in the published article. *Only*  the individual author can complete the verification step; PLOS staff *cannot*  verify ORCID iDs on behalf of authors.

We look forward to receiving your revised manuscript.

Kind regards,

Stanisław Jacek Wroński, M.D., Ph.D, FEBU

Academic Editor

PLOS One

Journal Requirements:

1. If the reviewer comments include a recommendation to cite specific previously published works, please review and evaluate these publications to determine whether they are relevant and should be cited. There is no requirement to cite these works unless the editor has indicated otherwise.

2. Please review your reference list to ensure that it is complete and correct. If you have cited papers that have been retracted, please include the rationale for doing so in the manuscript text, or remove these references and replace them with relevant current references. Any changes to the reference list should be mentioned in the rebuttal letter that accompanies your revised manuscript. If you need to cite a retracted article, indicate the article’s retracted status in the References list and also include a citation and full reference for the retraction notice.

Reviewers' comments:

Reviewer's Responses to Questions

**Comments to the Author**

1. If the authors have adequately addressed your comments raised in a previous round of review and you feel that this manuscript is now acceptable for publication, you may indicate that here to bypass the “Comments to the Author” section, enter your conflict of interest statement in the “Confidential to Editor” section, and submit your "Accept" recommendation.

Reviewer #2: (No Response)

**********

2. Is the manuscript technically sound, and do the data support the conclusions?

The manuscript must describe a technically sound piece of scientific research with data that supports the conclusions. Experiments must have been conducted rigorously, with appropriate controls, replication, and sample sizes. The conclusions must be drawn appropriately based on the data presented.

Reviewer #2: Yes

**********

3. Has the statistical analysis been performed appropriately and rigorously?

Reviewer #2: No

**********

4. Have the authors made all data underlying the findings in their manuscript fully available?

The PLOS Data policy requires authors to make all data underlying the findings described in their manuscript fully available without restriction, with rare exception (please refer to the Data Availability Statement in the manuscript PDF file). The data should be provided as part of the manuscript or its supporting information, or deposited to a public repository. For example, in addition to summary statistics, the data points behind means, medians and variance measures should be available. If there are restrictions on publicly sharing data—e.g. participant privacy or use of data from a third party—those must be specified.

Reviewer #2: No

**********

5. Is the manuscript presented in an intelligible fashion and written in standard English?

PLOS ONE does not copyedit accepted manuscripts, so the language in submitted articles must be clear, correct, and unambiguous. Any typographical or grammatical errors should be corrected at revision, so please note any specific errors here.

Reviewer #2: Yes

**********

6. Review Comments to the Author

Please use the space provided to explain your answers to the questions above. You may also include additional comments for the author, including concerns about dual publication, research ethics, or publication ethics. (Please upload your review as an attachment if it exceeds 20,000 characters)

Reviewer #2: I have provided feedback to the authors to do some revision to the manuscript (see attached feedback)

**********

7. PLOS authors have the option to publish the peer review history of their article (what does this mean?). If published, this will include your full peer review and any attached files.

If you choose “no”, your identity will remain anonymous but your review may still be made public.

**Do you want your identity to be public for this peer review?**  For information about this choice, including consent withdrawal, please see our Privacy Policy.

Reviewer #2: **Yes:**  Kelvin Oruko

**********

[NOTE: If reviewer comments were submitted as an attachment file, they will be attached to this email and accessible via the submission site. Please log into your account, locate the manuscript record, and check for the action link "View Attachments". If this link does not appear, there are no attachment files.]

To ensure your figures meet our technical requirements, please review our figure guidelines: https://journals.plos.org/plosone/s/figures

You may also use PLOS’s free figure tool, NAAS, to help you prepare publication quality figures: https://journals.plos.org/plosone/s/figures#loc-tools-for-figure-preparation.

NAAS will assess whether your figures meet our technical requirements by comparing each figure against our figure specifications.

Attachment Submitted filename: PONE-D-25-64830_Reviewer_1_2026.05.06.pdf

Attachment Submitted filename: Reviewer_1 Feedback.docx

# Author comment: Author response to Decision Letter 2

**DOI:** 10.1371/journal.pone.0353677.r004

22 May 2026

Response to Reviewer 1

We sincerely thank the reviewer for the thoughtful and constructive comments. We greatly appreciate the time and effort dedicated to reviewing our manuscript. The suggestions have significantly improved the clarity, organization, and scientific rigor of the paper. All comments were carefully considered and addressed as detailed below.

Comment 1

The term “intern” is used throughout the manuscript; however, this may be ambiguous, as it can refer to different healthcare professions (e.g., nursing, pharmacy, or medicine). For clarity and precision, I recommend specifying “medical intern” in the title, and in the content for the first mention.

Response: The terminology has been revised throughout the manuscript to specify “medical intern” where appropriate, including in the title and at first mention within the text, in order to improve clarity and avoid ambiguity regarding the study population.

Comment 2

Some statements need revision, as detailed in the document. For example, the phrase “… questionnaire conducted ….” should be revised to “… questionnaire administered ….”

Response: The manuscript has undergone thorough linguistic revision, and the suggested wording changes, including replacing “questionnaire conducted” with “questionnaire administered,” have been implemented throughout the manuscript to improve readability and academic style.

Comment 3

It is indicated that training was provide to the new interns, however, it is not specified at what point of internship. This is only mentioned in the discussion, please consider clarifying when (specific time) the medical interns were recruited and trained in the methods section.

Response: The Methods section has been revised to clarify the timing of recruitment and training. Specifically, we now state that the simulation workshop was conducted at the beginning of the interns’ academic year, prior to substantial clinical exposure, as part of their early internship orientation and training.

Comment 4

The ethics statement (in the Methods introduction) indicates approval by the “University Institutional Review Board,” however the specific university/institution is not identified. For clarity and transparency, please specify the name of the university (and include the approval reference number if available). Also, the ethical statements are repeated in both the Methods introduction and Ethical considerations sections. These can be consolidated under Ethics consideration section:

Response: The ethical approval statement has been clarified and consolidated within the dedicated “Ethical considerations” section. The manuscript now specifies that the study was approved by the Ethical Review Board of the Holy Spirit University of Kaslik (USEK), Jounieh, Lebanon (Approval number: EN-CETH-001). Redundant ethical statements were removed from the Methods introduction to improve manuscript organization and avoid repetition.

Comment 5

Two variables “Feeling adequately prepared for UC” and “Preferred method of training” are included in demographic tables, however, they do not appropriate fit as demographic characteristics. You may consider reporting them with satisfaction finding.

Response: These variables were removed from the demographic characteristics table and reorganized into a separate section addressing participant preparedness and training preferences, alongside satisfaction-related findings, to improve the conceptual consistency of the Results section.

Comment 6

The initial declarative knowledge is provided in the narrative and then indicates that these are also presented in a table 3. However, the corresponding table 3 does not display the pre-test scores as stated. Instead, table 3 displays the changes in the OSATS scores for TS and NTS before and after training. Please address this across the results to ensure consistency between texts and corresponding tables.

Response: The Results section and tables were revised for consistency and clarity. The updated Table 3 now presents both pre- and post-training declarative knowledge responses side by side, together with McNemar test p-values, allowing direct comparison of knowledge acquisition following the intervention.

Comment 7

While the summary of overall and change in knowledge scores (Table 5.) is appropriately presented with statistical comparison, the item-level table reports only pre-training responses (Table 4). Given that post-training data are available, it would improve clarity and interpretability to present both pre- and post-training responses side by side for each item. This would allow readers to better understand which specific knowledge areas improved following the intervention.

Response: In response, the item-level knowledge table was completely revised. The updated table now presents both pre-training and post-training correct responses for each knowledge item in a side-by-side format, together with corresponding McNemar p-values. This revision substantially improves interpretability and allows readers to identify the specific knowledge domains that improved after the simulation-based intervention.

Comment 8

Also, the item-level presentation of responses is informative; however, reporting all response options for each question may reduce clarity. Consider simplifying the table to present the proportion of correct responses for each item (pre- and post-training), which would more directly reflect knowledge acquisition. Detailed response distributions could be included as supplementary material if needed.

Response: We agree with the reviewer’s suggestion. The item-level knowledge table was simplified to report only the proportion of correct responses before and after training for each question, rather than displaying all response options. This improved readability and allowed the table to more directly reflect declarative knowledge acquisition following the intervention. Detailed response distributions were removed from the main manuscript.

Comment 9

In the section “transfer of learning outcomes”, the results are reported thought these are difficult to interpret. In particular, it is unclear how the proportions in the table were derived and the denominator used. The total number of urethral catheterisations performed collectively is reported as 274, however, it would be helpful to include the distribution of procedures per medical intern (e.g., range and/or median with IQR) to better understand variability in individual procedural exposure within the six months post training.

Response: The “Transfer of learning outcomes” section and accompanying tables were revised extensively to improve clarity and interpretability. Specifically, explanatory footnotes were added to clarify the denominators used for each percentage calculation:

procedure outcome percentages were calculated based on the total number of catheterization attempts,

causes of failed attempts were calculated relative to total failed attempts,

complication percentages were calculated relative to total complications.

This clarification has now been explicitly stated in the revised table footnotes and Results section.

Comment 10

It would be helpful to include the distribution of procedures per medical intern (e.g., range and/or median with IQR) to better understand variability in individual procedural exposure.

Response: In response, additional descriptive data regarding procedural exposure were added to the Results section. We now report that the median number of urethral catheterizations performed per intern during the six-month follow-up period was 5 [IQR: 4–10], demonstrating variability in individual clinical exposure following the training intervention.

We again sincerely thank the reviewer for the insightful comments and recommendations, which significantly improved the quality, clarity, and scientific presentation of our manuscript.

Sincerely,

Ramy Touma Sawaya, MD

(on behalf of all coauthors

Attachment Submitted filename: Response to Reviewer 1.pdf

# Peer review: Decision Letter 2

**DOI:** 10.1371/journal.pone.0353677.r005

**Authors:** Stanisław Wroński, Stanisław Wroński, Stanisław Wroński

4 Jun 2026

PONE-D-25-64830R2

Enhancing Medical Intern Competence in Urethral Catheterization: Impact of Simulation-Based Training on Knowledge, Self-Efficacy, and Clinical Outcomes.

PLOS One

Dear Dr. Touma Sawaya,

Thank you for submitting your manuscript to PLOS ONE. After careful consideration, we feel that it has merit but does not fully meet PLOS ONE’s publication criteria as it currently stands. Therefore, we invite you to submit a revised version of the manuscript that addresses the points raised during the review process.

Please submit your revised manuscript by Jul 19 2026 11:59PM If you will need more time than this to complete your revisions, please reply to this message or contact the journal office at plosone@plos.org. When you're ready to submit your revision, log on to https://www.editorialmanager.com/pone/ and select the 'Submissions Needing Revision' folder to locate your manuscript file.

Please include the following items when submitting your revised manuscript:

- A letter that responds to each point raised by the academic editor and reviewer(s). You should upload this letter as a separate file labeled 'Response to Reviewers'.
- A marked-up copy of your manuscript that highlights changes made to the original version. You should upload this as a separate file labeled 'Revised Manuscript with Track Changes'.
- An unmarked version of your revised paper without tracked changes. You should upload this as a separate file labeled 'Manuscript'.

If you would like to make changes to your financial disclosure, please include your updated statement in your cover letter. Guidelines for resubmitting your figure files are available below the reviewer comments at the end of this letter.

If applicable, we recommend that you deposit your laboratory protocols in protocols.io to enhance the reproducibility of your results. Protocols.io assigns your protocol its own identifier (DOI) so that it can be cited independently in the future. For instructions see: https://journals.plos.org/plosone/s/submission-guidelines#loc-laboratory-protocols. Additionally, PLOS ONE offers an option for publishing peer-reviewed Lab Protocol articles, which describe protocols hosted on protocols.io. Read more information on sharing protocols at https://plos.org/protocols?utm_medium=editorial-email&utm_source=authorletters&utm_campaign=protocols.

As the corresponding author, your ORCID iD is verified in the submission system and will appear in the published article. PLOS supports the use of ORCID, and we encourage all coauthors to register for an ORCID iD and use it as well. Please encourage your coauthors to verify their ORCID iD within the submission system before final acceptance, as unverified ORCID iDs will not appear in the published article. *Only*  the individual author can complete the verification step; PLOS staff *cannot*  verify ORCID iDs on behalf of authors.

We look forward to receiving your revised manuscript.

Kind regards,

Stanisław Jacek Wroński, M.D., Ph.D, FEBU

Academic Editor

PLOS One

Journal Requirements:

If the reviewer comments include a recommendation to cite specific previously published works, please review and evaluate these publications to determine whether they are relevant and should be cited. There is no requirement to cite these works unless the editor has indicated otherwise.

Please review your reference list to ensure that it is complete and correct. If you have cited papers that have been retracted, please include the rationale for doing so in the manuscript text, or remove these references and replace them with relevant current references. Any changes to the reference list should be mentioned in the rebuttal letter that accompanies your revised manuscript. If you need to cite a retracted article, indicate the article’s retracted status in the References list and also include a citation and full reference for the retraction notice.

**Comments to the Author**

1. If the authors have adequately addressed your comments raised in a previous round of review and you feel that this manuscript is now acceptable for publication, you may indicate that here to bypass the “Comments to the Author” section, enter your conflict of interest statement in the “Confidential to Editor” section, and submit your "Accept" recommendation.

Reviewer #2: (No Response)

**********

2. Is the manuscript technically sound, and do the data support the conclusions?

The manuscript must describe a technically sound piece of scientific research with data that supports the conclusions. Experiments must have been conducted rigorously, with appropriate controls, replication, and sample sizes. The conclusions must be drawn appropriately based on the data presented.

Reviewer #2: Yes

**********

3. Has the statistical analysis been performed appropriately and rigorously?

Reviewer #2: Yes

**********

4. Have the authors made all data underlying the findings in their manuscript fully available?

The PLOS Data policy requires authors to make all data underlying the findings described in their manuscript fully available without restriction, with rare exception (please refer to the Data Availability Statement in the manuscript PDF file). The data should be provided as part of the manuscript or its supporting information, or deposited to a public repository. For example, in addition to summary statistics, the data points behind means, medians and variance measures should be available. If there are restrictions on publicly sharing data—e.g. participant privacy or use of data from a third party—those must be specified.

Reviewer #2: Yes

**********

5. Is the manuscript presented in an intelligible fashion and written in standard English?

PLOS ONE does not copyedit accepted manuscripts, so the language in submitted articles must be clear, correct, and unambiguous. Any typographical or grammatical errors should be corrected at revision, so please note any specific errors here.

Reviewer #2: Yes

**********

6. Review Comments to the Author

Please use the space provided to explain your answers to the questions above. You may also include additional comments for the author, including concerns about dual publication, research ethics, or publication ethics. (Please upload your review as an attachment if it exceeds 20,000 characters)

Reviewer #2: I have provided review feedback for the authors to consider (attached) and further details on specific areas requiring attention are also indicated in the annotated manuscript.

**********

7. PLOS authors have the option to publish the peer review history of their article (what does this mean?). If published, this will include your full peer review and any attached files.

If you choose “no”, your identity will remain anonymous but your review may still be made public.

**Do you want your identity to be public for this peer review?**  For information about this choice, including consent withdrawal, please see our Privacy Policy.

Reviewer #2: **Yes:**  Kelvin Oruko

**********

[NOTE: If reviewer comments were submitted as an attachment file, they will be attached to this email and accessible via the submission site. Please log into your account, locate the manuscript record, and check for the action link "View Attachments". If this link does not appear, there are no attachment files.]

To ensure your figures meet our technical requirements, please review our figure guidelines: https://journals.plos.org/plosone/s/figures

You may also use PLOS’s free figure tool, NAAS, to help you prepare publication quality figures: https://journals.plos.org/plosone/s/figures#loc-tools-for-figure-preparation.

NAAS will assess whether your figures meet our technical requirements by comparing each figure against our figure specifications.

Attachment Submitted filename: Reviewer_2 Feedback_2026.05.24.docx

Attachment Submitted filename: PONE-D-25-64830_R2_reviewer_2026.05.24.pdf

# Author comment: Author response to Decision Letter 3

**DOI:** 10.1371/journal.pone.0353677.r006

8 Jun 2026

Dear Editor and Reviewer,

We would like to thank the reviewer for the careful evaluation of our revised manuscript and for the constructive comments provided throughout the review process. We appreciate the recognition that the manuscript has improved substantially following revision. We carefully revisited the annotated manuscript and addressed the remaining concerns related to clarity, presentation, wording, consistency, and data reporting. All changes have been incorporated into the revised manuscript.

Our responses to the specific comments are provided below.

1. Uniformity in the reporting of figures and percentages throughout the manuscript.

Response: We reviewed all tables and text for consistency in numerical reporting and corrected formatting discrepancies where identified. Percentages and decimal places have been standardized throughout the manuscript.

2. Table placement and sequencing.

Response: We reviewed the placement and sequencing of all tables. The table previously identified as being out of sequence was relocated to the appropriate section of the Results, ensuring better alignment with the corresponding narrative and improved manuscript flow.

3. Knowledge items showing little or no improvement following training.

Response: We agree that these findings are important and merit further discussion. Additional details regarding the domains that demonstrated minimal or no measurable improvement following training were added to the Results section. Furthermore, these findings are now discussed in greater detail in the Discussion section, including possible explanations and implications for future curriculum development.

4. Reporting of post-training preparedness.

Response: Post-training preparedness data were available and have now been incorporated into the Results section. This allows direct comparison with pre-training preparedness and provides a more complete assessment of the educational intervention.

5. Missing participant responses regarding preferred training methods.

Response: The two responses initially categorized as missing were reviewed. These responses had been entered as “other” but corresponded to existing response categories and were therefore reassigned appropriately. The table has been updated to reflect the complete dataset.

6. Clarity of transfer-of-learning outcomes and alignment between text and tables.

Response: We revised the transfer-of-learning outcomes section and corresponding table to improve clarity and consistency. The number and percentage of successful catheterization attempts (255; 93.1%) have been explicitly included in the table to align with the results reported in the text.

7. Placement of the satisfaction table.

Response: Following revision and restructuring of the Results section, the satisfaction table was repositioned to better correspond with the discussion of participant preparedness, preferred training methods, and post-training satisfaction outcomes.

Additional comments from the annotated manuscript.

Response: We carefully reviewed all tracked comments included within the annotated manuscript from the previous review round and addressed the identified issues. Specifically:

• The preparedness statement was moved from the demographic section to the participant satisfaction section.

• The demographic table was removed, and the relevant demographic information is now described directly within the text.

• References to supplementary material were verified and retained appropriately.

• Numerical formatting and decimal place inconsistencies were corrected.

• Findings demonstrating limited improvement following training were further described in both the Results and Discussion sections.

• Post-workshop preparedness outcomes were added to the Results section.

• Table placement and numbering were corrected following restructuring of the Results section.

• Reclassification of responses previously entered as “other” was performed where appropriate.

• The transfer-of-learning outcomes table was revised to include successful catheterization attempts and improve consistency between text and tables.

We believe that these revisions have strengthened the manuscript and addressed the reviewer’s remaining concerns. We thank the reviewer and editor for their time and consideration and hope that the revised manuscript is now suitable for publication in PLOS ONE.

Sincerely,

Maher Abdessater

On behalf of all co-authors

Attachment Submitted filename: Response 05.06.docx

# Peer review: Decision Letter 3

**DOI:** 10.1371/journal.pone.0353677.r007

**Authors:** Stanisław Wroński, Stanisław Wroński, Stanisław Wroński, Stanisław Wroński

29 Jun 2026

Enhancing Medical Intern Competence in Urethral Catheterization: Impact of Simulation-Based Training on Knowledge, Self-Efficacy, and Clinical Outcomes.

PONE-D-25-64830R3

Dear Dr. Ramy Sawaya,

We’re pleased to inform you that your manuscript has been judged scientifically suitable for publication and will be formally accepted for publication once it meets all outstanding technical requirements.

Within one week, you’ll receive an e-mail detailing the required amendments. When these have been addressed, you’ll receive a formal acceptance letter and your manuscript will be scheduled for publication.

An invoice will be generated when your article is formally accepted. Please note, if your institution has a publishing partnership with PLOS and your article meets the relevant criteria, all or part of your publication costs will be covered. Please make sure your user information is up-to-date by logging into Editorial Manager at Editorial Manager® and clicking the ‘Update My Information' link at the top of the page. For questions related to billing, please contact billing support.

If your institution or institutions have a press office, please notify them about your upcoming paper to help maximize its impact. If they’ll be preparing press materials, please inform our press team as soon as possible -- no later than 48 hours after receiving the formal acceptance. Your manuscript will remain under strict press embargo until 2 pm Eastern Time on the date of publication. For more information, please contact onepress@plos.org.

Kind regards,

Stanisław Jacek Wroński, M.D., Ph.D, FEBU

Academic Editor

PLOS One

Dear Authors,

In accordance with the reviewers’ final decision, the submitted manuscript, in its revised version (R3), meets the requirements for publication in the journal PLOS ONE

with compliments

Stanisław Wroński

Academic Editor

Reviewers' comments:

Reviewer's Responses to Questions

**Comments to the Author**

1. If the authors have adequately addressed your comments raised in a previous round of review and you feel that this manuscript is now acceptable for publication, you may indicate that here to bypass the “Comments to the Author” section, enter your conflict of interest statement in the “Confidential to Editor” section, and submit your "Accept" recommendation.

Reviewer #2: All comments have been addressed

**********

2. Is the manuscript technically sound, and do the data support the conclusions?

The manuscript must describe a technically sound piece of scientific research with data that supports the conclusions. Experiments must have been conducted rigorously, with appropriate controls, replication, and sample sizes. The conclusions must be drawn appropriately based on the data presented.

Reviewer #2: Yes

**********

3. Has the statistical analysis been performed appropriately and rigorously?

Reviewer #2: Yes

**********

4. Have the authors made all data underlying the findings in their manuscript fully available?

The PLOS Data policy requires authors to make all data underlying the findings described in their manuscript fully available without restriction, with rare exception (please refer to the Data Availability Statement in the manuscript PDF file). The data should be provided as part of the manuscript or its supporting information, or deposited to a public repository. For example, in addition to summary statistics, the data points behind means, medians and variance measures should be available. If there are restrictions on publicly sharing data—e.g. participant privacy or use of data from a third party—those must be specified.

Reviewer #2: Yes

**********

5. Is the manuscript presented in an intelligible fashion and written in standard English?

PLOS ONE does not copyedit accepted manuscripts, so the language in submitted articles must be clear, correct, and unambiguous. Any typographical or grammatical errors should be corrected at revision, so please note any specific errors here.

Reviewer #2: Yes

**********

6. Review Comments to the Author

Please use the space provided to explain your answers to the questions above. You may also include additional comments for the author, including concerns about dual publication, research ethics, or publication ethics. (Please upload your review as an attachment if it exceeds 20,000 characters)

Reviewer #2: I thank the authors for their careful responses to the comments and for the revisions made, which have strengthened the manuscript. I am satisfied that the concerns raised during the previous review have been adequately addressed.

As a minor editorial point, the authors should ensure consistency in the reporting of summary statistics throughout the manuscript (e.g., means reported to one decimal place and standard deviations reported to two decimal places, or according to the journal's preferred format).

**********

7. PLOS authors have the option to publish the peer review history of their article (what does this mean?). If published, this will include your full peer review and any attached files.

If you choose “no”, your identity will remain anonymous but your review may still be made public.

**Do you want your identity to be public for this peer review?**  For information about this choice, including consent withdrawal, please see our Privacy Policy.

Reviewer #2: **Yes:**  Kelvin Oruko

**********

# Editor report: Acceptance letter

**DOI:** 10.1371/journal.pone.0353677.r008

**Authors:** Stanisław Wroński, Stanisław Wroński, Stanisław Wroński, Stanisław Wroński

PONE-D-25-64830R3

PLOS One

Dear Dr. Touma Sawaya,

I'm pleased to inform you that your manuscript has been deemed suitable for publication in PLOS One. Congratulations! Your manuscript is now being handed over to our production team.

At this stage, our production department will prepare your paper for publication. This includes ensuring the following:

* All references, tables, and figures are properly cited

* All relevant supporting information is included in the manuscript submission,

* There are no issues that prevent the paper from being properly typeset

You will receive further instructions from the production team, including instructions on how to review your proof when it is ready. Please keep in mind that we are working through a large volume of accepted articles, so please give us a few days to review your paper and let you know the next and final steps.

Lastly, if your institution or institutions have a press office, please let them know about your upcoming paper now to help maximize its impact. If they'll be preparing press materials, please inform our press team within the next 48 hours. Your manuscript will remain under strict press embargo until 2 pm Eastern Time on the date of publication. For more information, please contact onepress@plos.org.

You will receive an invoice from PLOS for your publication fee after your manuscript has reached the completed accept phase. If you receive an email requesting payment before acceptance or for any other service, this may be a phishing scheme. Learn how to identify phishing emails and protect your accounts at https://explore.plos.org/phishing.

If we can help with anything else, please email us at customercare@plos.org.

Thank you for submitting your work to PLOS ONE and supporting open access.

Kind regards,

PLOS ONE Editorial Office Staff

on behalf of

Dr. Stanisław Jacek Wroński

Academic Editor

PLOS One
